# Evaluating anthelmintic, anti-platelet, and anti-coagulant activities, and identifying the bioactive phytochemicals of *Amaranthus blitum* L.

**DOI:** 10.1186/s12906-024-04478-2

**Published:** 2024-05-04

**Authors:** Ghada Abdel-Moez, Hanaa Sayed, Azza Khalifa, Salwa Abd-Elrahman, Mohammed Osman, Shaymaa Mohamed

**Affiliations:** 1 Department of Pharmacognosy, Faculty of Pharmacy, Assiut University, Assiut, 71526 Egypt; 2 Department of Parasitology, Faculty of Veterinary Medicine, Assiut University, Assiut, 71515 Egypt; 3 Department of Clinical Pathology, Faculty of Medicine, Assiut University, Assiut, 71511 Egypt

**Keywords:** *Amaranthus blitum* L., Amaranthaceae, Anthelmintic, Haemostatic, Anti-coagulant, Anti-platelet

## Abstract

**Background:**

Highlighting affordable alternative crops that are rich in bioactive phytoconstituents is essential for advancing nutrition and ensuring food security. *Amaranthus blitum* L. (AB) stands out as one such crop with a traditional history of being used to treat intestinal disorders, roundworm infections, and hemorrhage. This study aimed to evaluate the anthelmintic and hematologic activities across various extracts of AB and investigate the phytoconstituents responsible for these activities.

**Methods:**

In vitro anthelmintic activity against *Trichinella spiralis* was evaluated in terms of larval viability reduction. The anti-platelet activities were assessed based on the inhibitory effect against induced platelet aggregation. Further, effects on the extrinsic pathway, the intrinsic pathway, and the ultimate common stage of blood coagulation, were monitored through measuring blood coagulation parameters: prothrombin time (PT), activated partial thromboplastin time (aPTT), and thrombin time (TT), respectively. The structures of isolated compounds were elucidated by spectroscopic analysis.

**Results:**

Interestingly, a previously undescribed compound (**19**), *N*-(*cis*-*p*-coumaroyl)-ʟ-tryptophan, was isolated and identified along with 21 known compounds. Significant in vitro larvicidal activities were demonstrated by the investigated AB extracts at 1 mg/mL. Among tested compounds, compound **18** (rutin) displayed the highest larvicidal activity. Moreover, compounds **19** and **20** (*N*-(*trans*-*p*-coumaroyl)-ʟ-tryptophan) induced complete larval death within 48 h. The crude extract exhibited the minimal platelet aggregation of 43.42 ± 11.69%, compared with 76.22 ± 14.34% in the control plasma. Additionally, the crude extract and two compounds **19** and **20** significantly inhibited the extrinsic coagulation pathway.

**Conclusions:**

These findings extend awareness about the nutritional value of AB as a food, with thrombosis-preventing capabilities and introducing a promising source for new anthelmintic and anticoagulant agents.

**Supplementary Information:**

The online version contains supplementary material available at 10.1186/s12906-024-04478-2.

## Background

The genus *Amaranthus* (Amaranthaceae) encompasses approximately 70 species, and among them, *Amaranthus blitum* L. (AB) stands out for its nutritional properties and traditional uses. AB has a rich history of traditional use in treating intestinal disorders, hemorrhage, and roundworms [1]. AB has tender leaves and is rich in flavor. Despite sounding popular as food in many African, Asian, and South American countries, AB in Egypt is considered a weed rather than superfood opportunity. A previous study, that has investigated 15 *Amaranthus* species, showed that AB had appreciated levels of minerals, vitamin C, phenols, and flavonoids [2]. Concerning phytochemical composition, antioxidant betalains, betaxanthins, and anthocyanins pigments were estimated in AB extract [3]. In addition, some nonpolar constituents, including fatty acids and fatty esters were identified from the AB methanolic extract through GC-MS analysis [4]. In our earlier study, a total of 21 compounds were identified in the LC-MS/MS analysis of ethanolic extract of AB aerial parts [5]. Considering these limited phytochemical investigations, the current study aimed at expanding the chemical profile of this plant and isolating its main constituents.

Trichinosis is a helminth disease caused by *Trichinella spiralis* nematode (*T. spiralis*). The main transmission route of *T. spiralis*, which has infected approximately 11 million people worldwide, is by accidental ingestion of raw or undercooked meat from infected animals [6]. *T. spiralis* was one of the top 10 most prevalent foodborne parasites, according to the World Health Organization (WHO), that might eventually cause major health issues. To validate the traditional claim of AB effectiveness for treating round worms, we investigated the anthelmintic properties of the plant extracts and selected isolated compounds against *T. spiralis* ML in a trial to identify the effective phytoconstituents.

Restoring an efficient hemostatic system is vital for patients with hematologic abnormalities. Several cardiovascular diseases and inflammatory conditions are associated with pathological thrombosis, which is characterized by activated platelet aggregation and coagulation [7, 8]. Thereby, anti-platelet and anticoagulant agents are valuable therapeutics for these pathologies. A coagulation cascade involves the intrinsic and extrinsic pathways which originate differently but converge at the point of fibrin activation and acts to stabilize the platelet plug [9]. In this context, a protein of a related species, *Amaranthus hypocondriacus*, has demonstrated antiplatelet and antithrombotic activities [10]. Taken together, this study sought to investigate the effect of AB extracts and certain isolates on the hemostatic system.

## Materials and methods

### Ethics statement

All experimental procedures were carried out according to the principles and guidelines of the Ethics Committee of Faculty of Medicine, Assiut University, Egypt, for biological studies, with an approval number 17101912.

### General phytochemical experimental procedures

The ^1^H, APT, and 2D NMR experiments were run at 400 and 100 MHz using Bruker Avance III spectrometer, Faculty of Science, Zagazig University. The HR-ESI-MS analysis was performed using a Bruker Bioapex-FTMS with electrospray ionization (USA). Chromatographic adsorbents include silica gel G_60_ (60–120 mesh, Merck, Darmstadt, Germany), C18-RP silica gel (230–400 mesh, Merck, Darmstadt, Germany), MN-polyamide-SC-6 (50–160 μm, Sorbent Technologies, Norcross, GA, USA), Sephadex^®^ LH-20 (Mitsubishi Kagaku, Tokyo, Japan), Florosil (60–100 mesh, Carlo Erba Reagents, France) and Diaion^®^ HP-20 (Sorbent Technologies, Norcross, GA, USA). TLC was conducted on pre-coated silica 60 F254 aluminum sheets, 0.25 mm and RP-18 F254, 0.25 mm (E-Merck), Darmstadt, Germany). Solvents used for extraction and isolation were of analytical grade, purchased from (Adwic - El Nasr Pharmaceutical Co., Cairo, Egypt). Deuterated solvents for NMR spectral analysis: DMSO-*d*_6_, CD_3_OD, CDCl_3_, C_5_D_5_N (Cambridge Isotope Laboratories, Inc., MA).

### Plant material

Livid amaranth (*Amaranthus blitum* L.) is an annual herbaceous vegetable belonging to the Amaranthaceae family (https://www.worldfloraonline.org/taxon/wfo-0000530085). The aerial parts of AB were collected during the flowering season in September 2020 from The Medicinal Plants Station, Pharmacognosy Department, Assiut University, Assiut, Egypt. The plant was authenticated by Dr. Mostafa Aboelela, Associate Professor of Plant Taxonomy, Botany Department, Faculty of Science, Assiut University. A voucher herbarium specimen (No. 0002017) was deposited in the Pharmacognosy Department Museum, Faculty of Pharmacy, Assiut University. The collected materials were air-dried, powdered, and kept until subsequent research.

### Extraction and fractionation

The powdered plant material (3.6 kg) was macerated in 70% ethanol (5 × 6 L). The combined extracts were vacuum concentrated at 40 °C to give a residue of 248 g. This residue was suspended in distilled water (600 mL) and partitioned with *n*-hexane (8 × 1 L). The combined *n*-hexane extracts were vacuum concentrated to obtain the *n*-hexane fraction (104 g). The remaining aqueous layer was concentrated to a dried residue (140 g). The dried aqueous residue was subjected to Diaion-HP20 (500 g) column chromatography (CC), eluted with 100% H_2_O (3 L), followed by 100% MeOH (3 L). These eluents were vacuum concentrated to yield two fractions of 125 g and 10 g, respectively. The latter fraction, weighing 10 g, will be referred to as the polar fraction. An illustrative scheme was provided to depict the process (Supplementary scheme S1).

### Isolation of compounds 1–6 from *n*-hexane fraction

A portion of *n*-hexane fraction (90 g) was fractionated into five fractions labelled as (F1–F5), using vacuum liquid chromatography. The elution was carried out using a combination of *n*-hexane (*n*-Hex) and acetone (Ace) as solvent systems, with a gradient of increasing proportions of acetone. The step-by-step procedures for isolating compounds **1**–**6** from these fractions, along with an illustrative scheme of the process, can be found in (Supplementary S1 and scheme S2).

### Isolation of compounds 7–22 from the polar fraction

**Fig. 1:**
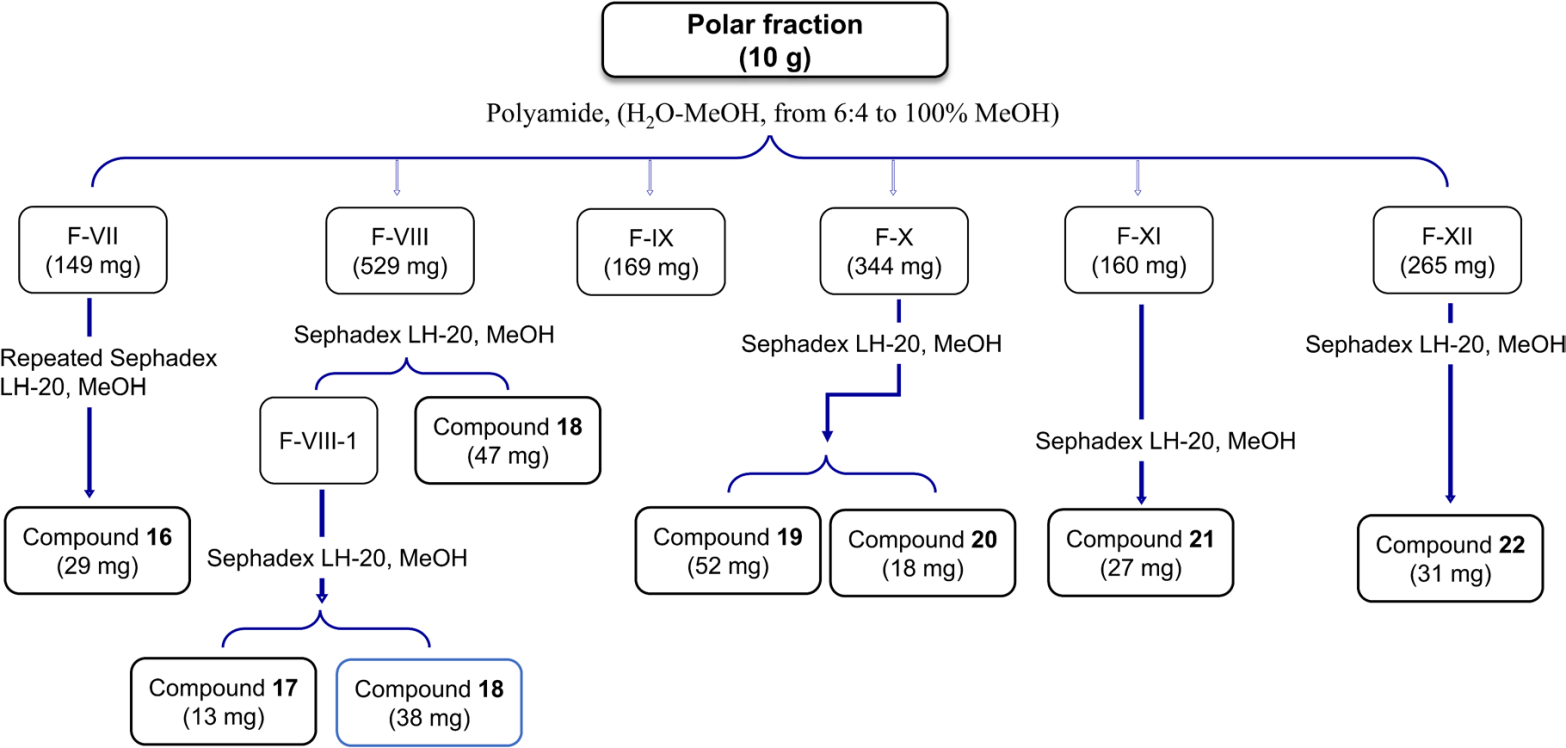
Isolation and purification of compounds **16**–**22** from the polar fraction of *Amaranthus blitum* L.

The polar fraction (10 g) was fractionated into 12 subfractions (F-I to F-XII), using polyamide column chromatography (250 g). The elution was carried out using water (H_2_O) initially, followed by gradient solvent systems of H_2_O and methanol (MeOH). The details for isolating compounds **7**–**22** from these subfractions, along with corresponding illustrative schemes of the process, can be found in (Supplementary S2, Scheme S3, and Fig. 1).

### In vitro anthelmintic effects

The anthelmintic efficacy of AB extracts and selected pure isolates were evaluated, for the first time, against *Trichinella spiralis* muscular larvae, in comparison with albendazole (ABZ) as a standard. The larvae were recovered from experimental *Trichinella spiralis*-infected albino mice and prepared using the procedure described by [11]. This investigation included three extracts (crude ethanolic extract, *n*-hexane fraction, and the polar fraction) at two concentrations of 0.5 and 1 mg/mL, and four compounds (**2** and **18**–**20**), at concentrations of (6.25, 12.5, 25, and 50 μg/mL), compared with ABZ (20 μg/mL). The treated larvae were inspected under a light microscope (Leica^®^, Germany) to monitor the viability rates at successive time points. Besides, scanning electron microscope (JEOL model, JEM-100 CXII, Tokyo, Japan) was used to examine the morphological alterations associated with different treatments.

#### Observation of muscular larvae viability using light microscopy

Using a sterile 6-well plates, three wells were dedicated for each concentration. In accordance with the described protocol [11], *T. spiralis* muscular larvae (ML) was diluted in Rapid Prototyping and Manufacturing Institute (RPMI)-1640 medium. A larval suspension (50 μL) of approximately 100 larvae was added to 1 mL of each sample. The 6 well plates were then sealed and incubated at 37 °C in an atmosphere containing 5% CO_2_ at different time points of 1, 4, 24, 48, and 72 h. At the end of the incubation periods, the number of viable ML, those that survive the applied treatment, was determined by direct observation under the light microscope, estimated by their motility. The results were expressed as the viability percentage. Besides, phenotypic changes induced by different treatments were observed at different time-points using light microscope.

#### Scanning electron microscopy (SEM)

To thoroughly detect any change in morphological features, an SEM examination of exposed larvae was undertaken. Certain specimens, that induced noteworthy phenotypic changes when examined under a light microscope, were chosen including, the three extracts at 0.5 mg/mL, compounds **2** and **18** at 50 μg/mL, compound **19** at 25 μg/mL, and compound **20** at 12.5 μg/mL. A typical protocol was followed for preparing parasite specimens [11]. After being properly washed in PBS, larvae were fixed for 2 h with 2.5% glutaric dialdehyde. The larvae were rewashed in PBS, post-fixed in a 2% osmium tetroxide in sodium cacodylate buffer for 1 h. Afterward, dehydration of postfixed worms was done in alcohol series of increasing concentrations, followed by air-drying, mounting on a stub, and gold-coating. Eventually, high resolution graphs were generated for parasites using a scanning electron microscope.

### Evaluation of the in vitro hematological activity

Blood samples were obtained from freshly donated blood bags at Assiut university hospital, central blood bank. In addition to the typical procedures for selection of donors applied at the blood bank, meticulous drug history was obtained from the donors to exclude any possibility of test results alteration due to drugs. CPDA-1 (Citrate phosphate dextrose adenine) was the anticoagulant utilized in blood bags. Fifteen mL from each bag were withdrawn into plain plastic tubes without any additional anticoagulant.

#### Preparation of platelet rich plasma (PRP) and platelet poor plasma (PPP)

At the beginning of the procedure, blood samples were placed in a centrifuge and spun at 1000 rpm (RPM) for 10 minutes. This step aimed to obtain a three-layered solution. The upper layer, known as plasma, was carefully examined for any remaining erythrocytes. If necessary, the samples underwent an additional centrifugation for 5 minutes at the same speed. During this process, the superficial layer containing platelets was extracted with caution, ensuring no disturbance to the intermediate buffy layer or underlying erythrocytes. This extracted layer is referred to as Platelet-Rich Plasma (PRP). To prepare Platelet-Poor Plasma (PPP), the PRP obtained previously underwent a second centrifugation at 2500 RPM for 20 minutes. This step caused the platelets to precipitate as pellets. The resulting supernatant, known as PPP, was carefully examined for hemolysis and then transferred into sterile tubes. By gently shaking the platelet pellets with a small amount of plasma, PRP is formed [12].

#### Light transmission aggregometry

The effect of AB extracts and compounds **2**, **19**, and **20** on platelet aggregation in PRP samples was evaluated, within 1 h of blood samples withdrawal, using a light transmission aggregometry. This technique relies on measuring the passage of light through turbid PRP, which contains uniformly circulating platelets. The addition of an agonist causes platelets to aggregate, resulting in a clearer, less light-absorbing solution. We used two common platelet agonists, ADP and AA, to activate the platelets. The light transmittance is detected using a photocell. The method involves passing an infrared beam through two cuvettes: one filled with platelet-poor plasma (PPP) as a reference and the other containing the PRP sample being analyzed. The resulting signals represent the continuous difference in light transmittance between the analyzed and reference samples. Prior to measurement, the PRP samples (495 μL) were pre-incubated with the examined plant extracts (5 μL) for 15 minutes at 37 °C. Subsequently, the samples were transferred into aggregometer cuvettes. Platelet aggregation was measured after stimulation with ADP or AA using concentrations of 10 μmol/L for ADP and 0.5 mg/mL for AA. Stock solutions of the AB extracts and compounds were prepared using 30% DMSO. The level of aggregation was expressed as a percentage of aggregation [13].

#### Measurement of the haemostatic parameters

In this assay, we measured the blood clotting times using automated Sysmex coagulation analyzers. The clotting times tested included prothrombin time (PT), activated partial thromboplastin time (aPTT), and thrombin time (TT). Before conducting the assay, plasma samples were pre-incubated with the tested extracts and compounds at 37 °C for 20 minutes. The final concentrations used for the crude ethanolic extract, *n*-hexane fraction, and polar fraction were 50 μg/mL. For compounds **2**, **19**, **20**, and dabigatran (reference anticoagulant drug), the concentrations used were 5 μg/mL [14].

### Statistical analysis

SPSS (Statistical Package for the Social Science, version 20, IBM, and Armonk, New York) was used for statistical analysis. Data were gathered and expressed as the mean ± SD. Level of confidence was kept at 95% and hence, *p value ≤ 0.05 was considered statistically significant while **p value ≤ 0.01 was considered highly statistically significant*.* For significant difference between dependent multiple groups at different time points, Kruskal-Wallis test was used. For significant difference between the mean values of independent groups compared with the control group, multiple t-test and student t-test were applied.

## Results

### Phytochemical investigation

**Fig. 2:**
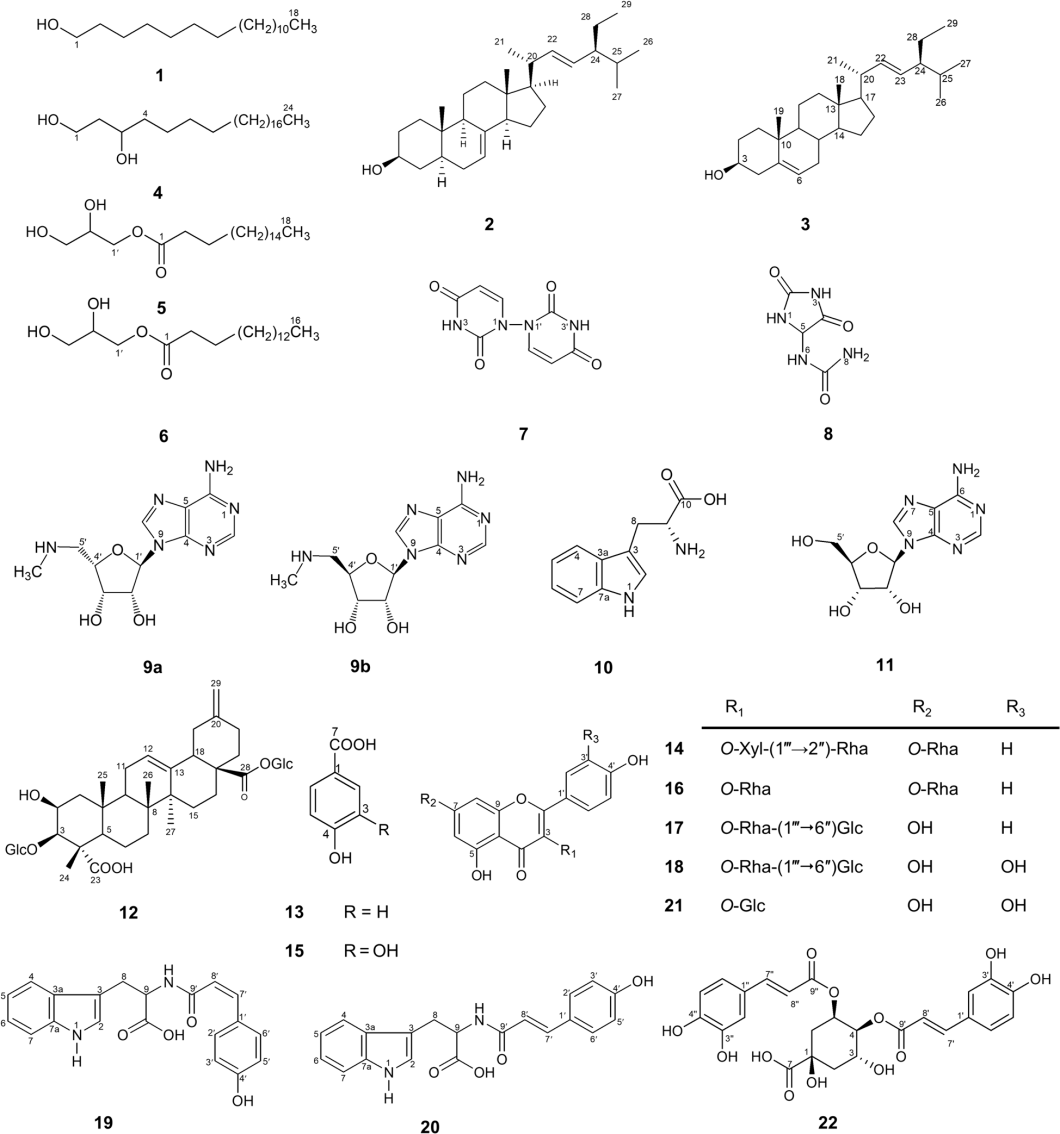
The chemical structures of the isolated compounds

The chromatographic and spectral analysis (Supplementary Fig. S1–S73) resulted in isolation and identification of a previously undescribed compound (**19**) along with other diverse 21 known compounds (Fig. 2 and Table 1). Structures of known compounds (**1**–**18** and **20**–**22**) were elucidated as 1-octadecanol (**1**) [15], α-spinasterol (**2**) [16], stigmasterol (**3**) [17], 1,3-tetracosanediol (**4**) [18], glycerol monostearate (**5**) [19], glycerol monopalmitate (**6**) [20], 1,1′-biuracil (**7**) [21], *S*-allantoin (**8**) [22], 5′-deoxy-5′-(methylamino)adenosine (**9a**) [23], 5′-deoxy-5′-(methylamino)-9-(α-ʟ-lyxofuranosyl)-adenine (**9b**) [24], ʟ-tryptophan (**10**) [17], adenosine (**11**) [25], 3-*O*-β-d-glucopyranosyl-2β,3β-dihydroxy-30-noroleane-12,20(29)-diene-23,28-dioic acid 28-*O*-β-d-glucopyranosyl ester (**12**) [26], *p*-hydroxybenzoic acid (**13**) [27], sagittatin A (**14**) [28], protocatechuic acid (**15**) [29], kaempferitrin (**16**) [28], nicotiflorin (**17**) [30], rutin (**18**) [30], *N*-(*E*-*p*-coumaroyl)-ʟ-tryptophan (**20**) [31], isoquercetin (**21**) [30], 4,5-*O*-dicaffeoylquinic acid (**22**) [32].

**Table 1:** ^1^H and ^13^C (APT) NMR spectral data of compounds **19** and **20** (CD_3_OD, 400, 100 MHz)

Position	*δ*_H_ (ppm), *multiplicity*, *J* (Hz)	*δ*_C_ (ppm)	*δ*_H_ (ppm), *multiplicity*, *J* (Hz)	*δ*_C_ (ppm)
	Compound **19**		Compound **20**	
	ʟ-Tryptophan moiety			
1-NH	10.12, *br s*	–	10.20, *br s*	–
2	7.01, *s*	124.4	7.11, *s*	124.4
3	–	111.4	–	111.4
3a	–	129.0	–	129.1
4	7.56, (*d*, 8)	119.4	7.59, (*d*, 8)	119.5
5	6.96, (*td*, 8, 1.2)	119.6	6.97, (*td*, 7.4, 1.1)	119.7
6	7.06, (*td*, 8, 1.2)	122.2	7.05, (*td*, 7.6, 1.1)	122.2
7	7.31, (d, 8)	112.2	7.30, (*d*, 8)	112.1
7a	–	137.9	–	137.9
8	Ha (3.19, *dd*, 7.2, 14.4) Hb (3.38, *d*, 4.8)	28.8	Ha (3.25, *dd*, 7.2, 14.5) Hb (3.42, *dd*, 4.7, 14.8)	28.9
9	4.74, (*t*, 7.2)	55.8	4.82, *br s*	55.9
9-COOH	–	177.0	–	177.0
	*trans*-*p*-Coumaroyl moiety		*cis*-*p*-Coumaroyl moiety	
1′	–	127.8	–	127.7
2′, 6′	7.27, (*d*, 8.8)	132.3	7.35, (*d*, 8.7)	130.6
3′, 5′	6.61, (*d*, 8.8)	115.9	6.77, (*d*, 8.7)	116.7
4′	–	159.0	–	160.4
7′	6.54, (*d*, 12.8)	138.4	7.40, (*d*, 15.8)	142.0
8′	5.77, (*d*, 12.8)	121.1	6.42, (*d*, 15.8)	118.4
9′	–	169.6	–	168.8

### In vitro anthelmintic effects

#### Larvicidal activities and larval phenotypic changes

The untreated larvae, negative control (NC), appeared active and kept its typical motility (Supplementary Fig. S74). As shown in Table 2, The crude ethanolic extract showed a moderate larvicidal effect, gradually lowering the larval viability to 15% within 72 h at 1 mg/mL; however, the lower concentration (0.5 mg/mL) exhibited only weak activity. Larvae exposed to 1 mg/mL, manifested a phenotypic change to wide circles with slow motility after 24 h (Supplementary Fig. S75). The majority of ML were completely dead after 72 h, as judged by the distinct comma-shaped appearance (Supplementary Fig. S76). However, at 0.5 mg/ml, some treated larvae appeared somewhat contracted and had wide circle bodies, but the majority remained coiled and viable. Interestingly, the *n*-hexane fraction induced a strong fall in viability percentage to approximately 20% at 1 mg/mL within 24 h. Complete mortality was reached by 72 h. In contrast, high viability proportions were observed at 0.5 mg/mL, revealing no noticeable larvicidal activity. Despite recording high viability levels at 0.5 mg/mL, larvae appeared obviously contracted in incomplete circles (C-shaped) (Supplementary Fig. S77). Encouraging results were obtained by the polar fraction, demonstrating the highest larvicidal activity. Complete larval death was observed after 48 h at 1 mg/mL. At this high concentration, most larvae shaped as commas and others showed wide circles after 24 h. The effect of this treatment was increased with increasing the exposure time, with all ML dead within 48 h. At a lower concentration of 0.5 mg/mL, similar changes as those observed in case of *n*-hexane larvae were observed to be contracted, showing half circle appearance (C-shaped) after 24 h, some larvae were coiled, and others lost their contractility and exhibited wide circle appearance within 48 and 72 h.

**Table 2:** Results of in vitro larvicidal activity of different AB extracts and isolated compounds (**2**, **18**, **19**, and **20**) against *T. spiralis* in terms of larval viability percentage

Sample	Dose (mg/mL)	Measurement time-points					
		1 h	4 h	24 h	48 h	72 h	*p* value
Different AB extracts (mg/mL)							
Crude extract	0.5	100	100	92.7 ± 2.52^*^	85.0 ± 5.00	81.7 ± 7.64	0.018^*^
	1	100	100	61.7 ± 2.30^*^	35.0 ± 5.00^**^	15.0 ± 5.00^**^	0.012^*^
*n*-hexane fraction	0.5	100	100	84.3 ± 4.51^*^	80.0 ± 5.00	81.0 ± 5.57	0.032^*^
	1	100	100	21.7 ± 3.64^**^	15.7 ± 1.15^**^	3.0 ± 2.65^**^	0.014^*^
The polar fraction	0.5	100	97.7 ± 4.93	84.0 ± 3.61	80.3 ± 0.58	72.7 ± 2.52	0.014^*^
	1	100	95.3 ± 0.58	21.7 ± 7.64^**^	0^**^	0^**^	0.008^*^
Certain isolated compounds (μg/mL)							
**2**	6.25	100	100	95.0 ± 1.00	93.0 ± 1.00	88.3 ± 1.50	0.012^*^
	12.5	100	100	94.0 ± 1.50	91.7 ± 1.50	83.0 ± 2.60	0.012^*^
	25	100	100	90.0 ± 1.00	87.0 ± 0.60^*^	82.3 ± 2.50^*^	0.015^*^
	50	100	97.7 ± 2.50	40.0 ± 1.00^**^	25.0 ± 1.00^**^	15.0 ± 5.00^**^	0.017^*^
**18**	6.25	100	100	92.3 ± 2.10^*^	90.3 ± 0.60	92.0 ± 1.00	0.023^*^
	12.5	100	100	91.0 ± 1.00^**^	89.7 ± 1.50	81.0 ± 1.00	0.014^*^
	25	100	100	87.7 ± 2.50^**^	86.0 ± 1.00	81.0 ± 1.00	0.015^*^
	50	97.7 ± 2.50	85.0 ± 5.00^*^	48.3 ± 5.4^**^	0^**^	0^**^	0.011^*^
**19**	6.25	100	100	96.0 ± 1.00^*^	94.0 ± 1.00	89.0 ± 1.00	0.012^*^
	12.5	100	100	94.7 ± 1.50^*^	93.3 ± 0.60	87.0 ± 1.00	0.014^*^
	25	100	100	93.7 ± 1.50^*^	89.7 ± 0.60	88.0 ± 1.00	0.012^*^
	50	100	96.7 ± 2.10	20.0 ± 5.00^**^	0^**^	0^**^	0.009^**^
**20**	6.25	100	100	96.0 ± 1.00^*^	94.3 ± 1.00	86.0 ± 1.00	0.012^*^
	12.5	100	100	94.7 ± 0.60^**^	92.0 ± 1.00	86.0 ± 1.00	0.012^*^
	25	100	100	91.7 ± 1.50^**^	88.3 ± 1.50	84.7 ± 0.60	0.012^*^
	50	100	95.0 ± 1.00	12.3 ± 6.8^**^	0^**^	0^**^	0.008^**^
NC	–	100	100	99.0 ± 1.00	80.3 ± 1.53	76.0 ± 1.00	0.02^*^
ABZ	20 μg/mL	100	49.0 ± 3.61	0	0	0	0.008^**^

Data were expressed as the mean of triplicates ± standard deviation (SD)

Kruskal-Wallis test was used for analysis of significant difference between dependent multiple groups at different time points. For significant difference between the mean values of independent groups compared with the control group, multiple student t-test was applied

^*^p value ≤0.05 was considered statistically significant while ^**^p value ≤0.01 was considered highly statistically significant

Among the compounds studied, rutin (**18**) exhibited the highest larvicidal effects at a concentration of 50 μg/mL. It demonstrated rapid reduction in larval viability, reaching less than 50% within 24 h and complete eradication within 48 h.

Additionally, compound **20** induced complete larval mortality between 24 h and 48 h at the highest tested concentration, followed by compound **19**, which caused complete larval death by 48 h at 50 μg/mL.

Compound **2** induced a considerable decline in larval viability at successive time-points and killed the majority of ML after 72 h at 50 μg/mL. Dead larvae were observed after 24 h, in response to the treatment with compound **18** at 50 μg/mL. However, at 25 μg/ml, larvae were contracted, with no motility, showing C-shaped appearance after 24 h and became coiled, with complete disappearance of viability and contractility after 48 h. At 12.5 μg/ml, half of the larvae were contracted, while the remaining were coiled then the larvae began to lose their contractility and shaped as wide circles by 72 h. At 6.25 μg/mL, many larvae were supercoiled, larvae apparently immotile but they are totally viable and can contract and take a button shape appearance (Supplementary Fig. S78) and totally viable, this means that the effect of rutin on viability was dose dependent. Regarding compounds **19** and **20** (50 μg/mL), major proportion of larvae were comma shaped from the first 24 h, indicating complete death. At 25 and 12.5 μg/mL, associated phenotypic modifications were observed on larvae, which were contracted, with no apparent motility, showing C-shape bodies after 24 h and became coiled by 48 h. Moreover, some larvae were coiled, and others demonstrated as wide circles within 72 h. However, at a 6.25 μg/mL, the exposed larvae appeared normal; some were wrinkled, with low motility after 24 h and all larvae appeared as C-shape within 72 h. In the case of compound **2** (50 μg/mL), most larvae showed comma shaped appearance, viable ones appeared very week after 24 h then all larvae were dead after 72 h. At (25 and 12 μg/mL), the larvae were contracted, with low motility, whereas, at 6.25 μg/mL, some larvae were coiled.

#### Morphological alterations in larvae cuticles

**Fig. 3:**
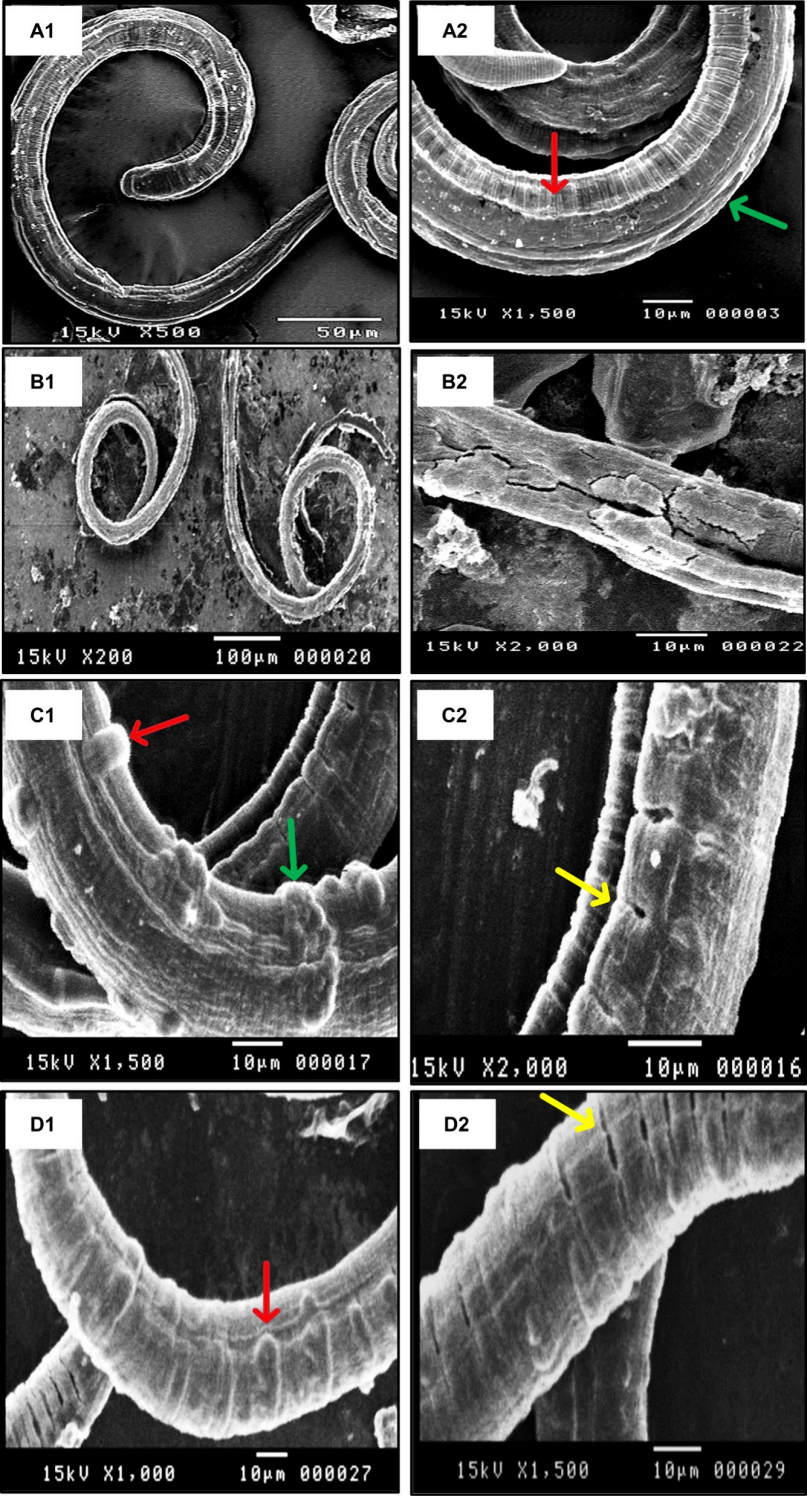
Scanning electron microscopy of *Trichinella spiralis* larva: (A1 and A2): Untreated larva showing normal cuticle with transverse creases (red arrow) and longitudinal ridges (green arrow). (B1 and B2): Compound **18**-treated larvae, with totally disintegrated bodies, showing sloughing and detachment of the cuticles. (C1 and C2): Compound **19**-treated larvae, showing pores (yellow arrow), severe blebbing (red arrow), and severe wart-like lesions (green arrow). (D1 and D2): Compound **20**-treated larvae, showing opacity and loss of striation, contracted larvae, severe lesion, pores (yellow arrow), and severe blebbing (red arrow)

SEM revealed remarkable morphological changes in cuticles of the treated larvae, contrasting that of untreated larvae with typical transverse creases and longitudinal ridges (Fig. 3, A1 and A2). The crude extract-treated larvae (0.5 mg/mL) became opaque with loss of normal striation, also there were some pores that may serve as penetration and entry points for the treatment to reach the interior of the larvae (Supplementary Fig. S79). In the case of *n*-hexane fraction-treated group (0.5 mg/mL), the larvae showed holes and some blebs on the cuticle (the blebbing is an attempt by the parasite to replace damaged surface membrane in response to drug) (Supplementary Fig. S80). While larvae treated with the polar fraction (0.5 mg/mL) and isolated compounds were more affected than other groups, there was a loss of normal striation, the larvae became opaque and shortened. Also, there were some pores, and the most obvious effect was sloughing of some areas and detachment of the cuticles (Supplementary Fig. S81). The larvae treated with compounds **18**–**20** showed remarkable destructive features. Sloughing of some areas and detachment of the cuticles of compound **18** (50 μg/mL)-treated larvae were observed (Fig. 3, B1 and B2). Blebbing and wart-like lesions (that may be hyper-atrophy at the tissue of the parasites) were obvious in compound **19** (25 μg/mL)-treated larvae (Fig. 3, C1 and C2). Additionally, presence of pores, sloughing, and blebbing were observed in the groups treated with compound **20** (12.5 μg/mL) (Fig. 3, D1 and D2). In a similar manner, compound **2**-treated larvae showed severe lesions as sloughing and detachment of the cuticle.

### Evaluation of the in vitro hematological activities

#### In vitro anti-platelet aggregating activity

Platelet aggregometry assay is generally used for the analysis of platelet function. In this assay, the primary hemostasis (human platelet aggregation), induced by two agonists, ADP and AA, was assessed in the presence of different investigated samples. Interestingly, the crude extract and the polar fraction displayed highly significant suppression (*p* < 0.001) in percentages of ADP- and AA-induced aggregation, compared with control plasma. As listed in Table 3, the maximum inhibitory potency was recorded with the crude ethanolic extract followed by the polar fraction. Whereas compounds **2**, **19**, and **20** showed less effective, but statistically significant, inhibitory activity against ADP- and AA-induced platelet aggregation, compared with the control plasma. In contrast, *n*-hexane fraction showed a non-significant reduction in platelet aggregation, compared with the control plasma.

**Table 3:** Platelet aggregation (%) in the presence of investigated samples, with ADP and AA as platelet agonists, ^*^p < 0.05, ^***^p < 0.001, *n* = 19

Sample	Platelet aggregation (%)	
	(ADP)	(AA)
Control plasma	75.11 ± 11.89	76.22 ± 14.34
Dabigatran	68.05 ± 10.71	71.84 ± 11.88
Crude extract	43.89 ± 15.25^***^	43.42 ± 11.69^***^
*n*-Hexane fraction	67.89 ± 13.14	69.53 ± 14.57
Polar fraction	45.15 ± 13.03^***^	46.89 ± 12.56^***^
Compound **2**	66.47 ± 15.38^*^	65.89 ± 13.36^*^
Compound **19**	66.42 ± 13.21^*^	65.53 ± 12.85^*^
Compound **20**	62.63 ± 14.13^*^	64.42 ± 13.53^*^

Student t test was used for analysis of platelet aggregation

Data were expressed as the mean ± standard deviation (SD)

### Effects on the coagulation cascade

#### Effects on prothrombin time (PT)

The crude extract and *n*-hexane fraction significantly prolonged PT. Among the studied compounds, compounds **19** and **20** exhibited highly significant prolongation of PT. In contrast, insignificant differences in PT were observed with the polar fraction and compound **2**, in comparison with the control plasma (Table 4).

**Table 4:** The mean prothrombin time (PT), activated partial thromboplastin time (aPTT), and thrombin time (TT) values in response to AB extracts and isolated compounds, ^*^p < 0.05, ^***^p < 0.001, *n* = 19

Sample	PT (s)	aPTT (s)	TT (s)
Control plasma	11.98 ± 0.65	36.51 ± 4.87	19.90 ± 1.38
Dabigatran	12.96 ± 0.73^*^	50.70 ± 9.31^***^	89.55 ± 1.58^***^
Crude extract	13.28 ± 1.11^***^	40.81 ± 4.94^*^	21.71 ± 1.35
*n*-Hexane fraction	13.88 ± 1.30^***^	40.14 ± 4.89	22.42 ± 1.92
Polar fraction	12.70 ± 1.18	38.51 ± 5.21	20.54 ± 1.74
Compound **2**	12.42 ± 0.90	45.99 ± 6.74^***^	20.72 ± 1.58
Compound **19**	15.96 ± 1.93^***^	37.65 ± 4.02	20.84 ± 2.05
Compound **20**	15.64 ± 2.86^***^	38.82 ± 4.14	20.93 ± 1.65

Student t test was used for analysis of clotting times

Data were expressed as the mean ± standard deviation (SD)

#### Effects on the activated partial thromboplastin time (aPTT)

Regarding the aPTT assay, the crude extract exhibited a mild increase in aPTT. Conversely, when the blood plasma sample was treated with compound **2**, a significant increase in aPTT time was observed. On the other hand, the remaining samples displayed negligible differences in aPTT compared to the control plasma (Table 4).

#### Effects on thrombin time (TT)

All investigated samples showed no significant difference in TT, compared with the control plasma as shown in (Table 4). However, Dabigatran, the used reference anticoagulant drug, prolonged all clotting times through thrombin inhibition.

## Discussion

The molecular formula of compound **19** was established to be C_20_H_18_N_2_O_4_, based on HR-ESI-MS data in positive-ion mode (Supplementary Fig. S1), which showed a molecular ion peak at *m/z* 351.1313 [M + H]^+^ (calcd for C_20_H_19_N_2_O_4_ 351.1344) and *m/z* 373.1120 [M + Na]^+^ (calcd for C_20_H_18_N_2_O_4_Na 373.1162) and confirmed via the negative-ion mode (Supplementary Fig. S2), which displayed the quasi-molecular ion peak at *m/z* 349.1186 [M − H]^−^ (calcd for C_20_H_17_N_2_O_4_ 349.1188) and *m/z* 699.2446 [2 M − H]^−^ (calcd for C_40_H_35_N_4_O_8_ 699.2455).

The ^1^H NMR spectrum (Supplementary Fig. S3) showed signals for two moieties, ʟ-tryptophan and *p*-coumaric acid, conjugated through an amide linkage. The tryptophan moiety was characterized by signals of indole ring and alanyl groups. Specifically, characteristic aromatic splitting pattern for the 3-substituted indole was observed, including two *o*-coupled doublets resonating at *δ*_H_ 7.56 and 7.31 attributed to H-4 and H-7, respectively, and two triplets of doublets at *δ*_H_ 6.96 and 7.06 attributed to H-5 and H-6, respectively (Table 1). Also, there were two singlets at *δ*_H_ 7.01 and 10.12, assigned for H-2 and 1-NH. The alanyl side chain was evidenced by two signals for the β methylene at *δ*_H_ 3.38 (*d*, *J* = 4.8 Hz) and 3.19 (*dd*, *J* = 14.4, 7.2 Hz); and an α-proton at *δ*_H_ 4.74. The *p*-coumaroyl group was revealed through the two sets of *o*-coupled aromatic protons of *p*-di-substituted benzene ring (AA′BB′ spin system) at *δ*_H_ 6.61 and 7.27 (each 2H, *d*, *J* = 8.8 Hz). In addition, signals for olefinic protons of the propenyl group were resonating at *δ*_H_ 5.77 (*d*, *J* = 12.8 Hz, H-8′) and 6.54 ppm (*d*, *J* = 12.8 Hz, H-7′). The double bond geometric isomerization was deduced as *Z*-configuration from the magnitude of coupling constant (12.8 Hz) between these protons. The APT spectrum of 19 (Supplementary Fig. S4) supported this analysis, displaying closely related, but chemically shifted, carbons than those observed for the known *trans* isomer, *N*-(*E*-*p*-coumaroyl)-ʟ-tryptophan (20), shortened as javamide-I. Based on careful 1D and 2D spectral analysis (Supplementary Fig. S5 and S6), 19 was assigned as *N*-(*Z*-*p*-coumaroyl)-ʟ-tryptophan, a newly described isomer for javamide I, which has never been reported so far. Interestingly, *N*-phenylpropenoyl-ʟ-amino acid conjugates have shown interesting biological activities. These conjugates have been detected in a few families, including Rubiaceae, Malvaceae, Asteraceae, and Apiaceae [31, 33, 34]. Javamide-I was previously isolated from green coffee beans [35]; whereas this is the first report for its isolation from the family Amaranthaceae. Structures of known compounds (**1**–**18** and **20**–**22**) were confirmed through comparison of their spectral data with the reported ones (Supplementary Fig. S7–S73). Among isolates, compounds **2**, **3**, **12**, **13**, **15**, **17**, **18**, and **21** were isolated from the plant for the first time; compounds **8** and **22** were first isolated from the genus; and compounds **1**, **4**–**7**, **9**–**11**, **14**, **16**, and **20** were first isolated from the family. These findings offer valuable chemotaxonomic information at species, genus, and family levels.

The anthelmintic investigation revealed, for the first time, significant in vitro dose- and time-dependent larvicidal activities of both *n*-hexane and polar fractions against *Trichinella spiralis* ML at 1 mg/mL and that even sublethal concentrations induced morphological changes of the larval musculature and could impair the larval motility. The polar fraction demonstrated the highest larvicidal efficacy among tested extracts. These findings were consistent with the detected activities of compounds **18**–**20**, isolated with high yields, from this polar fraction. Thus, the chemical composition of the polar fraction contributes to its superior larvicidal activity. Since compound **2** (spinasterol) and compound **18** (rutin) were major constituents of these bioactive fractions, they were considered in the anthelmintic investigation. Noteworthy, the selection of compounds **19** and **20**, members of *N*-phenylpropenoyl-ʟ-amino acid amides, to be investigated was justified considering the prevalence of indole heterocycles in the structures of many anthelmintics and the lack of studies investigating the anthelmintic activity of this interesting class [36, 37]. Additionally, it is of interest to investigate the influence of different geometrical stereochemistry of these isomers on their anthelmintic activity. Interestingly, significant in vitro larvicidal efficacies of the tested compounds at 50 μg/mL were reported in this study for the first time. Rutin displayed the highest larvicidal activity, with a rapid decrease in viability percentage. Moreover, the *N*-phenylpropenoyl-ʟ-amino acid amides, compounds **20** and **19**, induced complete larval mortality within 48 h. This further supports the existing evidence of the anthelmintic activities commonly observed in indole-based compounds. The significant anthelmintic activity of rutin reported herein is consistent with a recent study, which reported rutin’s anthelmintic effect against *Gastrothylax crumenifer* [38]. In addition, rutin displayed significant ovicidal and larvicidal activity against *Haemonchus contortus*, attributed to the presence of hydroxyl and rutinose groups [39]. Moreover, rutin sensitivity has also been observed in other nematodes, such as *Trichostrongylus*, *Chabertia*, and *Teladorsagia* [40]. The anthelmintic activities observed with compounds **19** and **20** are consistent with previous studies that have reported anthelmintic activity of such structural entity.

Regarding the hematological activities, rutin (compound **18**) was excluded from this investigation. This exclusion was based on its previous testing, which demonstrated anticoagulant activity by prolonging the activated partial thromboplastin time (aPTT) [41]. The observed highly significant anti-platelet aggregating activities of the crude extract and the polar fraction could be mediated by inhibition of thromboxane A2 release, or interference with arachidonic acid metabolism, leading to the inhibition of the fibrinogen binding to activated platelets and decreased platelet aggregation capacity [42]. These promising activities of the crude extract and the polar fraction might be attributed to their phytochemical profiles and could indicate the probable protective and therapeutic potential of the plant under investigation in cardiovascular disorders such as myocardial infarction.

The PT monitors the extrinsic and common pathways. In general, prolongation of PT may be associated with interference in some extrinsic factors or factor VII inhibition [42]. The highly significant prolongation of PT via compounds **19** and **20**, suggesting the potential anticoagulant activities of these *N*-phenylpropenoyl-ʟ-amino acid amides. This anti-coagulant effect can be exploited to prevent the formation of thrombi in the veins or arteries or the growing of such thrombi circulating in the bloodstream. The aPTT specifically measures the activity of the intrinsic system and common pathway of the coagulation system. Increased aPTT has been associated with Lupus anti-coagulant (antiphospholipid syndrome), heparin exposure and haemophilia A and B (Factor VIII and IX deficiency, respectively) [43]. Accordingly, possible adverse events in such patients and potential interactions with heparin should be considered in the future studies on this investigated compound **2** (spinasterol). The TT measures the ultimate step in the blood clotting cascade, in which fibrinogen is converted to fibrin.

## Conclusions

The phytochemical investigation of AB identified a new conjugate and other diverse phytoconstituents, most of them have proven health-promoting attributes. These findings spotlight the value of expanding the cultivation and economic exploitation of this species. The polar fraction demonstrated the highest larvicidal efficacy among tested extracts. Rutin (**18**) displayed the highest larvicidal activity, with a rapid decrease in viability percentage, which was less than 50% after 24 h, then dropped to zero within 48 h. Moreover, the *N*-phenylpropenoyl-ʟ-amino acid amides, compounds **19** and **20**, induced complete larval mortality within 48 h, supporting the anthelmintic activities documented for most indole-based compounds. The crude ethanolic extract and the polar fraction demonstrated potent anti-platelet activities, significantly lowering both ADP- and AA-induced platelet aggregation. These findings established the plant’s therapeutic possibility of preventing arterial thrombosis, myocardial infarction, and stroke. Based on blood clotting times analysis: The crude extract significantly prolonged PT and slightly prolonged aPTT, reflecting its influence on the extrinsic and common pathway. Compounds **19** and **20** (*N*-phenylpropenoyl-ʟ-amino acid amides) displayed significant prolongation of PT, indicating their inhibition capacities of the extrinsic coagulation pathway. These findings emphasize AB’s nutritional value, thrombosis-preventing potential, and its promise as a source for new anthelmintic and anticoagulant agents. While these in vitro findings are significant for early-stage research and provide valuable preliminary data, their relevance may be limited due to the lack of physiological factors such as immune responses, organ interactions, or systemic circulation. Therefore, further in vivo studies are necessary to gain a more complete understanding of the biological processes and their real-life significance.

### Supplementary Information

**Supplementary Material 1.**

## Data Availability

All data generated or analyzed during this study are included in this published article and its supplementary information file.

## Abbreviations

aPTT: Activated partial thromboplastin time
AA: Arachidonic acid
AB: *Amaranthus blitum* L.
ABZ: Albendazole
ADP: Adenosine diphosphate
C18-RP: C18-reversed-phase
C_5_D_5_N: Deuterated pyridine
CC: Column chromatography
CDCl_3_: Deuterated chloroform
CD_3_OD: Deuterated methanol
CPDA-1: Citrate phosphate dextrose adenine
DCM: Dichloromethane
DMSO-*d*_6_: Deuterated dimethyl sulfoxide
EtOAc: Ethyl acetate
GC-MS: Gas chromatography-mass spectrometry
H_2_O: Distilled water
LC-MS/MS: Liquid chromatography-tandem mass spectrometry
MeOH: Methanol
ML: Muscular larvae
PPP: Platelet-poor plasma
PRP: Platelet-rich plasma
PT: Prothrombin time
RPM: Revolutions per minute
RPMI: Rapid Prototyping and Manufacturing Institute
SEM: Scanning electron microscopy
SPE: Solid phase extraction
TT: Thrombin time
WHO: World Health Organization

## References

[1] Rastogi A, Shukla S (2013). Amaranth: A new millennium crop of nutraceutical values. Crit Rev Food Sci Nutr..

[2] Jiménez-Aguilar DM, Grusak MA (2017). Minerals, vitamin C, phenolics, flavonoids and antioxidant activity of *Amaranthus* leafy vegetables. J Food Compos Anal..

[3] Sarker U, Oba S (2020). Nutrients, minerals, pigments, phytochemicals, and radical scavenging activity in *Amaranthus blitum* leafy vegetables. Sci Rep..

[4] Jahan S, Nesa M, Hossain ME, Rajbangshi JC, Hossain MS (2022). In vivo and in silico evaluation of analgesic and hypoglycemic activities of *Amaranthus blitum* L. S Afr J Bot..

[5] Abdel-Moez G, Avula B, Sayed H, Khalifa A, Ross S, Katragunta K (2023). Phytochemical profiling of three *Amaranthus* species using LC-MS/MS metabolomic approach and chemometric tools. J Pharm Biomed Anal..

[6] Muñoz-Carrillo J, Maldonado-Tapia C, López- Luna A, Jesús Muñoz-Escobedo J, La Torre AF-D, J, Moreno-García A. Current aspects in Trichinellosis. In: Parasites and Parasitic Diseases. London: IntechOpen. 2019. p. 175–216.

[7] Stark K, Massberg S (2021). Interplay between inflammation and thrombosis in cardiovascular pathology. Nat Rev Cardiol..

[8] Ceulemans A, Spronk HMH, ten Cate H, van Zwam WH, van Oostenbrugge RJ, Nagy M (2024). Current and potentially novel antithrombotic treatment in acute ischemic stroke. Thromb Res..

[9] Davie EW (2003). A brief historical review of the waterfall/Cascade of blood coagulation. J Biol Chem..

[10] Sabbione AC, Rinaldi G, Añón MC, Scilingo AA (2016). Antithrombotic effects of *Amaranthus hypochondriacus* proteins in rats. Plant Foods Hum Nutr..

[11] Abd-Elrahman SM, Dyab AK, Mahmoud AES, Alsharif FM, Mohamed SM, Abomughaid MM (2021). Influence of chemically and biosynthesized silver nanoparticles on in vitro viability and infectivity of *Trichinella spiralis* muscle larvae. Ann Parasitol..

[12] Dhurat R, Sukesh M (2014). Principles and methods of preparation of platelet-rich plasma: A review and author′s perspective. J Cutan Aesthet Surg..

[13] Hakim NS, Papalois VE (2007). Surgical complications: diagnosis and treatment.

[14] Kolodziejczyk-Czepas J, Ponczek M, Sady-Janczak M, Pilarski R, Bukowska B (2021). Extracts from *Uncaria tomentosa* as antiplatelet agents and thrombin inhibitors – the in vitro and in silico study. J Ethnopharmacol..

[15] Azman NAN, Alhawarri MB, Rawa MSA, Dianita R, Gazzali AM, Nogawa T (2020). Potential anti-acetylcholinesterase activity of *Cassia timorensis* DC. Molecules..

[16] Lee MY, Shin IS, Kyoung H, Seo CS, Son JK, Shin HK (2014). α-Spinasterol from *Melandrium firmum* attenuates benign prostatic hyperplasia in a rat model. Mol Med Rep..

[17] Saeidnia S, Gohari A, Malmir M, Moradi A, Ajani Y (2011). Tryptophan and sterols from *Salvia limbata*. J Medic Plants..

[18] Schulz S (2001). Composition of the silk lipids of the spider *Nephila clavipes*. Lipids..

[19] Ng WS, Lee CS, Cheng SF, Chuah CH, Wong SF (2018). Biocompatible polyurethane scaffolds prepared from glycerol Monostearate-derived polyester polyol. J Polym Environ..

[20] Sun Y, ying, Meng K, Su Z xia, Guo G lin, Pu Y fang, Wang C hai. Isolation and purification of antialgal compounds from the red alga *Gracilaria lemaneiformis* for activity against common harmful red tide microalgae. Environ Sci Pollut Res. 2017;24:4964–72. 10.1007/s11356-016-8256-y 27995507

[21] Lee YG, Lee DG, Gwag JE, Kim M, Kim M, Kim HG (2019). A 1,1′-biuracil from *Epidermidibacterium keratini* EPI-7 shows anti-aging effects on human dermal fibroblasts. Appl Biol Chem..

[22] Chaniad P, Wattanapiromsakul C, Pianwanit S, Tewtrakul S (2016). Anti-HIV-1 integrase compounds from *Dioscorea bulbifera* and molecular docking study. Pharm Biol..

[23] Jiao L, Tao Y, Wang W, Mei L, Shao Y, Wang Q (2019). Chemical constituents of fruit body of *Armillaria luteo-virens*. Chem Nat Compd..

[24] Miao BJ, Chen J, Shao JH, Xu XQ, Zhao CC, Wang YP (2018). A new adenine glycoside from the flowers of *Brassica rapa*. Chem Nat Compd..

[25] Ciuffreda P, Casati S, Manzocchi A (2007). Spectral assignments and reference data complete ^1^H and ^13^C NMR spectral assignment of α-and β-adenosine, 2-deoxyadenosine and their acetate derivatives. Magn Reson Chem..

[26] Rastrelli L, Aquino R, Abdo S, Proto M, De Simone F, De Tommasi N (1998). Studies on the constituents of *Amaranthus caudatus* leaves: isolation and structure elucidation of new triterpenoid Saponins and Ionol-derived glycosides. J Agric Food Chem..

[27] El Euch IZ, Frese M, Sewald N, Smaoui S, Shaaban M, Mellouli L (2018). Bioactive secondary metabolites from new terrestrial Streptomyces sp. TN82 strain: isolation, structure elucidation and biological activity. Med Chem Res..

[28] Lin HY, Chang ST (2012). Kaempferol glycosides from the twigs of *Cinnamomum osmophloeum* and their nitric oxide production inhibitory activities. Carbohydr Res..

[29] Lin Z, Fang Y, Huang A, Chen L, Guo S, Chen J (2014). Chemical constituents from *Sedum aizoon* and their hemostatic activity. Pharm Biol..

[30] Hamed ANE, Abouelela ME, El Zowalaty AE, Badr MM, Abdelkader MSA (2022). Chemical constituents from *Carica papaya* Linn. Leaves as potential cytotoxic, EGFR wt and aromatase (CYP19A) inhibitors; a study supported by molecular docking. RSC Adv..

[31] Park JB. NMR confirmation and HPLC quantification of Javamide-I and Javamide-II in green coffee extract products available in the market. Int J Anal Chem. 2017;2017. 10.1155/2017/1927983 PMC5613680 29138635

[32] El-askary H, Salem HH, Motaal AA (2022). Potential mechanisms involved in the protective effect of Dicaffeoylquinic acids from *Artemisia annua* L. leaves against diabetes and its complications. Molecules..

[33] Hensel A, Deters AM, Müller C, Stark T, Wittschier N, Hofmann T (2007). Occurrence of *N*-phenylpropenoyl-ʟ-amino acid amides in different herbal drugs and their influence on human keratinocytes, on human liver cells and on adhesion of *helicobacter pylori* to the human stomach. Planta Med..

[34] Stark T, Hofmann T (2005). Isolation, structure determination, synthesis, and sensory activity of *N*-phenylpropenoyl-ʟ-amino acids from cocoa (*Theobroma cacao*). J Agric Food Chem..

[35] Murata M, Okada H, Homma S (1995). Hydroxycinnamic acid derivatives and *p*-Coumaroyl-(ʟ)-tryprophan, A novel Hydroxycinnamic acid derivative, from coffee beans. Biosci Biotechnol Biochem..

[36] Aderibigbe SA, Idowu SO, Olaniyi AA, Wright CW, Fatokun AA (2021). Bioactivity and cytotoxicity profiling of vincosamide and strictosamide, anthelmintic epimers from *Sarcocephalus latifolius* (smith) Bruce leaf: anthelmintic vincosamide and strictosamide from *S. Latifolius*. J Ethnopharmacol..

[37] Mondal P, Jana S, Balaji A, Ramakrishna R, Kanthal L (2012). Synthesis of some new isoxazoline derivatives of chalconised indoline 2-one as a potential analgesic, antibacterial and anthelmimtic agents. J Young Pharmacists..

[38] Minsakorn S, Watthanadirek A, Poolsawat N, Puttarak P, Chawengkirttikul R, Anuracpreeda P. The anthelmintic potentials of medicinal plant extracts and an isolated compound (rutin, C_27_H_30_O_16_) from *Terminalia catappa* L. against *Gastrothylax crumenifer*. Vet Parasitol. 2021;291:109385. 10.1016/j.vetpar.2021.109385 33667989

[39] Lima CS, Pereira MH, Gainza YA, Hoste H, Regasini LO, Chagas AC de S. Anthelmintic effect of *Pterogyne nitens* (Fabaceae) on eggs and larvae of *Haemonchus contortus*: analyses of structure-activity relationships based on phenolic compounds. Ind crops Prod. 2021;164:113348.

[40] Giovanelli F, Mattellini M, Fichi G, Flamini G, Perrucci S (2018). In vitro anthelmintic activity of four plant-derived compounds against sheep gastrointestinal nematodes. Vet Sci..

[41] Kuntic V, Filipovic I, Vujic Z (2011). Effects of rutin and hesperidin and their Al(III) and cu(II) complexes on in vitro plasma coagulation assays. Molecules..

[42] Gilani R, Mills JL (2022). Vascular complications of surgery and intervention.

[43] Bauça JM, Ajzner É, Cadamuro J, Hillarp A, Kristoffersen AH, Meijer P (2022). An international study on activated partial thromboplastin time prolongation. Part 2: interpretative commenting. Clin Chim Acta..

