# Supplementary material for: Evaluating anthelmintic, anti-platelet, and anti-coagulant activities, and identifying the bioactive phytochemicals of Amaranthus blitum L
Source: BMC Complement Med Ther. 2024 May 4;24:183. doi: 10.1186/s12906-024-04478-2 (PMC11069165; doi:10.1186/s12906-024-04478-2)
Supplement: Supplementary file 1 — Supplementary Material 1. [file 12906_2024_4478_MOESM1_ESM.pdf]

Supplementary material

Evaluating anthelmintic, anti-platelet, and anti-coagulant activities, and identifying the bioactive phytochemicals of *Amaranthus blitum* L.

Ghada A. Abdel-Moez[1]. Hanaa M. Sayed[1]. Azza. A. Khalifa[1]. Salwa M. Abd-Elrahman[2], Mohammed A. Osman[3], Shaymaa M. Mohamed[*,1]

Affiliation

[1]Department of Pharmacognosy, Faculty of Pharmacy, Assiut University, Assiut 71526, Egypt

[2]Department of Parasitology, Faculty of Veterinary Medicine, Assiut University, Assiut 71515, Egypt

[3]Department of Clinical Pathology, Faculty of Medicine, Assiut University, Assiut 71511, Egypt

Correspondence

Dr. Shaymaa Mohamed, Department of Pharmacognosy, Faculty of Pharmacy, Assiut University, Assiut 71526, Egypt

E-mail address: Shaymaa.makram@aun.edu.eg, Tel.: + 01019023522, fax: + 20882080774

List of data

S 1. Isolation of compounds **1–6** from *n*-hexane fraction 1
S 2. Isolation of compounds **7–22** from the polar fraction 1

List of schemes

Scheme S1: Extraction and fractionation of *Amaranthus blitum* L. aerial parts. 3
Scheme S2: Isolation and purification of compounds **1–6** from *n*-hexane fraction of *Amaranthus blitum* L. 4
Scheme S3: Isolation and purification of compounds **7–15** from the polar fraction of *Amaranthus blitum* L. 4

List of figures:

Fig. S1: Positive-ion HR-ESI-MS of compound **19** 5
Fig. S2: Negative-ion HR-ESI-MS of compound **19** 5
Fig. S3: ^{1}H NMR spectrum of compound **19** (CD_3OD, 400 MHz) 6
Fig. S4: APT spectrum of compound **19** (CD_3OD, 100 MHz) 6
Fig. S5: HSQC spectrum of compound **19** (CD_3OD, 400/100 MHz) 7
Fig. S6: HMBC spectrum of compound **19** (CD_3OD, 400/100 MHz) 7
Fig. S7: LC-MS/ESI spectrum of compound **1** 8
Fig. S8: ^{1}H NMR spectrum of compound **1** ($CDCl_3$, 400 MHz) 8
Fig. S9: ^{13}C (APT) NMR spectrum of compound **1** ($CDCl_3$, 100 MHz) 9
Fig. S10: ^{1}H NMR spectrum of compound **2** (C_5D_5N, 400 MHz) 9
Fig. S11: ^{13}C (APT) NMR spectrum of compound **2** (C_5D_5N, 100 MHz) 10
Fig. S12: ^{1}H NMR spectrum of compound **3** ($CDCl_3$, 400 MHz) 10
Fig. S13: ^{13}C (APT) NMR spectrum of compound **3** ($CDCl_3$, 100 MHz) 11
Fig. S14: Positive HR-ESI-MS of compound **4** 11
Fig. S15: Negative HR-ESI-MS of compound **4** 12
Fig. S16: ^{1}H NMR spectrum of compound **4** (C_5D_5N, 400 MHz) 12
Fig. S17: ^{13}C (APT) NMR spectrum of compound **4** (C_5D_5N, 100 MHz) 13
Fig. S18: HSQC spectrum of compound **4** (C_5D_5N, 400/100 MHz) 13
Fig. S19: HMBC spectrum of compound **4** (C_5D_5N, 400/100 MHz) 14

Fig. S20: Positive HR-ESI-MS of compound **5** 14
Fig. S21: ^{1}H NMR spectrum of compound **5** ($CDCl_3$, 400 MHz) 15
Fig. S22: ^{13}C (APT) NMR spectrum of compound **5** ($CDCl_3$, 100 MHz) 15
Fig. S23: Positive HR-ESI-MS of compound **6** 16
Fig. S24: Negative HR-ESI-MS of compound **6** 16
Fig. S25: ^{1}H NMR spectrum of compound **6** ($CDCl_3$, 400 MHz) 17
Fig. S26: ^{13}C (APT) NMR spectrum of compound **6** ($CDCl_3$, 100 MHz) 17
Fig. S27: ^{1}H NMR spectrum of compound **7** (DMSO-d_6, 400 MHz) 18
Fig. S28: ^{13}C (APT) NMR spectrum of compound **7** (DMSO-d_6, 400 MHz) 18
Fig. S29: Positive HR-ESI-MS of compound **8** 19
Fig. S30: Negative HR-ESI-MS of compound **8** 19
Fig. S31: ^{1}H NMR spectrum of compound **8** (DMSO-d_6, 400 MHz) 20
Fig. S32: ^{13}C (APT) NMR spectrum of compound **8** (DMSO-d_6, 100 MHz) 20
Fig. S33: ^{1}H-^{1}H COSY spectrum of compound **8** (DMSO-d_6, 400 MHz) 21
Fig. S34: HMBC spectrum of compound **8** (DMSO-d_6, 400/100 MHz) 21
Fig. S35: ^{1}H NMR spectrum of compound **9** (DMSO-d_6, 400 MHz) 22
Fig. S36: ^{13}C (APT) NMR spectrum of compound **9** (DMSO-d_6, 100 MHz) 22
Fig. S37 : ^{1}H NMR spectrum of compound **10** (DMSO-d_6, 400 MHz) 23
Fig. S38: ^{13}C (APT) NMR spectrum of compound **10** (DMSO-d_6, 100 MHz) 23
Fig. S39: ^{1}H NMR spectrum of compound **11** (DMSO-d_6, 400 MHz) 24
Fig. S40: ^{13}C (APT) NMR spectrum of compound **11** (DMSO-d_6, 100 MHz) 24
Fig. S41: Positive HR-ESI-MS of compound **12** 25
Fig. S42: Negative HR-ESI-MS of compound **12** 25
Fig. S43: ^{1}H NMR spectrum of compound **12** (C_5D_5N, 400 MHz) 26
Fig. S44: ^{13}C (APT) NMR spectrum of compound **12** (C_5D_5N, 400 MHz) 26
Fig. S45: HSQC spectrum of compound **12** (C_5D_5N, 400/100 MHz) 27
Fig. S46: HMBC spectrum of compound **12** (C_5D_5N, 400/100 MHz) 27
Fig. S47: ^{1}H NMR spectrum of compound **13** (DMSO-d_6, 400 MHz) 28
Fig. S48: ^{13}C (APT) NMR spectrum of compound **13** (DMSO-d_6, 100 MHz) 28
Fig. S49: ^{1}H NMR spectrum of compound **14** (DMSO-d_6, 400 MHz) 29
Fig. S50: ^{13}C (APT) NMR spectrum of compound **14** (DMSO-d_6, 100 MHz) 29

Fig. S51: HSQC spectrum of compound **14** (DMSO-d_6, 400/100 MHz) 30
Fig. S52: HMBC spectrum of compound **14** (DMSO-d_6, 400/100 MHz) 30
Fig. S53: ^{1}H NMR spectrum of compound **15** (DMSO-d_6, 400 MHz) 31
Fig. S54: ^{13}C (APT) NMR spectrum of compound **15** (DMSO-d_6, 100 MHz) 31
Fig. S55: ^{1}H NMR spectrum of compound **16** (DMSO-d_6, 400 MHz) 32
Fig. S56: ^{13}C (APT) NMR spectrum of compound **16** (DMSO-d_6, 100 MHz) 32
Fig. S57: HSQC spectrum of compound **16** (DMSO-d_6, 400/100 MHz) 33
Fig. S58: HMBC spectrum of compound **16** (DMSO-d_6, 400/100 MHz) 33
Fig. S59: ^{1}H NMR spectrum of compound **17** (DMSO-d_6, 400 MHz) 34
Fig. S60: ^{13}C (APT) NMR spectrum of compound **17** (DMSO-d_6, 100 MHz) 34
Fig. S61: HSQC spectrum of compound **17** (DMSO-d_6, 400/100 MHz) 35
Fig. S62: ^{1}H NMR spectrum of compound **18** (DMSO-d_6, 400 MHz) 35
Fig. S63: ^{13}C (APT) NMR spectrum of compound **18** (DMSO-d_6, 100 MHz) 36
Fig. S64: HSQC spectrum of compound **18** (DMSO-d_6, 400/100 MHz) 36
Fig. S65: HMBC spectrum of compound **18** (DMSO-d_6, 400/100 MHz) 37
Fig. S66: ^{1}H NMR spectrum of compound **20** (CD_3OD, 400 MHz) 37
Fig. S67: ^{13}C (APT) NMR spectrum of compound **20** (CD_3OD, 100 MHz) 38
Fig. S68: HSQC spectrum of compound **20** (CD_3OD, 400/100 MHz) 38
Fig. S69: HMBC spectrum of compound **20** (CD_3OD, 400/100 MHz) 39
Fig. S70: ^{1}H NMR spectrum of compound **21** (DMSO-d_6, 400 MHz) 39
Fig. S71: ^{13}C (APT) NMR spectrum of compound **21** (DMSO-d_6, 100 MHz) 40
Fig. S72: ^{1}H NMR spectrum of compound **22** (DMSO-d_6, 400 MHz) 40
Fig. S73: ^{13}C (APT) NMR spectrum of compound **22** (DMSO-d_6, 400 MHz) 41
Fig. S74: Microscopic examination of active motile *Trichinella spiralis* larvae, scale bars are 100 µm. 42
Fig. S75: Microscopic examination of weak coiled *Trichinella spiralis* larvae (wide circle shaped), scale bars are 100 µm. 42
Fig. S76: Microscopic examination of completely dead *Trichinella spiralis* larvae (comma shaped), scale bars are 100 µm. 42
Fig. S77: Microscopic examination of C-shaped *Trichinella spiralis* larvae, scale bars are 100 µm. 43

Fig. S78: Microscopic examination of typical supercoiled *Trichinella spiralis* larvae, scale bars are 100 µm. 43
Fig. S79: Scanning electron microscopy of crude ethanolic extract-treated groups, showing opacity and loss of normal striation, pores and holes (red arrow), and blebs (green arrows) 44
Fig. S80: Scanning electron microscopy of *n*-hexane-treated larvae, showing pores and holes (red arrows), and blebs (green arrow) 44
Fig. S81: Scanning electron microscopy of polar fraction-treated larvae, showing opacity shortening, pores and holes (red arrow), sloughing of some areas and detachment of the cuticles (green arrow). 44

S 1. Isolation of compounds **1**–**6** from *n*-hexane fraction

F2 (30 g) was subjected to silica gel (900 g) CC, eluted with (*n*-Hex-Ace, from 99:1 to 90:10), to afford four subfractions (F2-A–F2-D). F2-B (11 g) was subjected to silica gel CC (300 g), eluted with (*n*-Hex-EtOAc, from 98:2 to 95:5), to give compound **1** (57 mg). F2-D (15 g) was applied over silica gel CC (400 g), eluted with (*n*-Hex-Ace, from 99:1 to 97:3) to give three subfraction, F2-D-1– F2-D-3. F2-D-2 (1.5 g) was re-chromatographed on florisil CC (100 g), eluted with (*n*-Hex-Ace, from 99:1 to 97.5:2.5), to afford compound **2** (216 mg) and a mixture of compounds **2** and **3** (92 mg). This mixture was applied over florisil CC (100 g), eluted with (*n*-Hex-EtOAc, from 99:1 to 97:3), to separate compound **3** (10 mg) and an additional amount of compound **2** (24 mg). F3 (25 g) was re-chromatographed on silica gel CC (700 g), eluted with (DCM-Ace, from 20:1 to 8:2), to afford four subfractions F3-A–F3-D. F3-A (10 g) was fractionated using silica gel CC (300 g), eluted with (*n*-Hex-Ace, from 90:10 to 85:15), followed by alumina CC (50 g), eluted with (*n*-Hex-Ace, 8.5:1.5), then purified using florisil CC (100 g), eluted with (*n*-Hex-Ace, from 9:1 to 6:4), to yield compound **4** (55 mg). F3-C (8 g) was fractionated over silica gel CC (250 g), eluted with *n*-Hex, then (*n*-Hex-Ace, from 95:5 to 85:15), to afford a subfraction F3-C-1 (871 mg), among others. F3-C-1 was subjected to florisil CC (100 g), eluted with (DCM-EtOAc, 9:1), to afford an additional amount of compound **4** (9 mg) and a subfraction F3-C-1a. Purification of F3-C-1a using Sephadex LH-20 (100 g), eluted with (DCM-MeOH, 1:1), followed by silica gel CC (150 g), eluted with *n*-Hex-Ace, from 9:1 to 8:2), yielded compound **5** (10 mg). **F4** (8 g) was subdivided into two subfractions F4-A and F4-B after silica gel CC (250 g), eluted with (DCM-Ace,10:2). F4-B (4 g) when chromatographed over florisil CC (100 g), eluted with (*n*-Hex-Ace, 9:1), provided F4-B-1 (65 mg). Purification of F4-B-1 over silica gel SPE column, eluted with (*n*-Hex-Ace, 95:5), gave a subfraction F4-B1a (22 mg) that was subjected to Sephadex LH-20 CC (100 g) using (DCM-MeOH, 1:1), followed by a silica gel SPE CC, eluted with (*n*-Hex-Ace, 85:15), to afford compound **6** (12 mg).

S 2. Isolation of compounds **7**–**22** from the polar fraction

F-I (50 mg) was chromatographed over Sephadex LH-20 (500 g), eluted with MeOH, to afford two subfractions (F-I-1 and F-I-2). F-I-1 (160 mg) was purified by repeated silica gel SPE CC, eluted with (DCM-MeOH, 25:1) to give compound **7** (10 mg). F-I-2 (30 mg) was subjected to a silica gel SPE CC, eluted with (DCM-MeOH, 15:1) to afford a mixture of compounds **8** and **9**.

This mixture was separated using reversed phase silica gel (C-18) RP SPE, eluted with 100% H_2O to give compound **8** (3 mg) and then 100% MeOH to yield compound **9** (3 mg). F-II (110 mg) was chromatographed over Sephadex LH-20 (500 g), eluted with MeOH, to afford F-II-1 (67 mg) and compound **11** (44 mg). Purification of F-II-1 using silica gel SPE CC, eluted with (EtOAc-DCM-MeOH, 8:5:3), gave compound **10** (20 mg). F-VI (404 mg) was chromatographed over Sephadex LH-20 (500 g), eluted with MeOH, to give three groups (F-VI-1–F-VI-3). F-VI-1 (84 mg) was subjected to reversed phase silica gel (C-18) RP SPE CC, eluted with (H_2O-MeOH, 1:1) to yield compound **12** (11 mg). F-VI-2 (38 mg) was adsorbed over silica gel SPE, eluted with (DCM-MeOH, 17:1) to afford compound **13** (10 mg) and then compound **14** (15 mg) was separated with (EtOAc-DCM-MeOH-H_2O, 8:5:3:1). F-VI-3 (50 mg) was purified using Sephadex LH-20 followed by a silica gel SPE CC, eluted with (DCM-MeOH, 17:1) to afford compound **15** (6 mg). F-VII (149 mg) was subjected to repeated Sephadex LH-20, eluted with MeOH, to afford compound **16** (29 mg). F-VIII (529 mg) was chromatographed over Sephadex LH-20 (500 g), eluted with MeOH, to afford a subfraction F-VIII-1 and compound **18** (47 mg). F-VIII-1 (159 mg) was chromatographed over Sephadex LH-20 (500 g), eluted with MeOH, to give compound **17** (13 mg) and an additional amount of compound **18** (38 mg). F-X (344 mg) was chromatographed over Sephadex LH-20 (500 g), eluted with MeOH, to afford compound **19** (52 mg) and compound **21** (18 mg). F-XI (160 mg) was purified over Sephadex LH-20 (500 g), eluted with MeOH, to yield compound **20** (27 mg). F-XII (265 mg) was chromatographed over Sephadex LH-20 (500 g), eluted with MeOH, to afford compound **22** (31 mg).

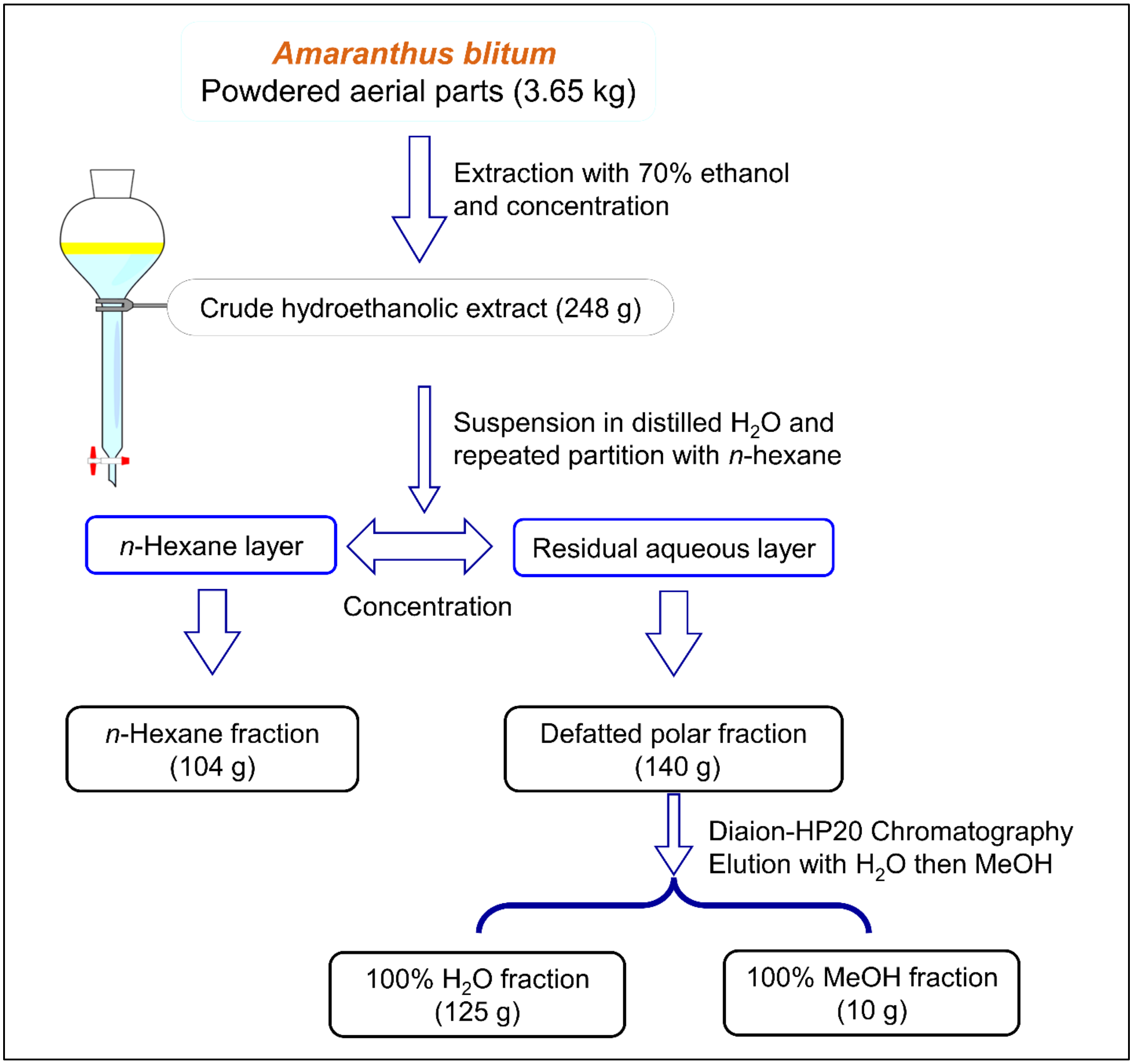

Scheme S1: Extraction and fractionation of *Amaranthus blitum* L. aerial parts.

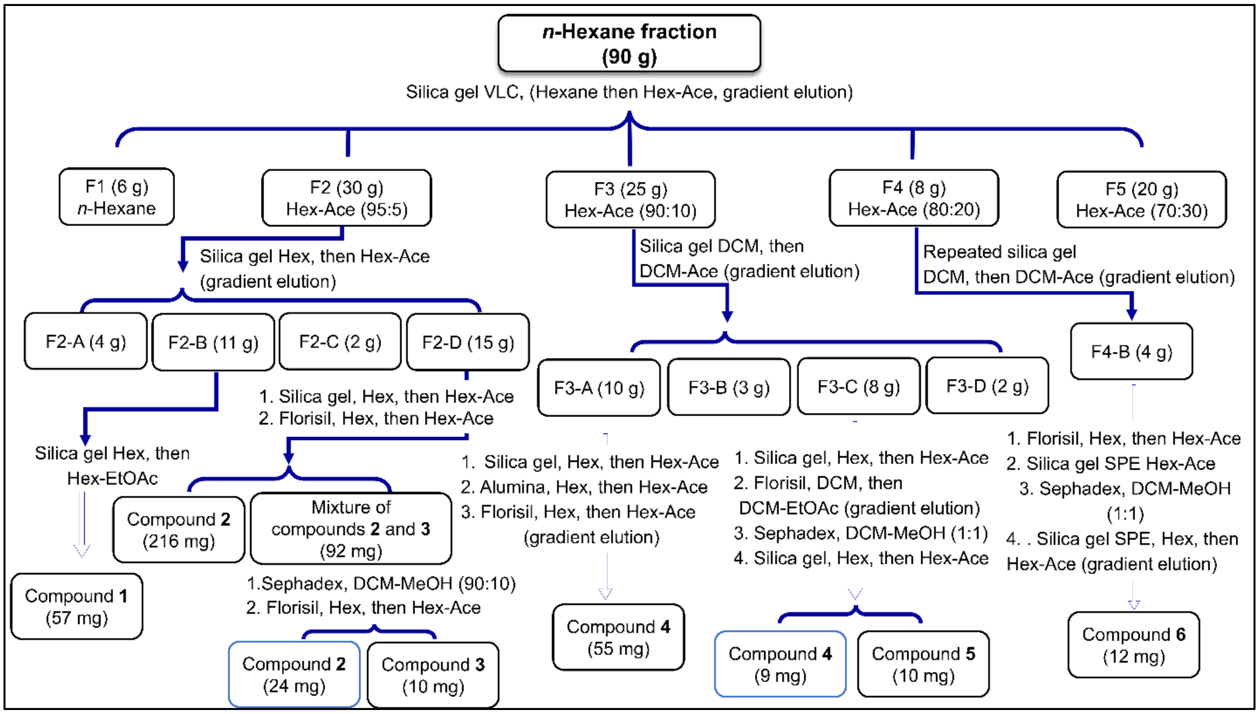

Scheme S2: Isolation and purification of compounds **1**–**6** from *n*-hexane fraction of *Amaranthus blitum* L.

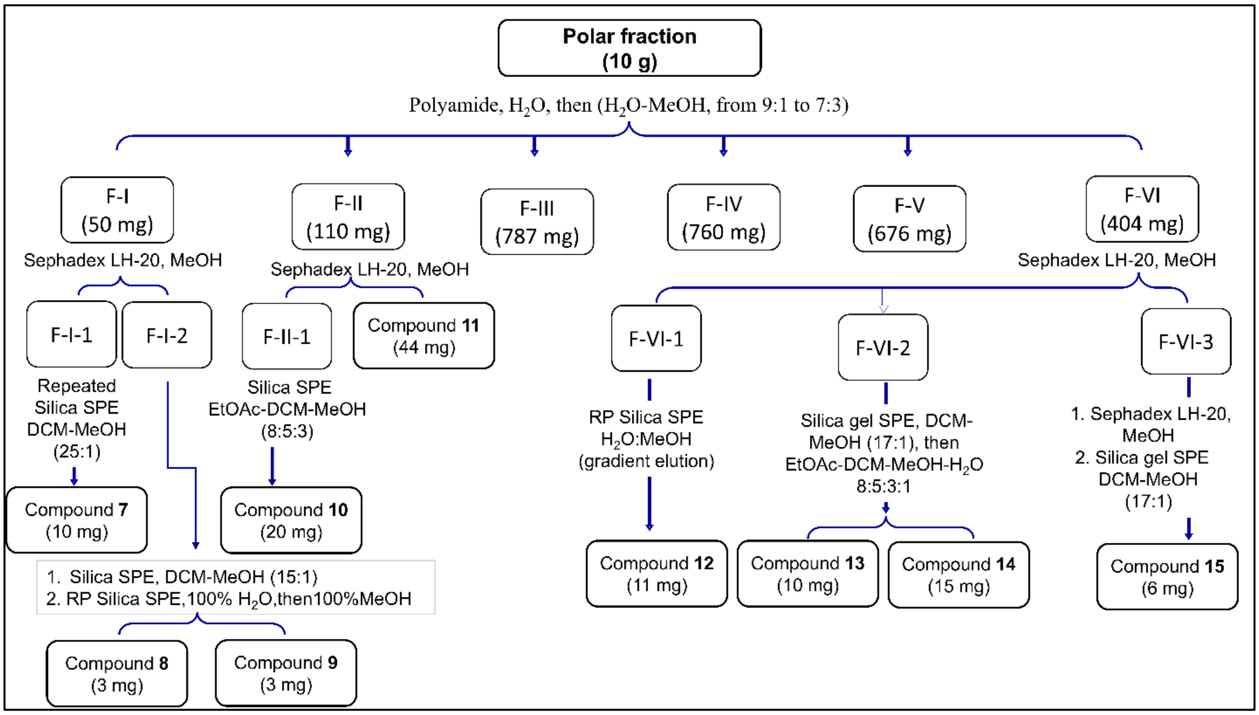

Scheme S3: Isolation and purification of compounds **7**–**15** from the polar fraction of *Amaranthus blitum* L.

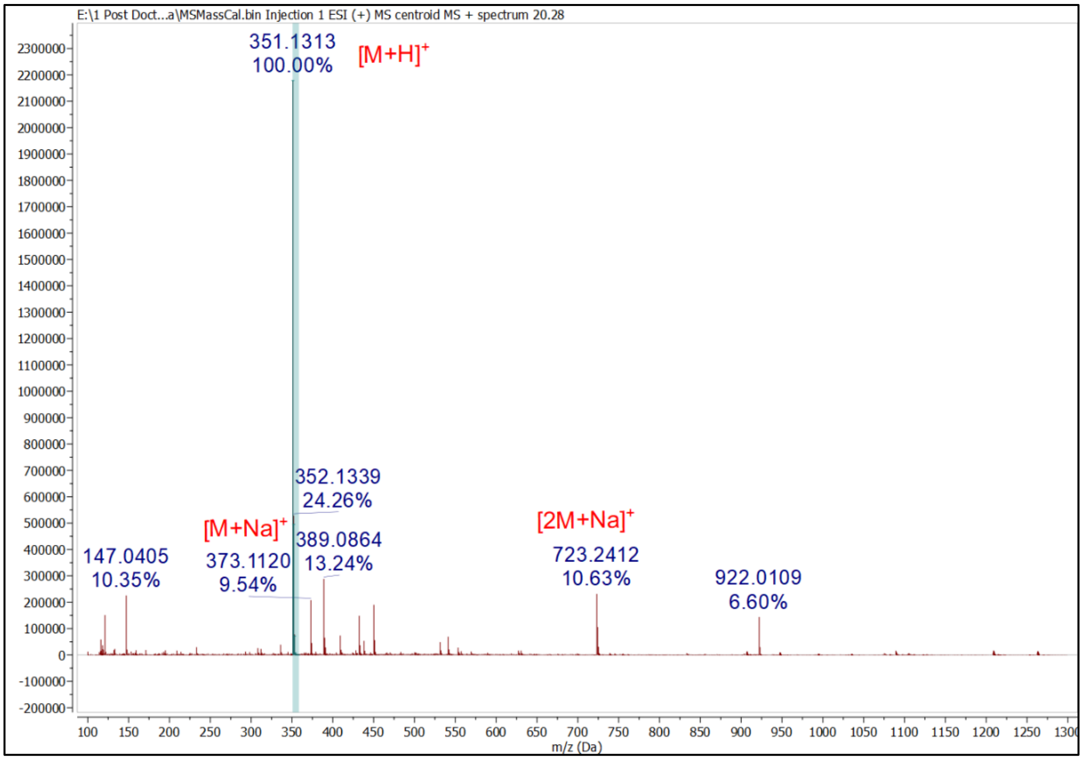

Fig. S1: Positive-ion HR-ESI-MS of compound **19**

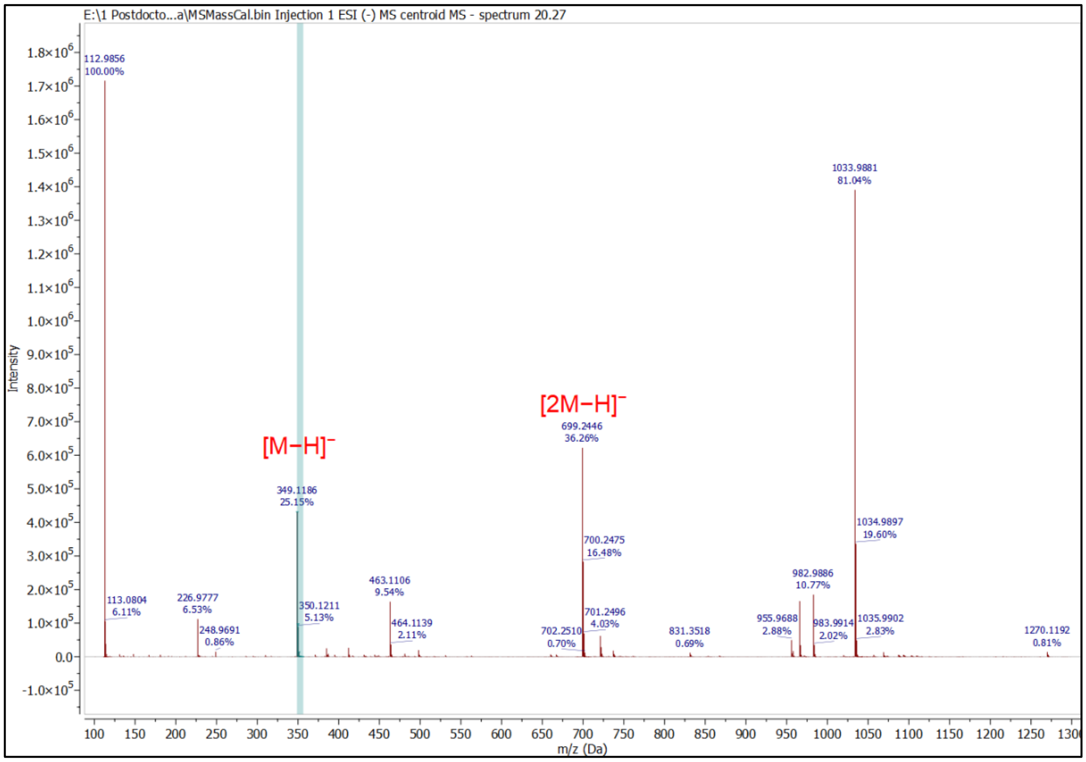

Fig. S2: Negative-ion HR-ESI-MS of compound **19**

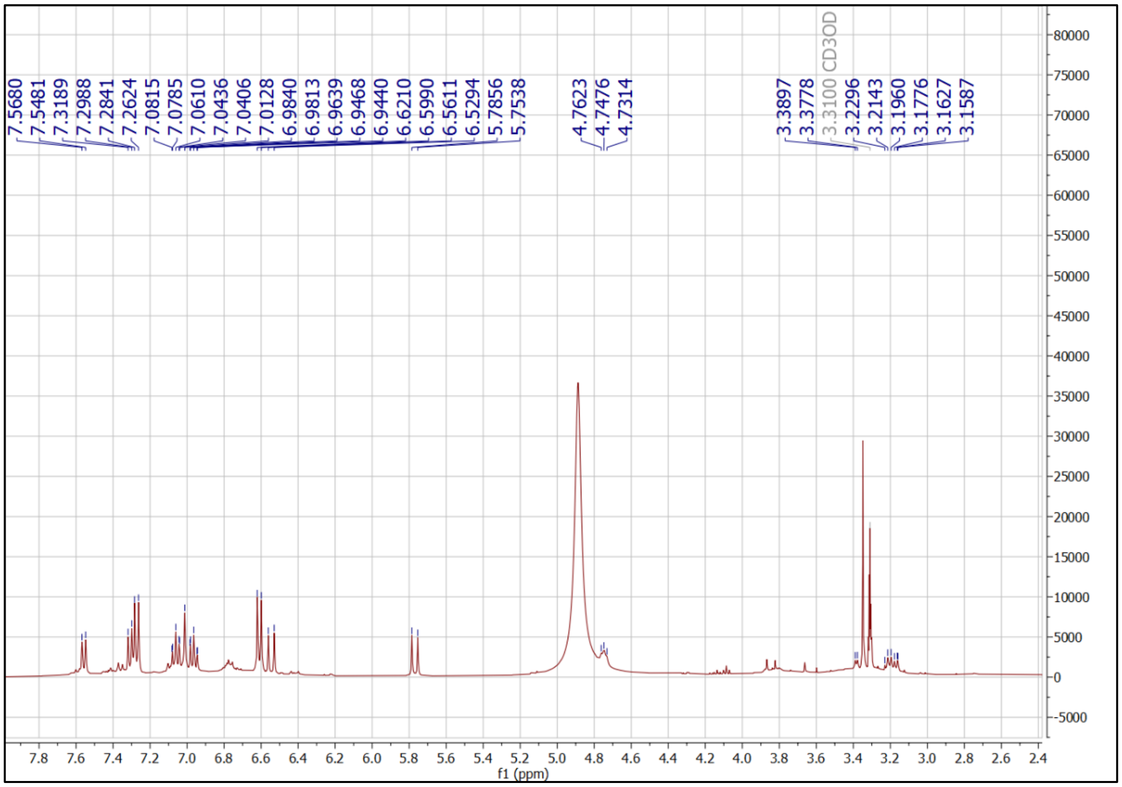

Fig. S3: ^{1}H NMR spectrum of compound **19** (CD_3OD, 400 MHz)

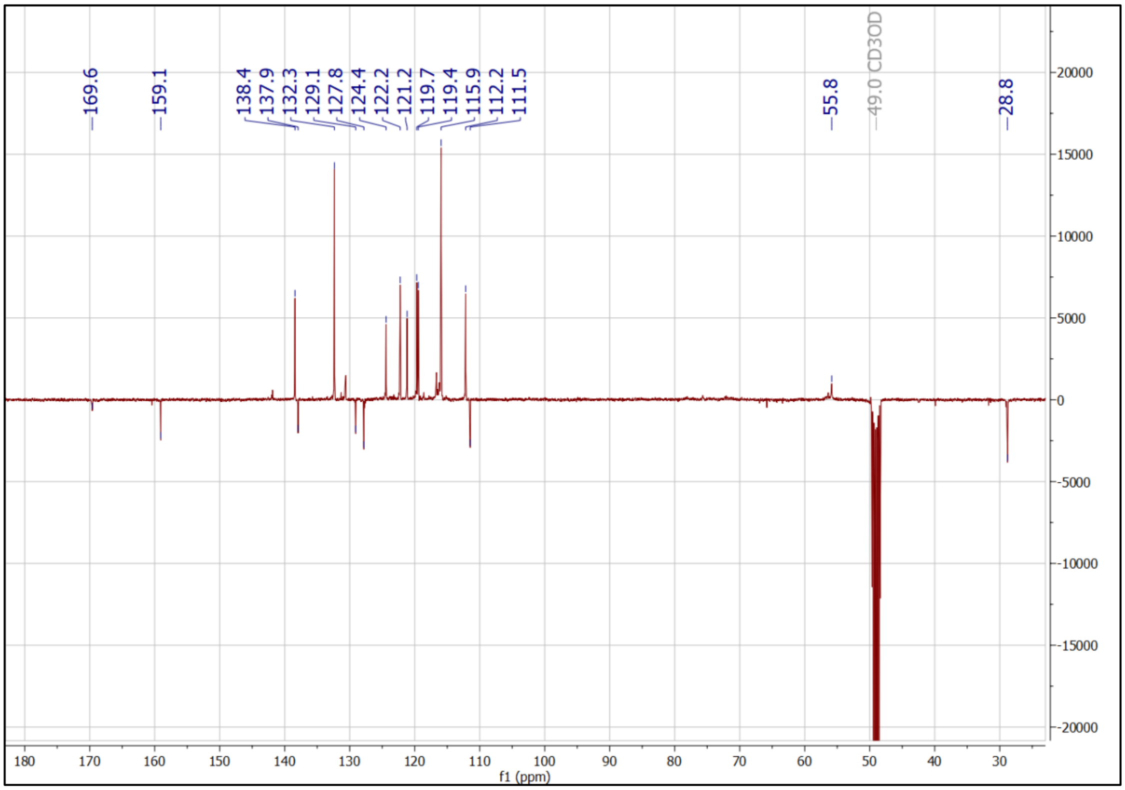

Fig. S4: APT spectrum of compound **19** (CD_3OD, 100 MHz)

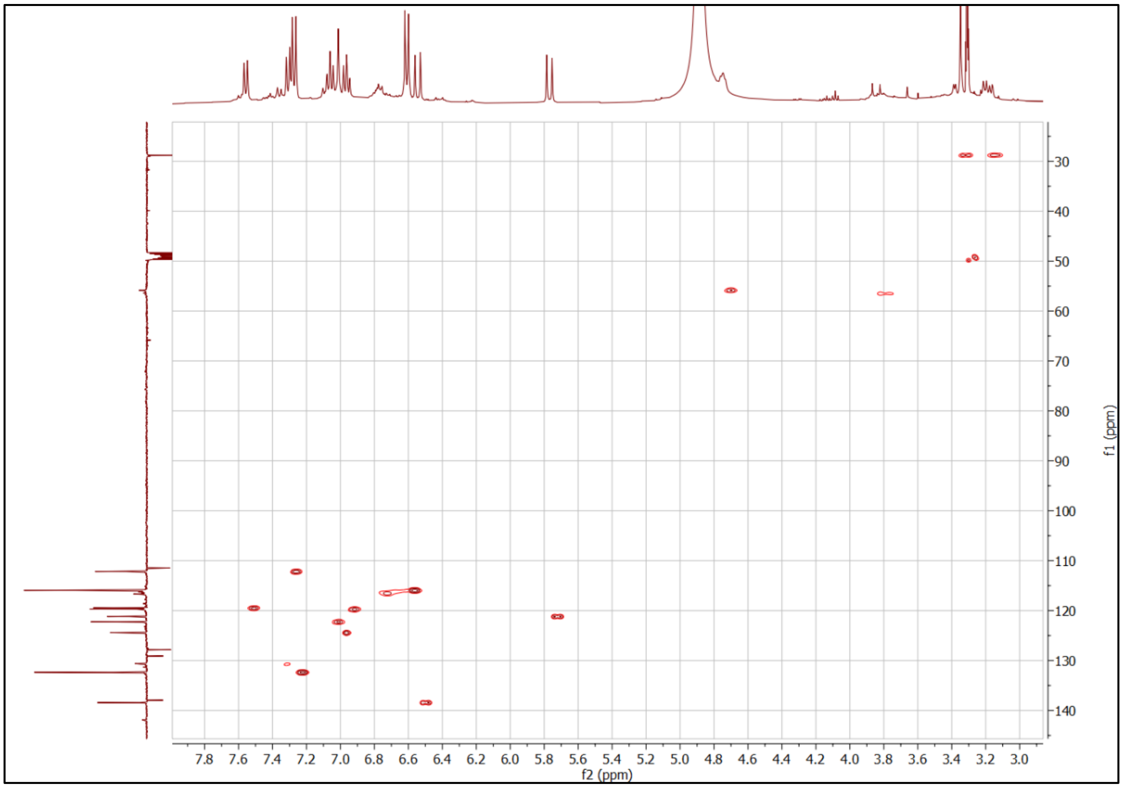

Fig. S5: HSQC spectrum of compound **19** (CD_3OD, 400/100 MHz)

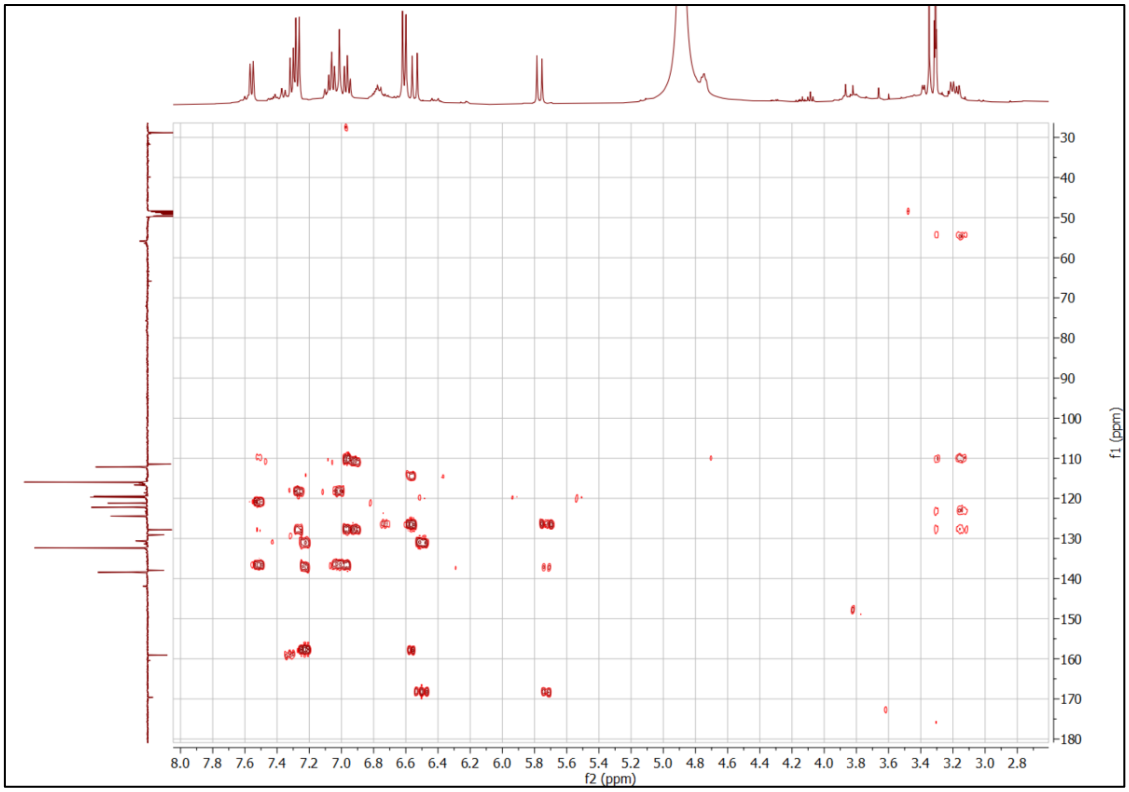

Fig. S6: HMBC spectrum of compound **19** (CD_3OD, 400/100 MHz)

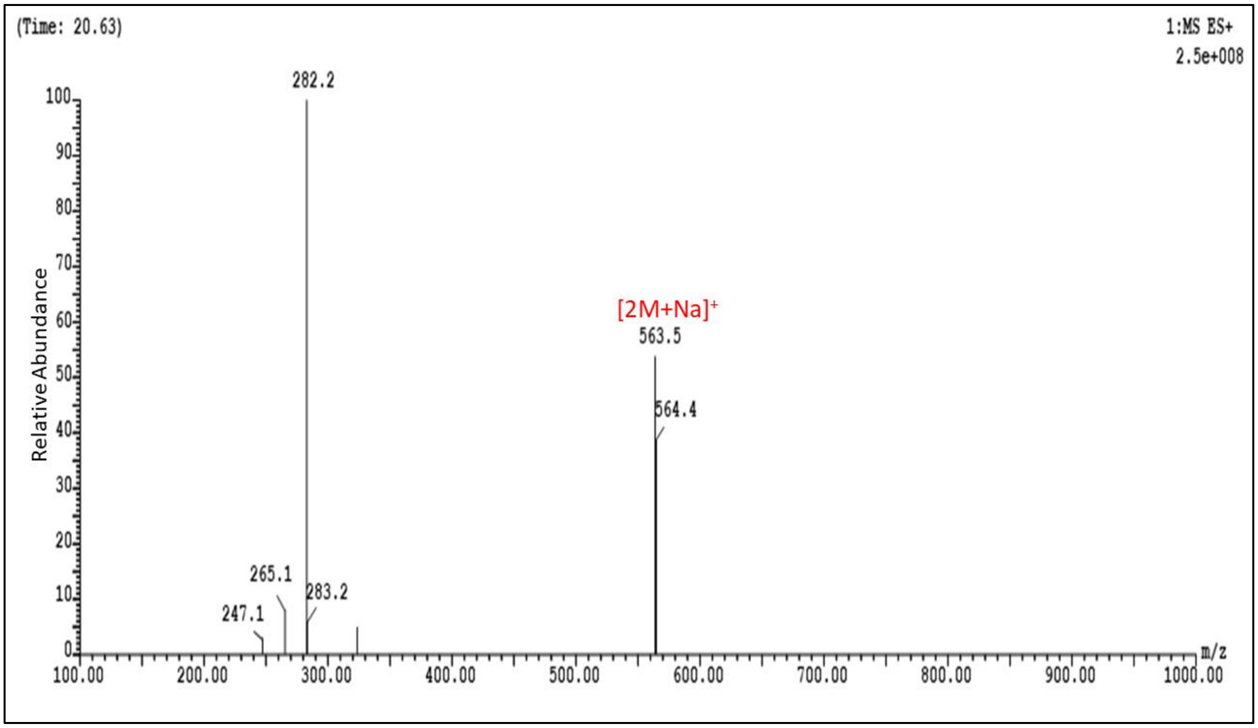

Fig. S7: LC-MS/ESI spectrum of compound **1**

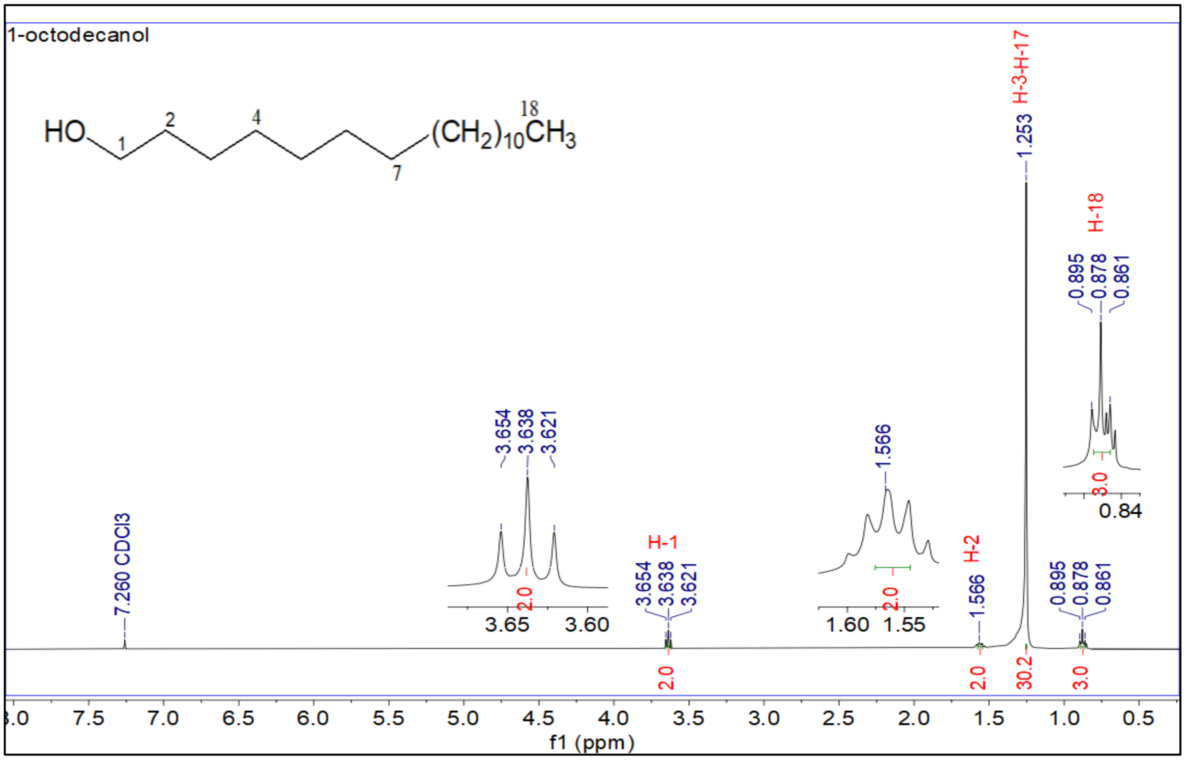

Fig. S8: ^{1}H NMR spectrum of compound **1** ($CDCl_3$, 400 MHz)

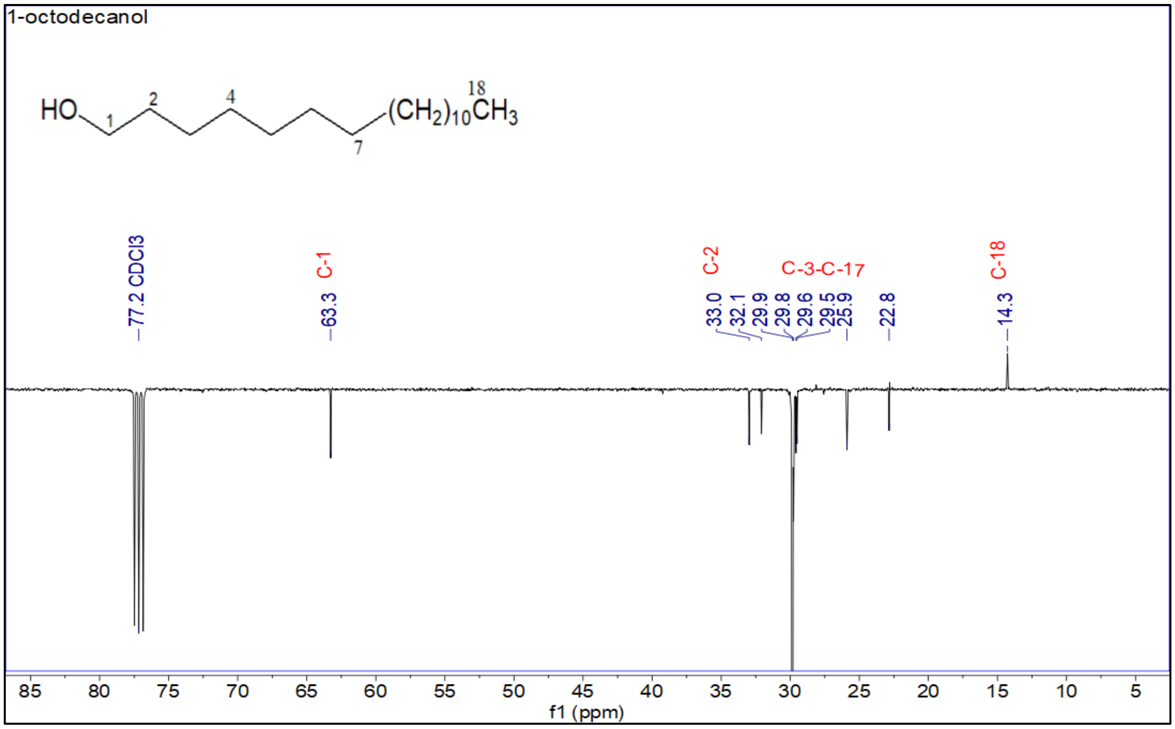

Fig. S9: ^{13}C (APT) NMR spectrum of compound **1** ($CDCl_3$, 100 MHz)

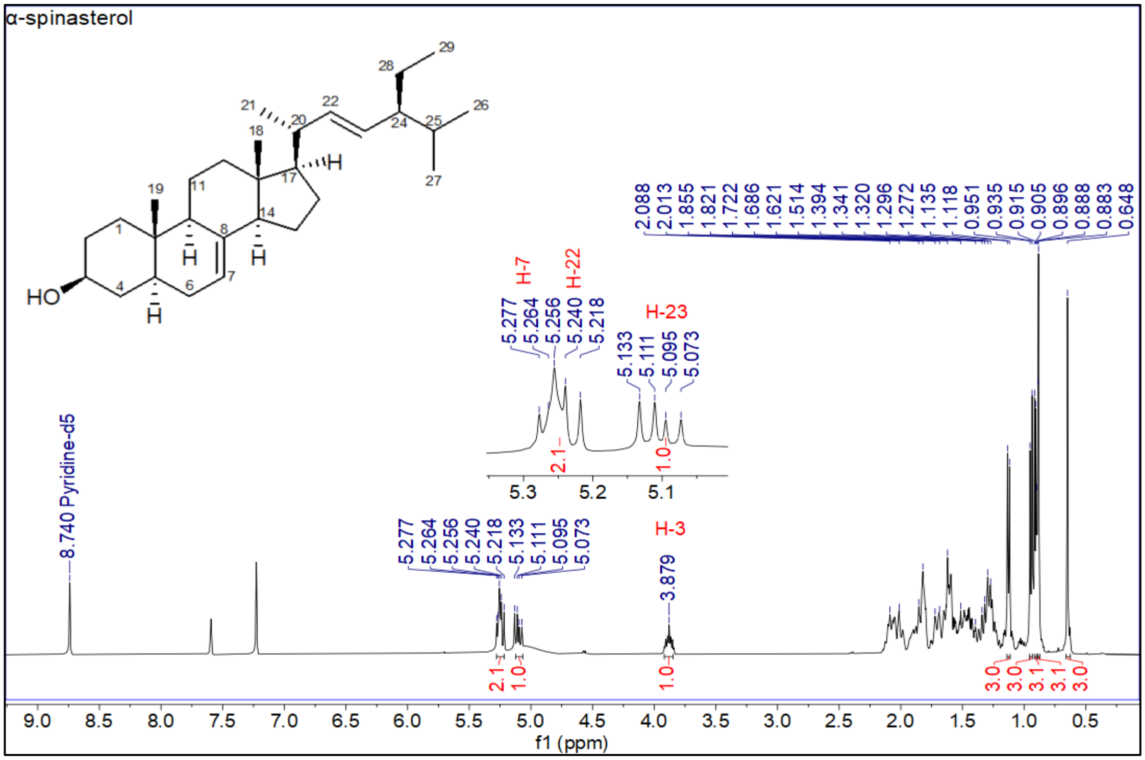

Fig. S10: ^{1}H NMR spectrum of compound **2** (C_5D_5N, 400 MHz)

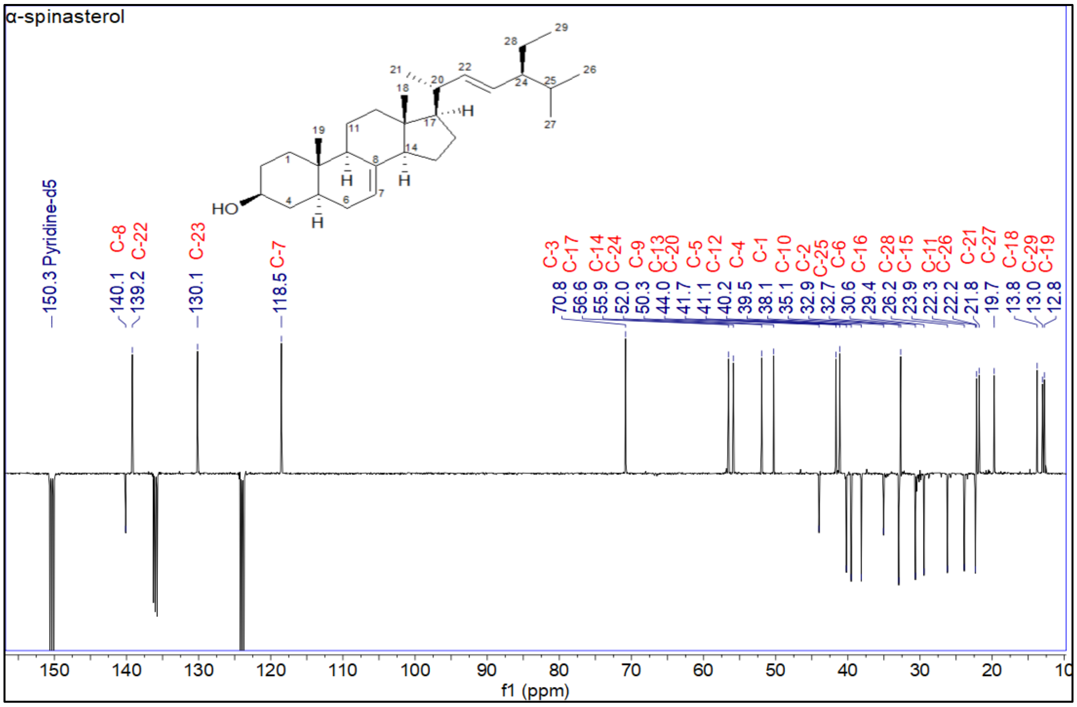

Fig. S11: ^{13}C (APT) NMR spectrum of compound **2** (C_5D_5N, 100 MHz)

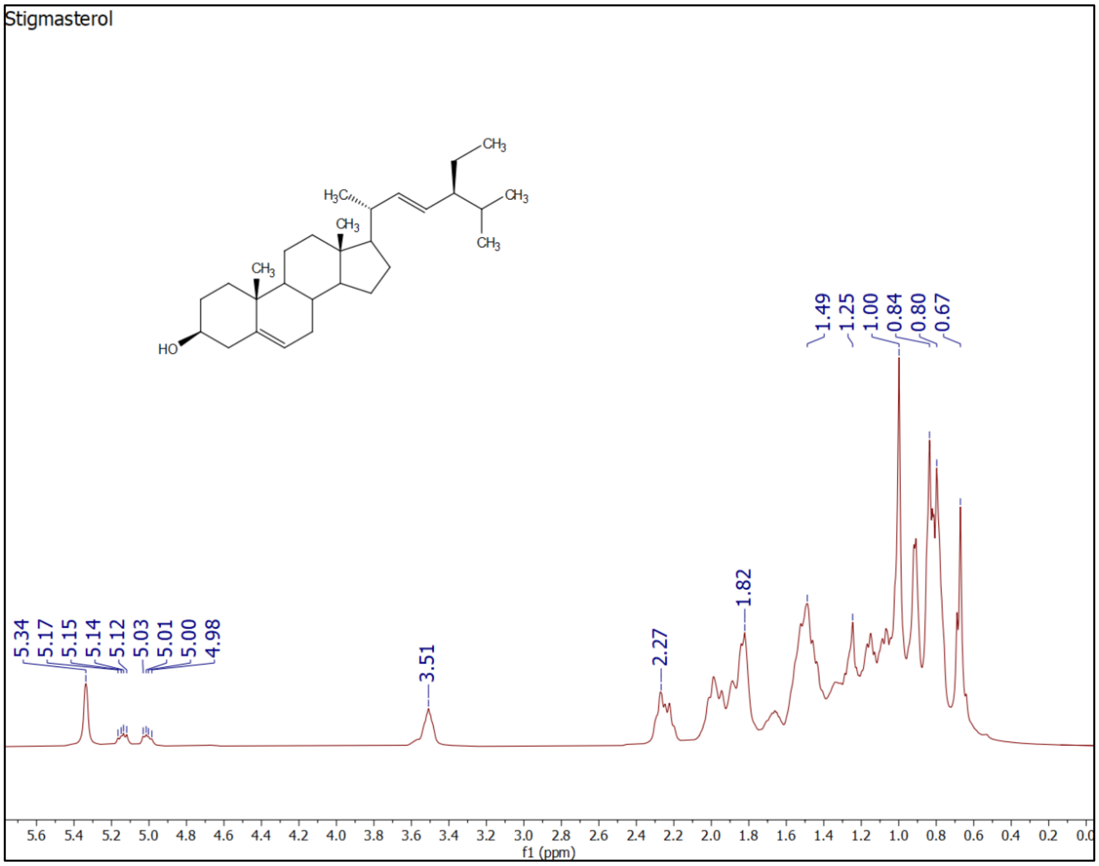

Fig. S12: ^{1}H NMR spectrum of compound **3** ($CDCl_3$, 400 MHz)

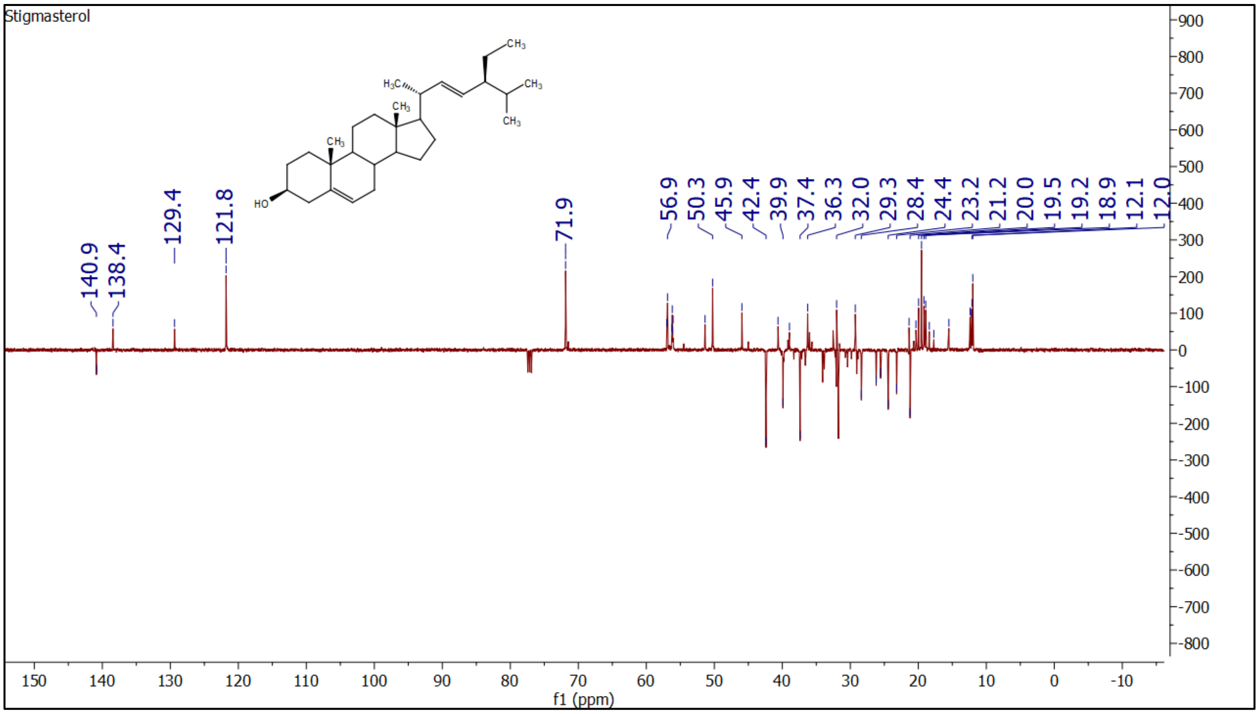

Fig. S13: ^{13}C (APT) NMR spectrum of compound **3** ($CDCl_3$, 100 MHz)

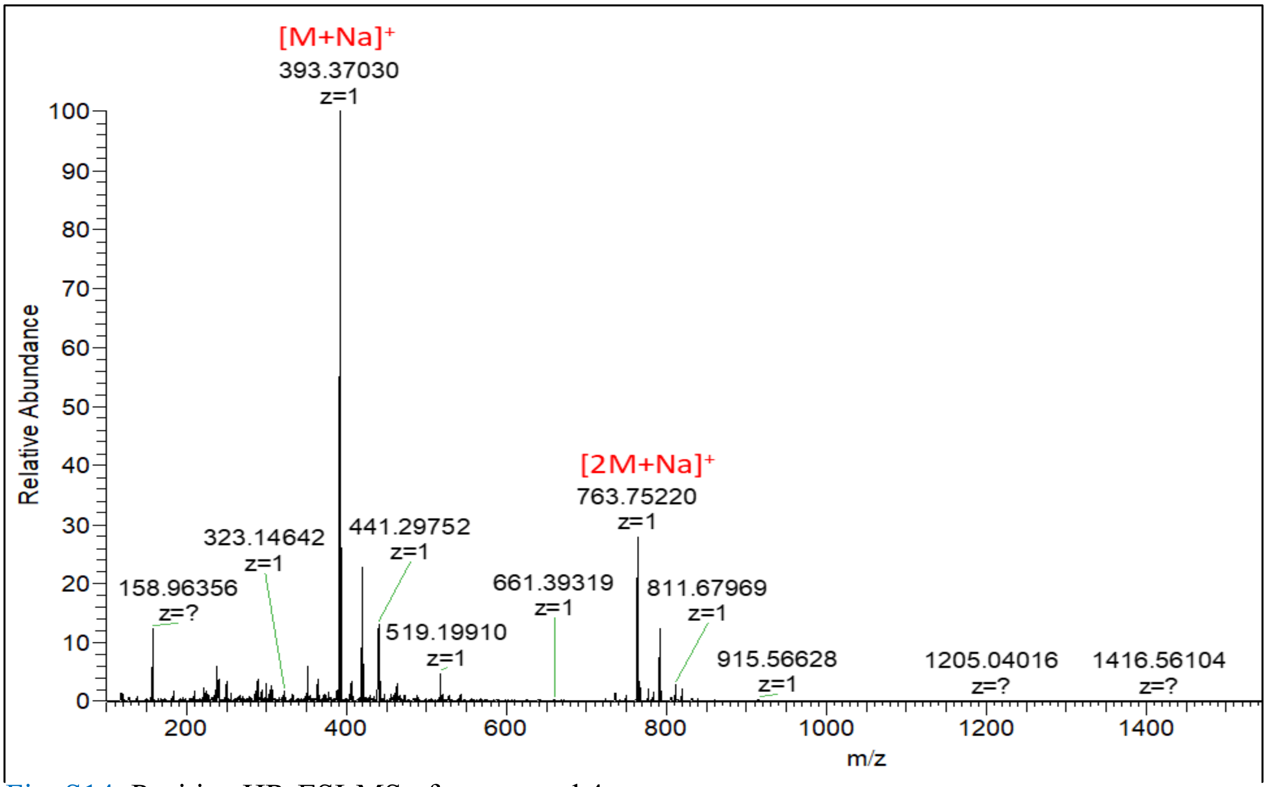

Fig. S14: Positive HR-ESI-MS of compound 4

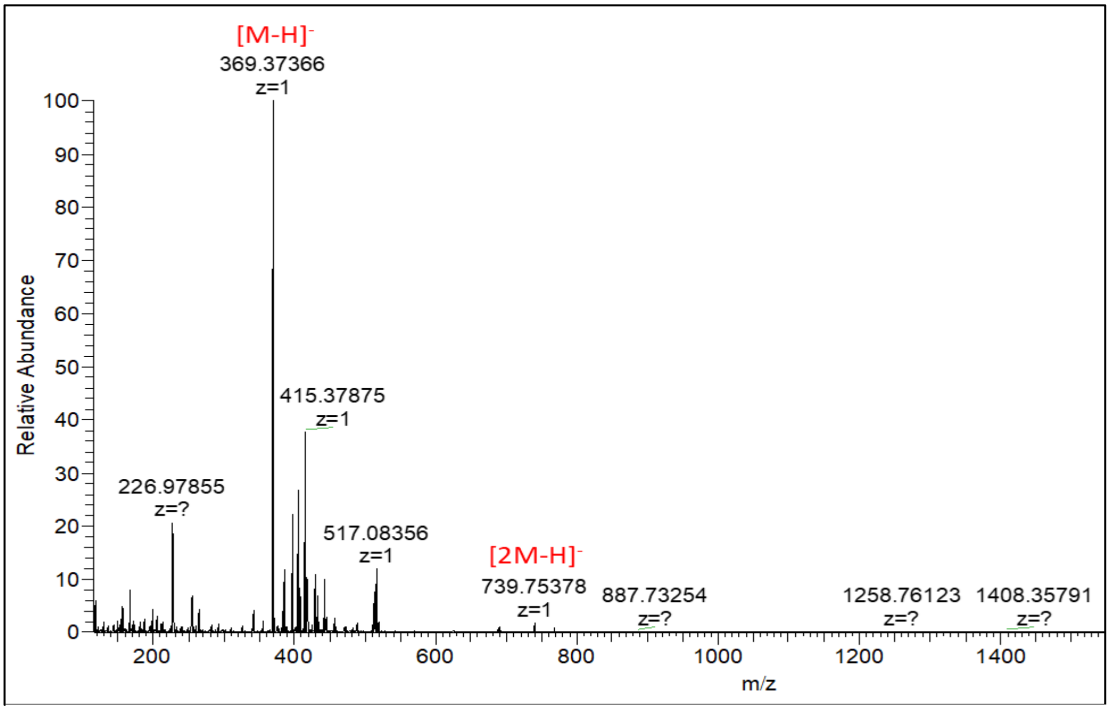

Fig. S15: Negative HR-ESI-MS of compound **4**

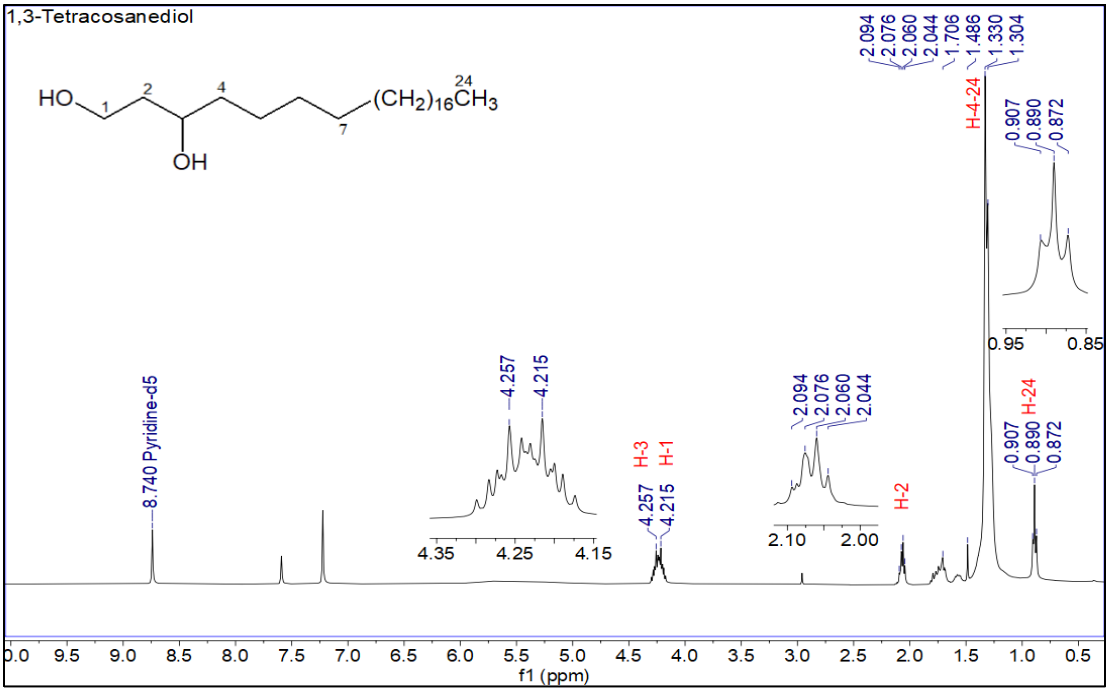

Fig. S16: ^{1}H NMR spectrum of compound **4** (C_5D_5N, 400 MHz)

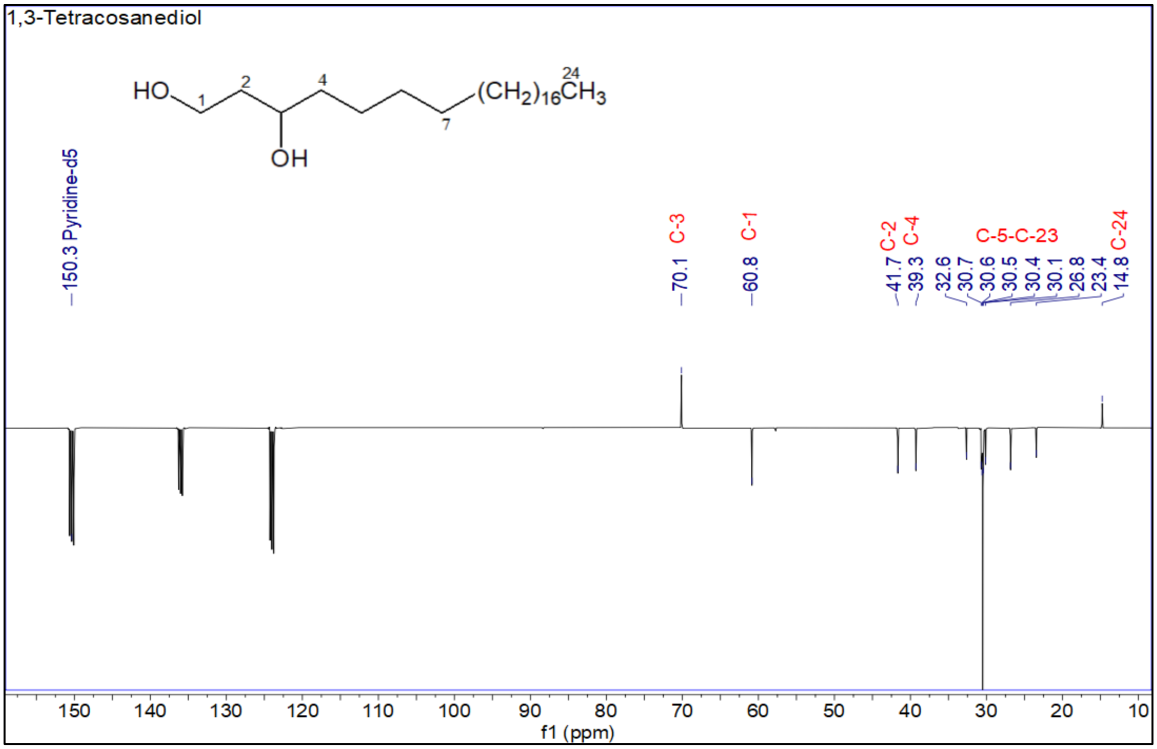

Fig. S17: ^{13}C (APT) NMR spectrum of compound **4** (C_5D_5N, 100 MHz)

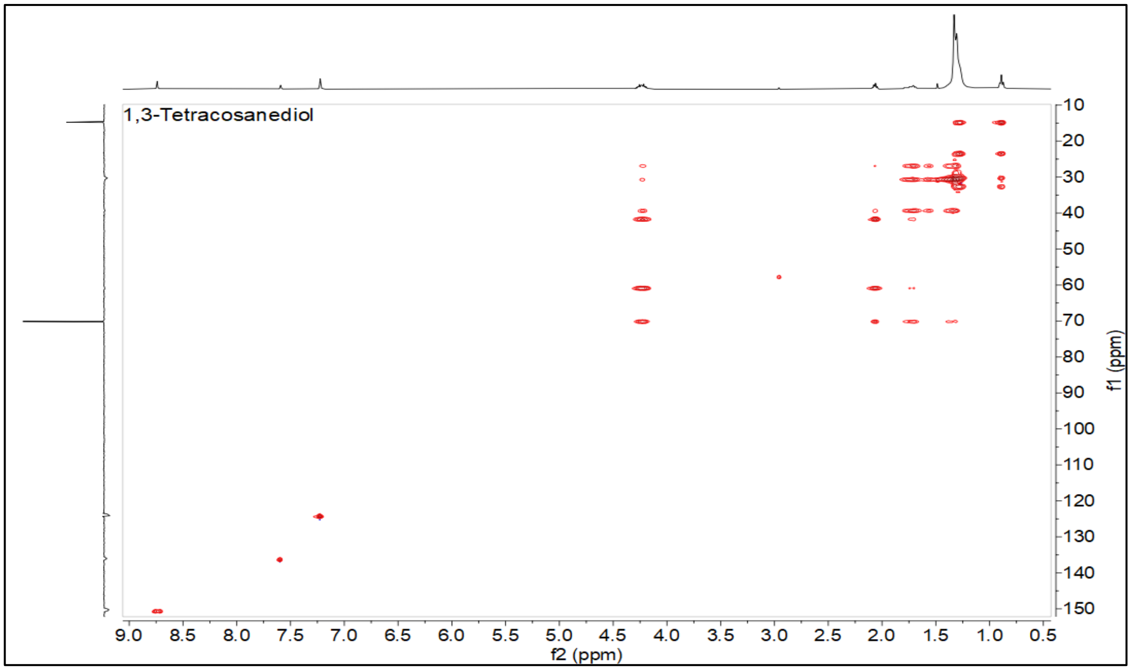

Fig. S18: HSQC spectrum of compound 4 (C_5D_5N, 400/100 MHz)

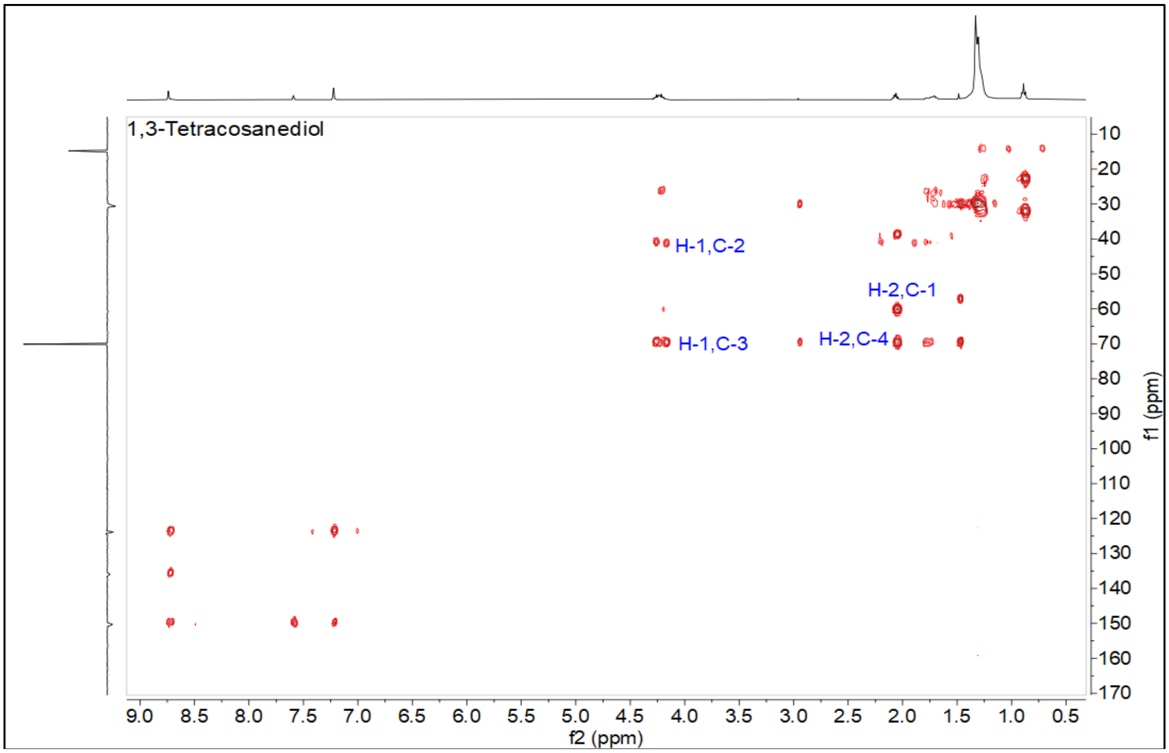

Fig. S19: HMBC spectrum of compound **4** (C_5D_5N, 400/100 MHz)

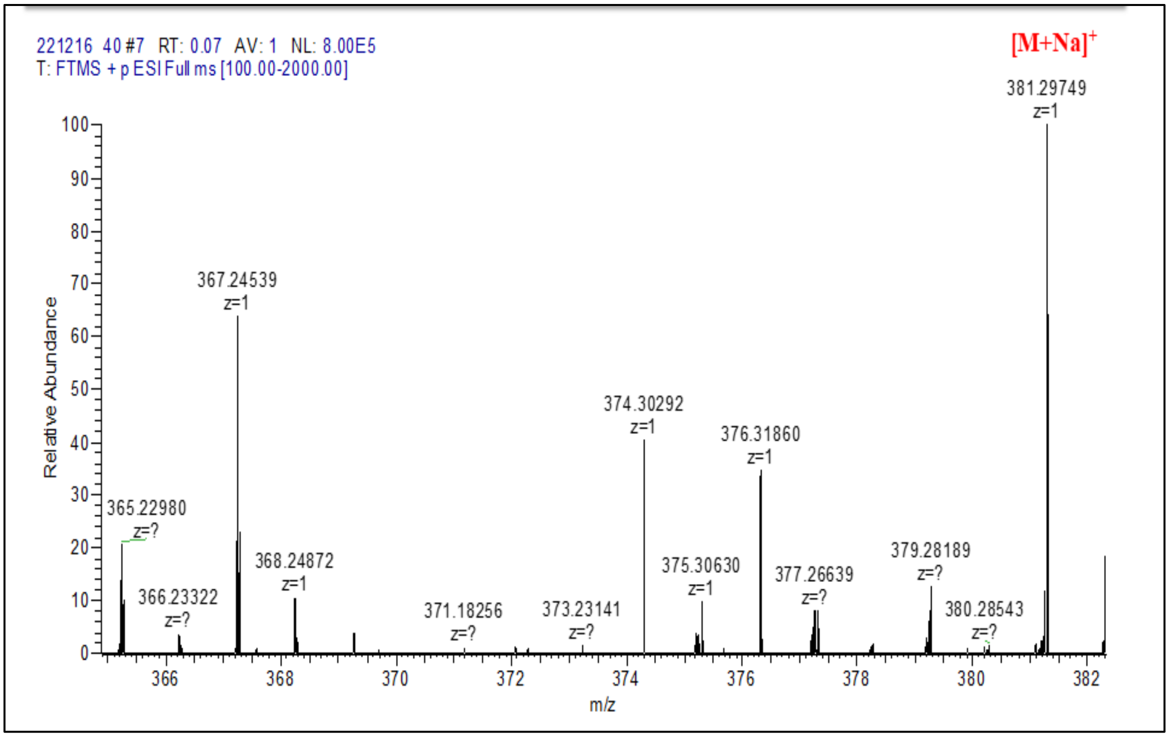

Fig. S20: Positive HR-ESI-MS of compound **5**

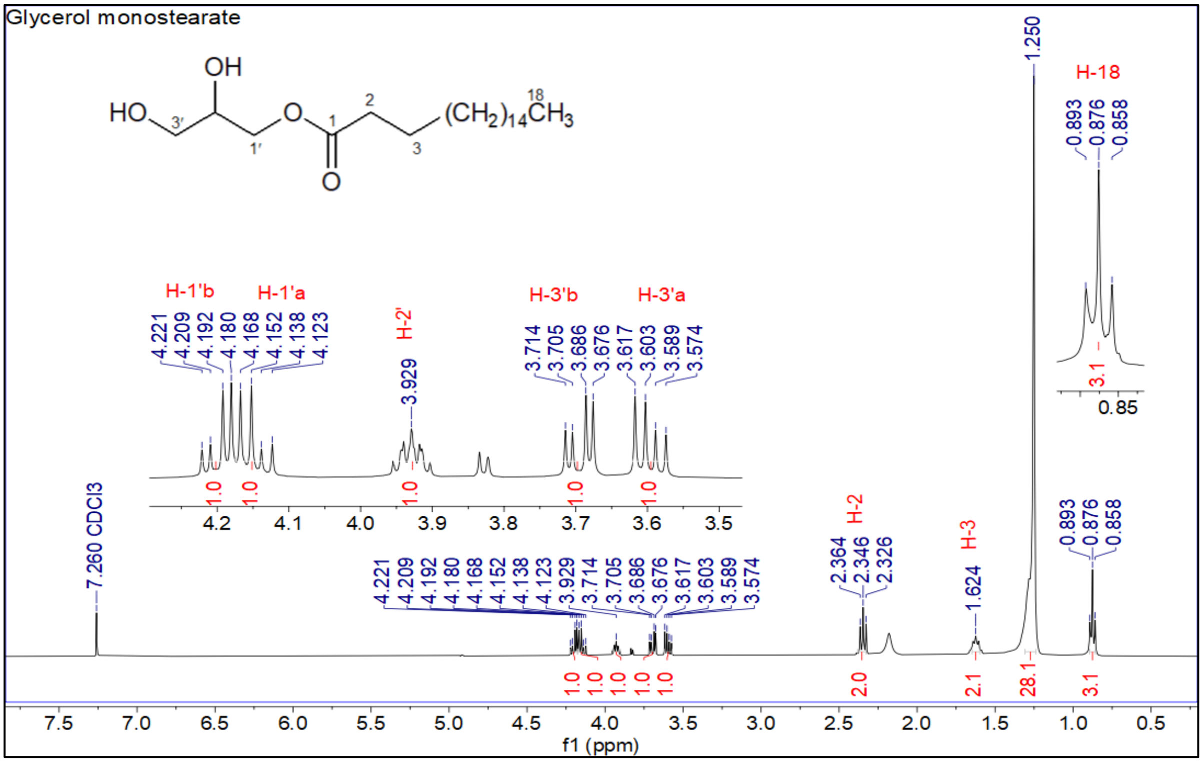

Fig. S21: ^{1}H NMR spectrum of compound **5** ($CDCl_3$, 400 MHz)

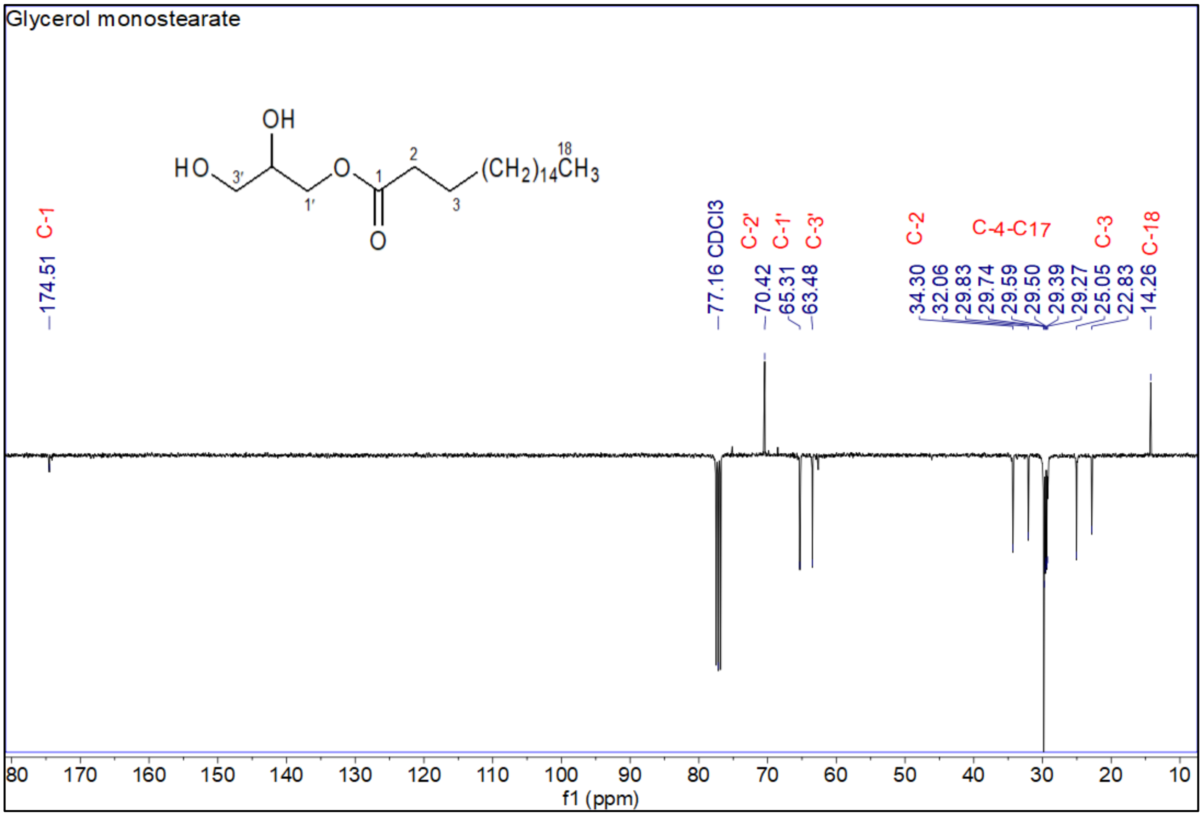

Fig. S22: ^{13}C (APT) NMR spectrum of compound **5** ($CDCl_3$, 100 MHz)

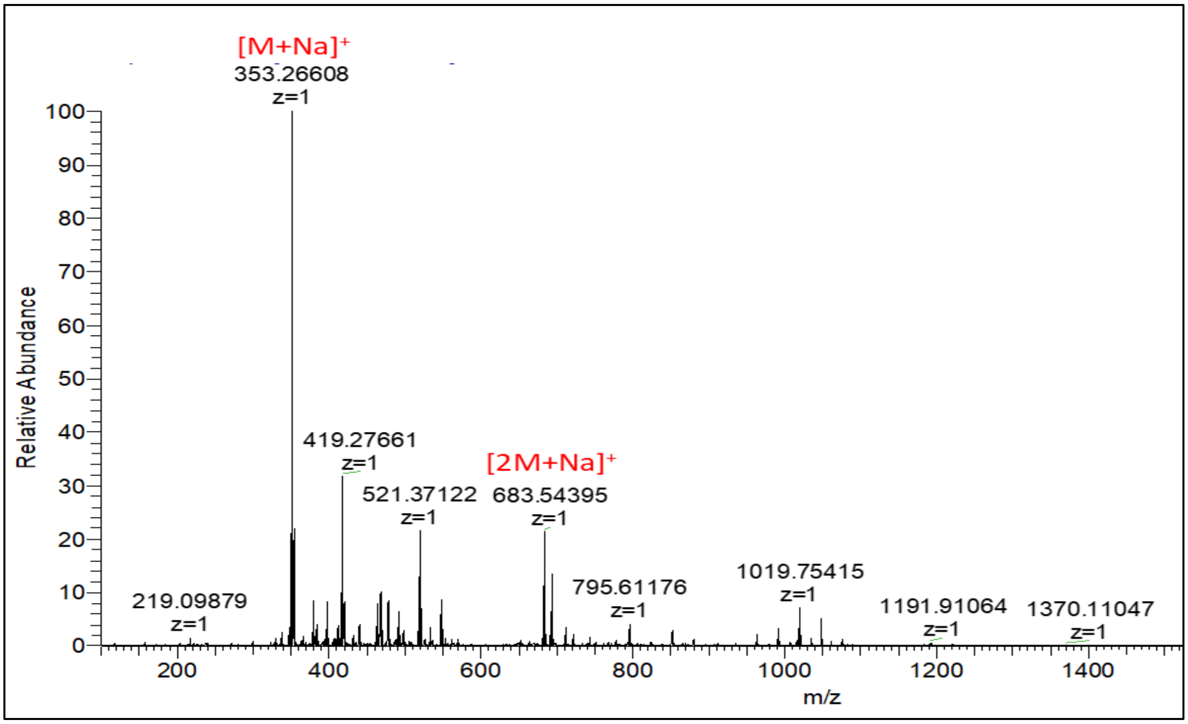

Fig. S23: Positive HR-ESI-MS of compound **6**

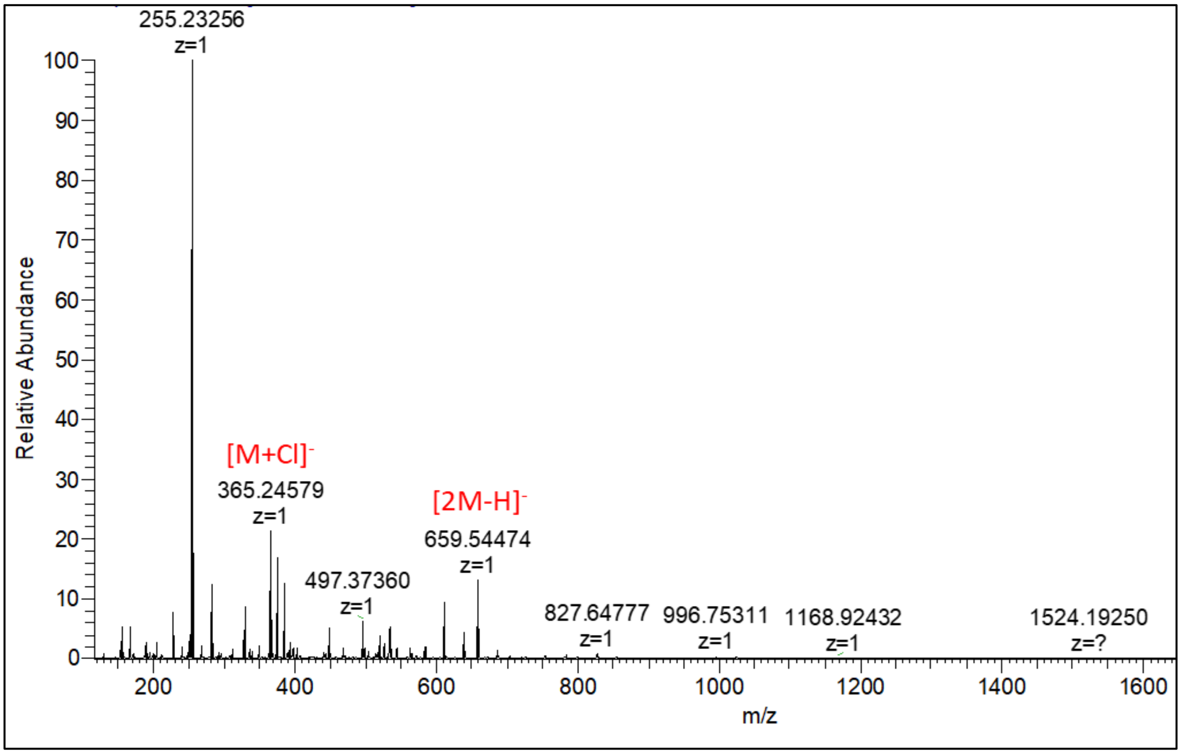

Fig. S24: Negative HR-ESI-MS of compound **6**

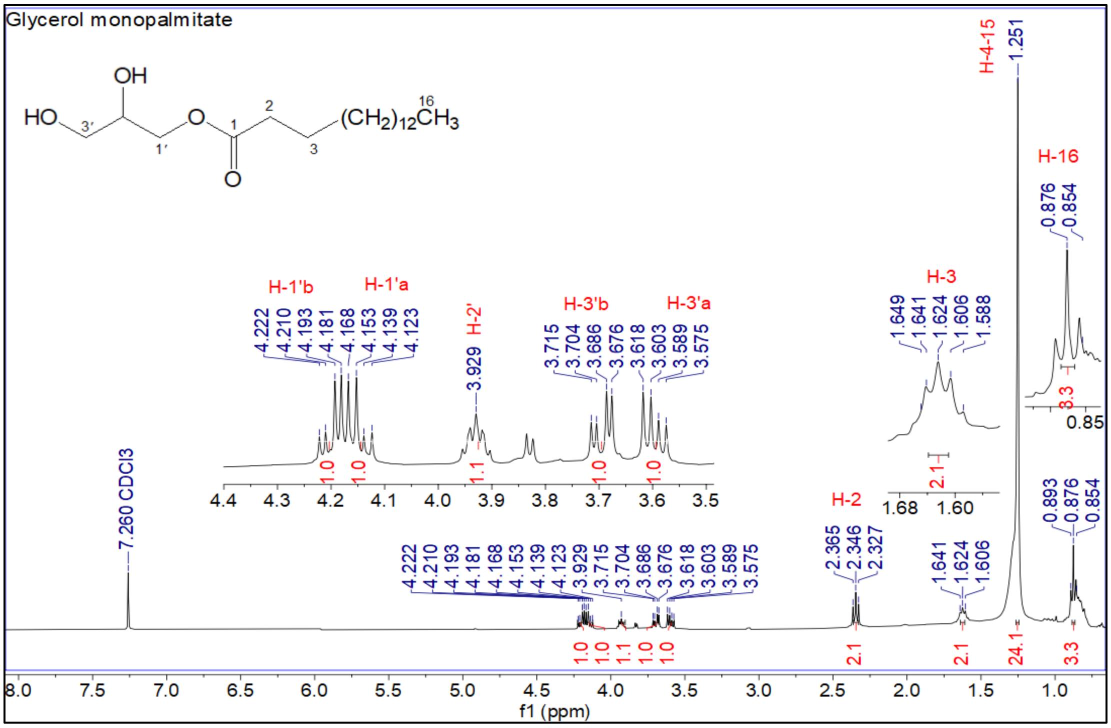

Fig. S25: ^{1}H NMR spectrum of compound **6** ($CDCl_3$, 400 MHz)

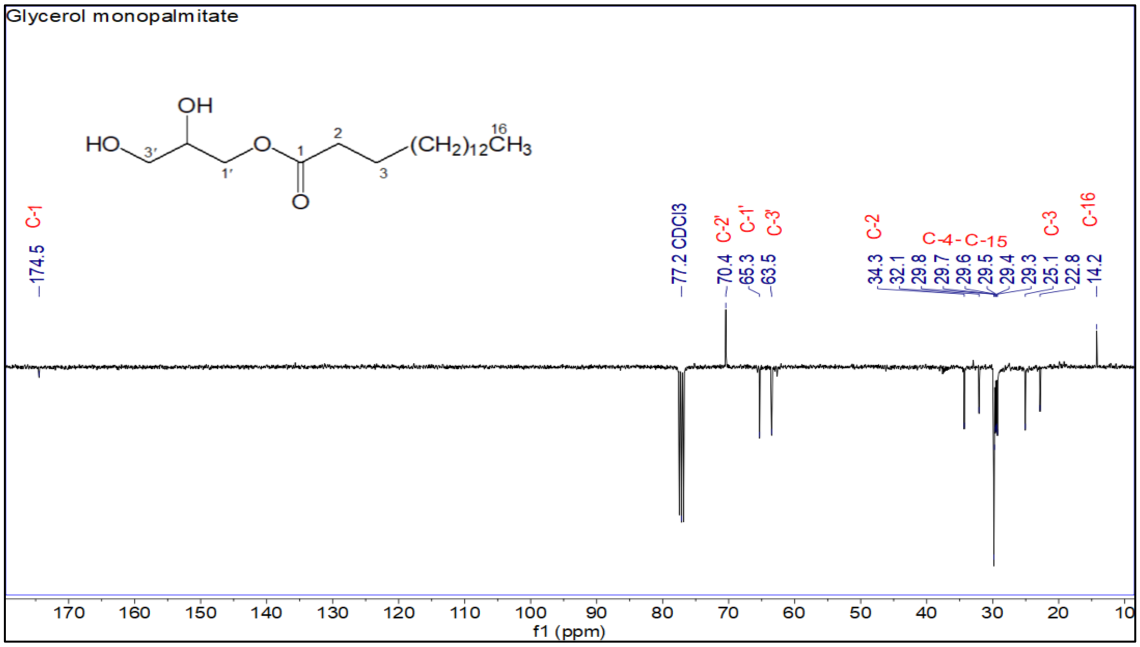

Fig. S26: ^{13}C (APT) NMR spectrum of compound **6** ($CDCl_3$, 100 MHz)

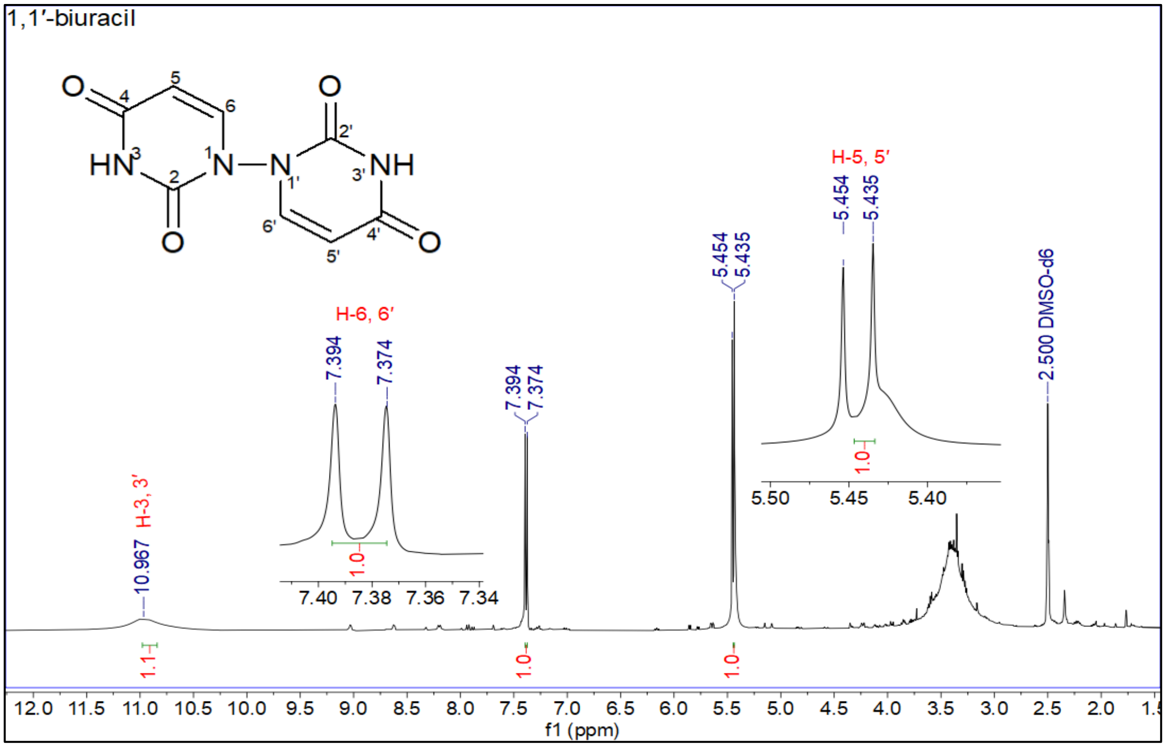

Fig. S27: ^{1}H NMR spectrum of compound **7** (DMSO-d_6, 400 MHz)

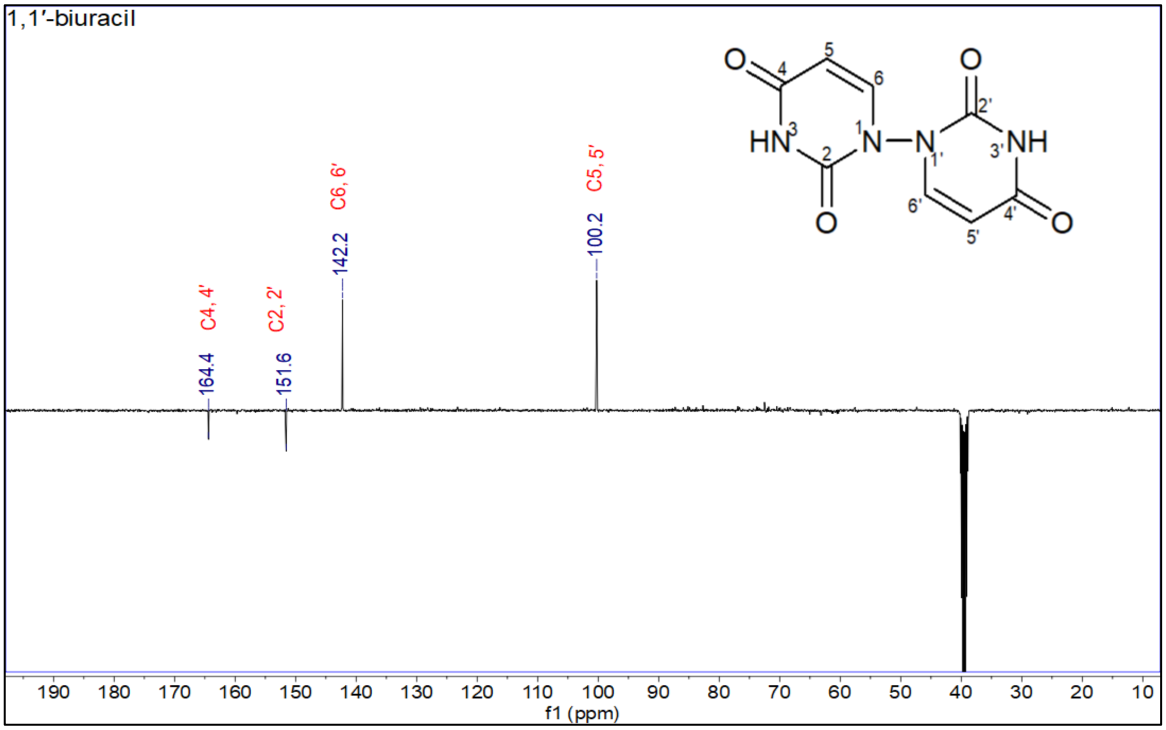

Fig. S28: ^{13}C (APT) NMR spectrum of compound **7** (DMSO-d_6, 400 MHz)

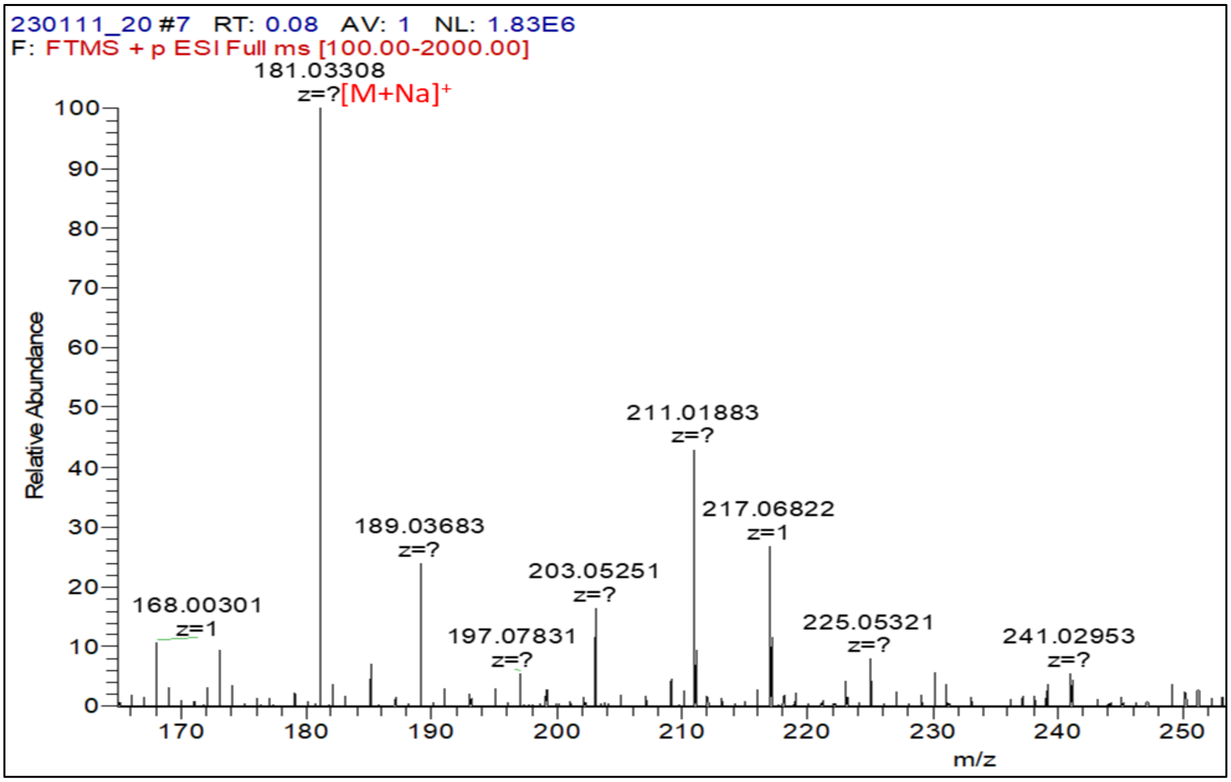

Fig. S29: Positive HR-ESI-MS of compound **8**

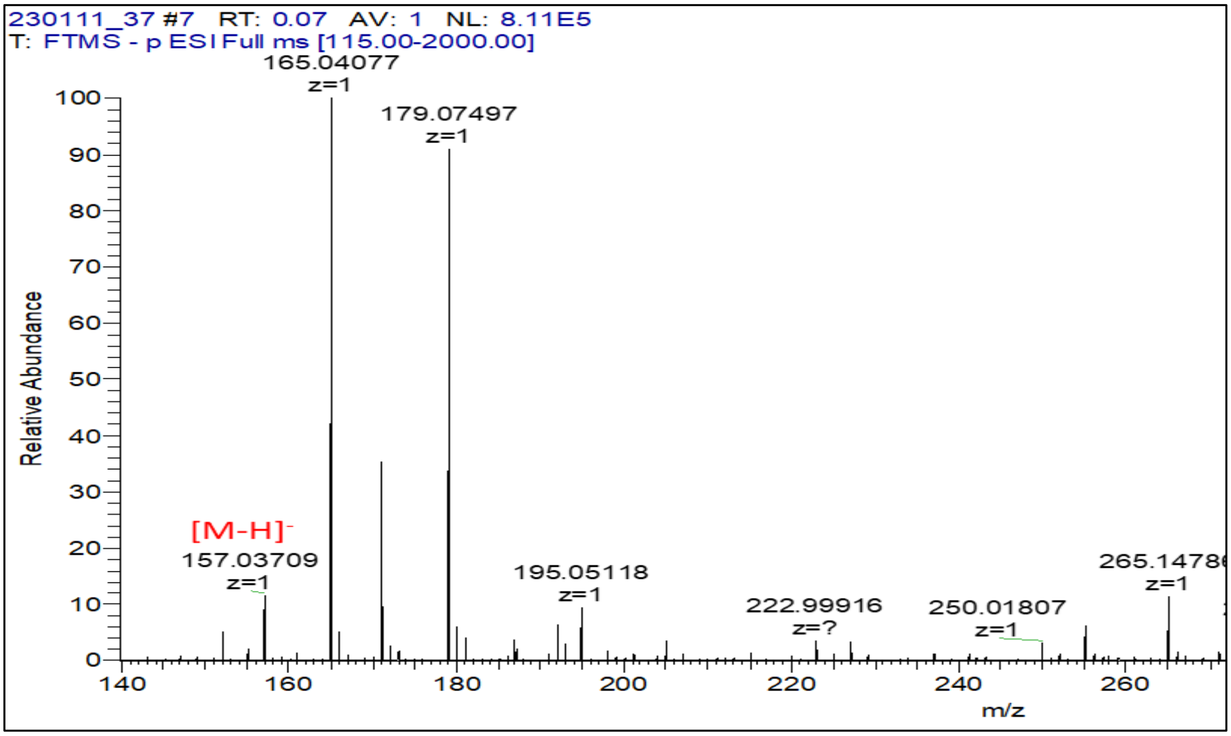

Fig. S30: Negative HR-ESI-MS of compound **8**

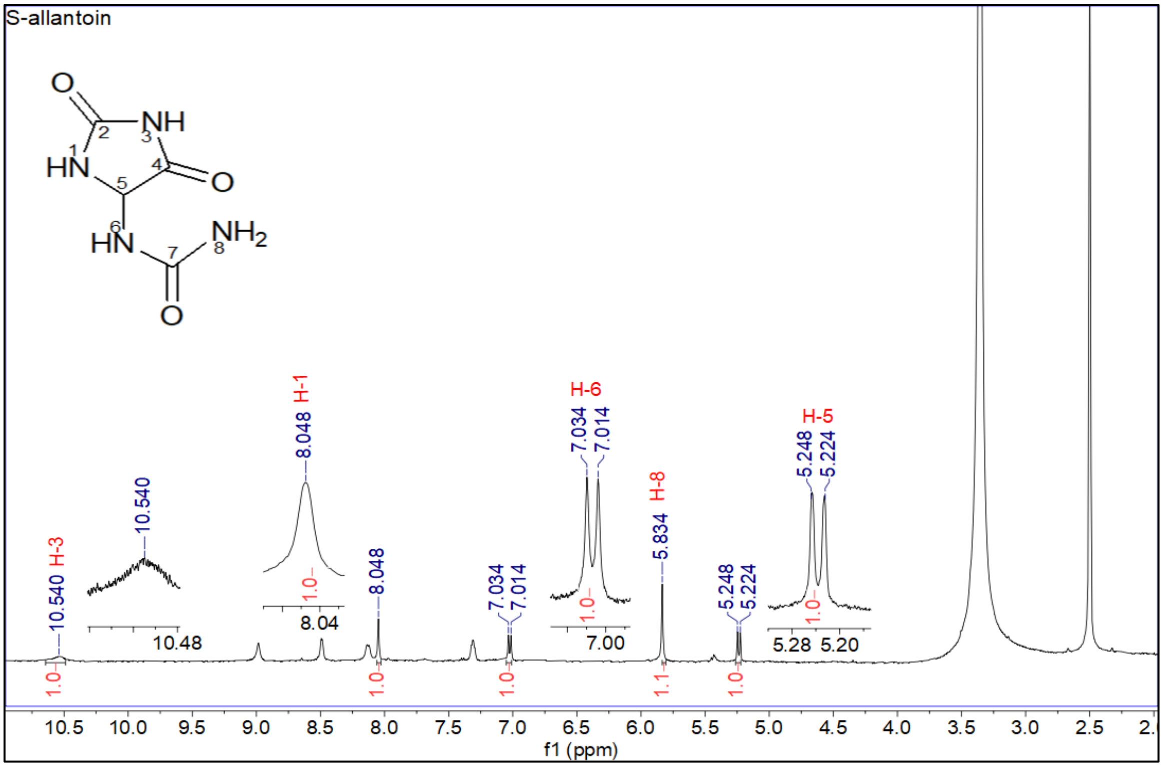

Fig. S31: ^{1}H NMR spectrum of compound **8** (DMSO-d_6, 400 MHz)

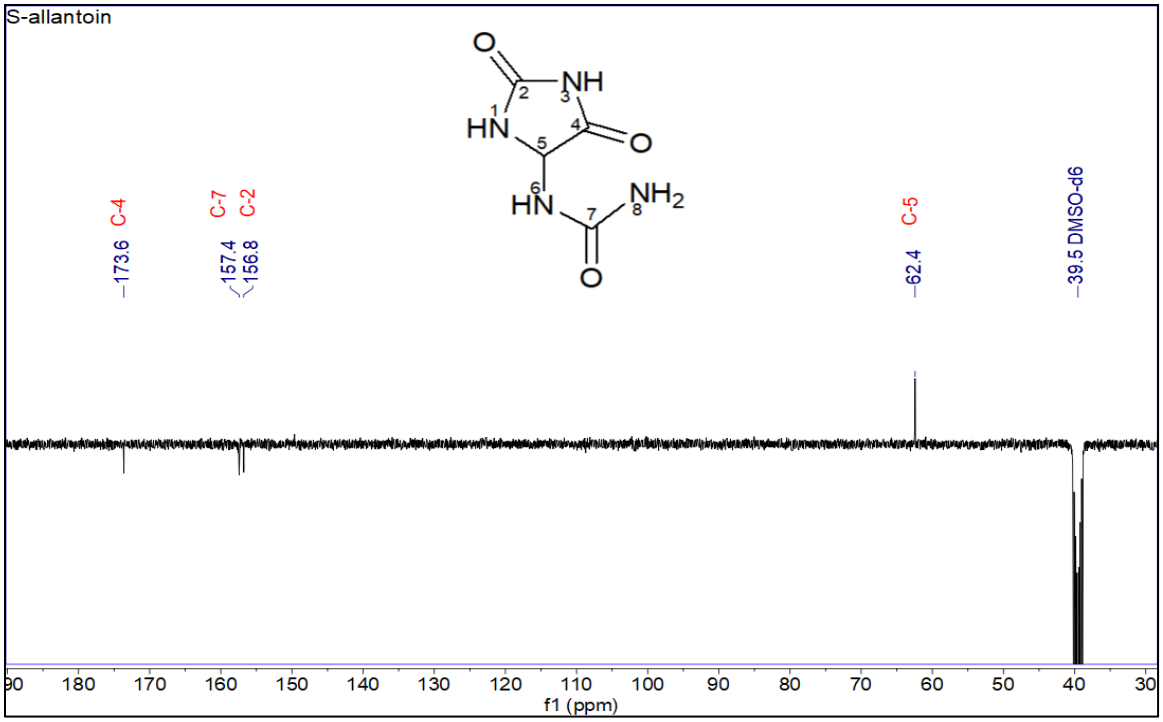

Fig. S32: ^{13}C (APT) NMR spectrum of compound **8** (DMSO-d_6, 100 MHz)

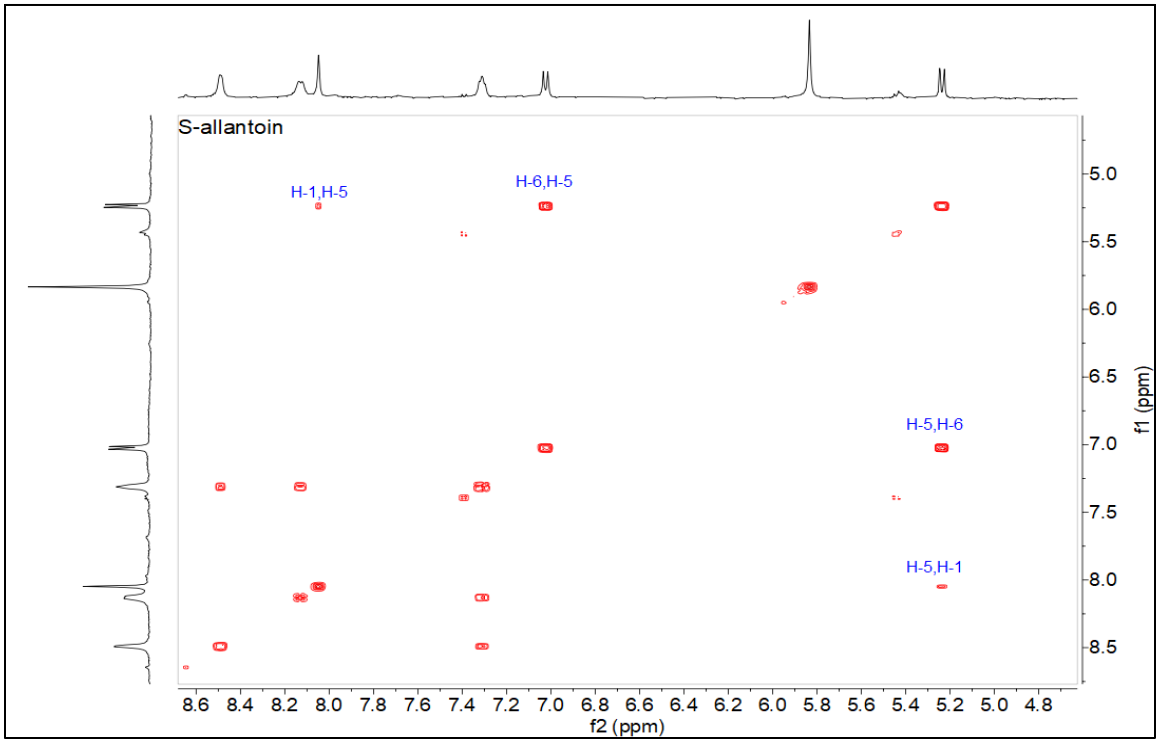

Fig. S33: ^{1}H-^{1}H COSY spectrum of compound **8** (DMSO-d_6, 400 MHz)

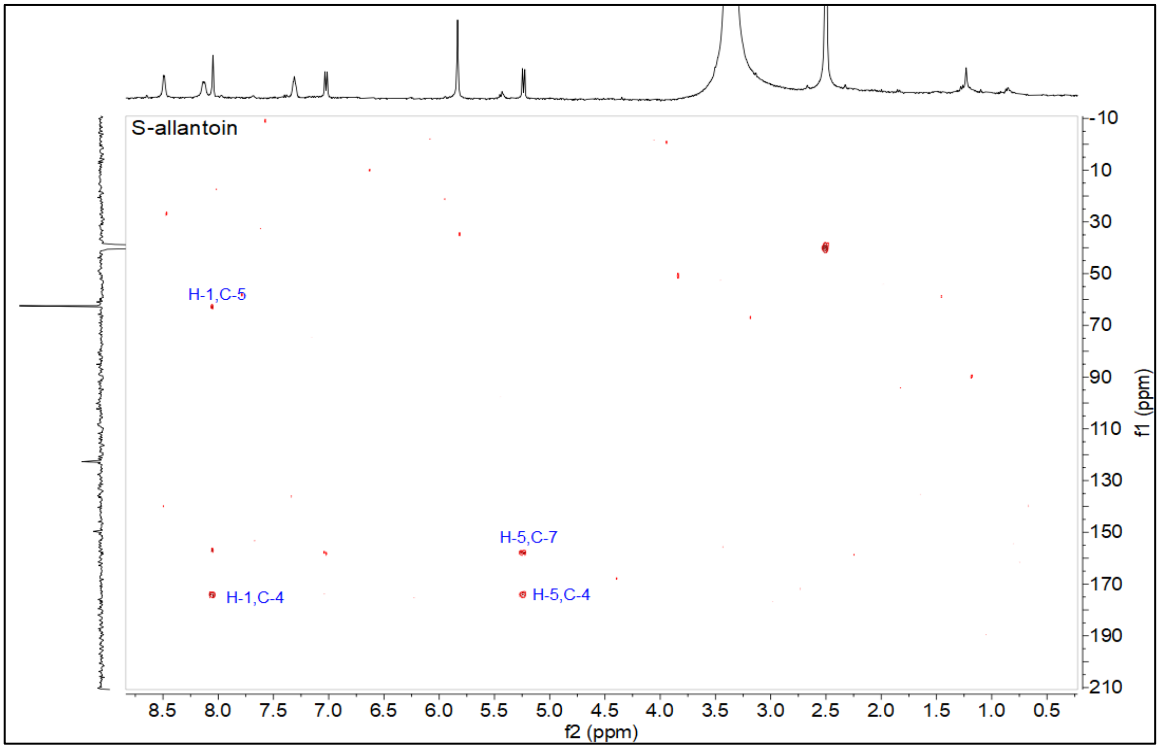

Fig. S34: HMBC spectrum of compound **8** (DMSO-d_6, 400/100 MHz)

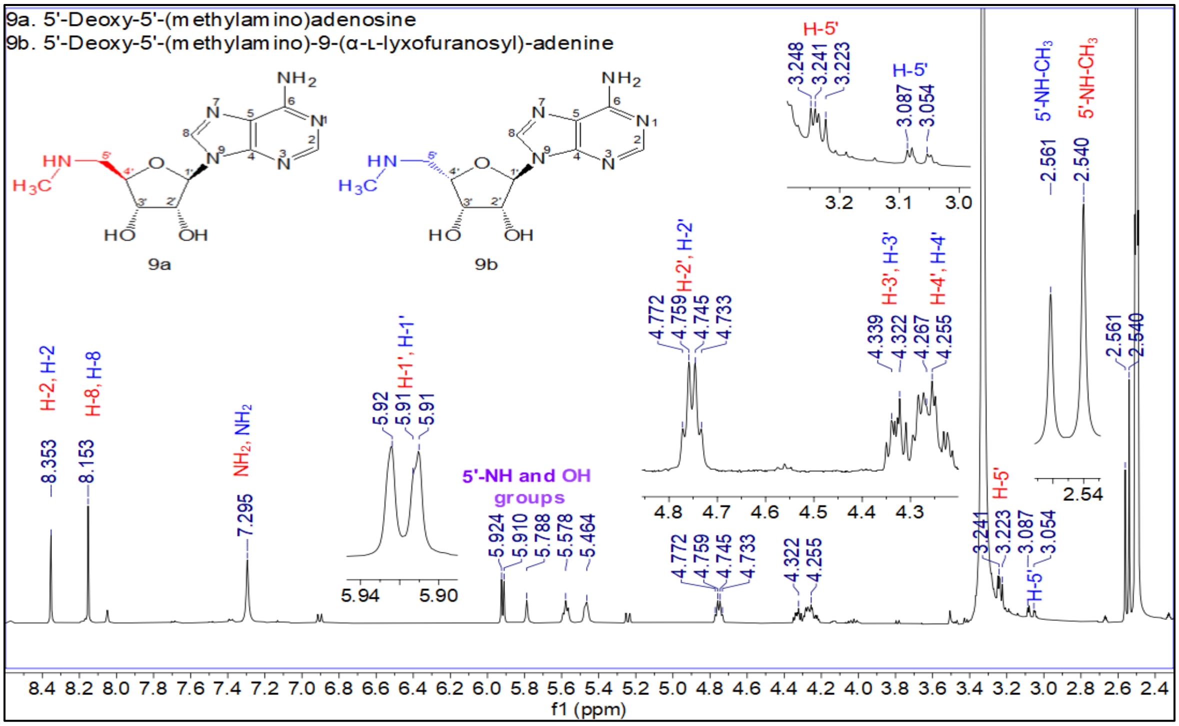

Fig. S35: ^{1}H NMR spectrum of compound **9** (DMSO-d_6, 400 MHz)

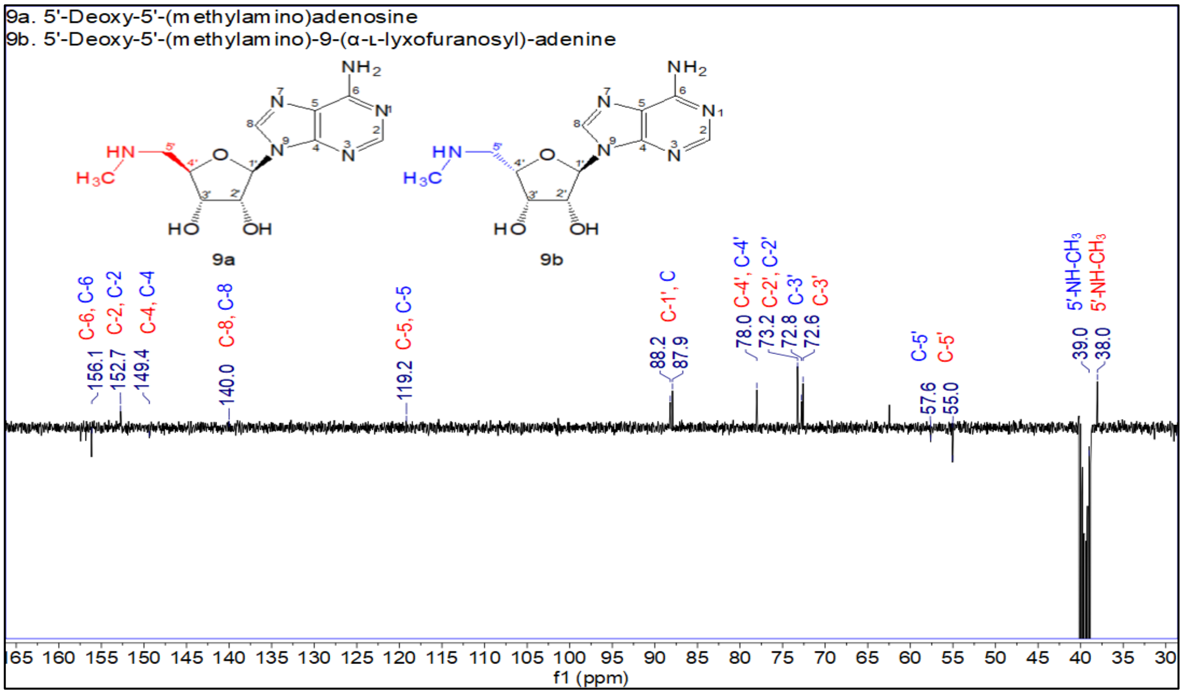

Fig. S36: ^{13}C (APT) NMR spectrum of compound **9** (DMSO-d_6, 100 MHz)

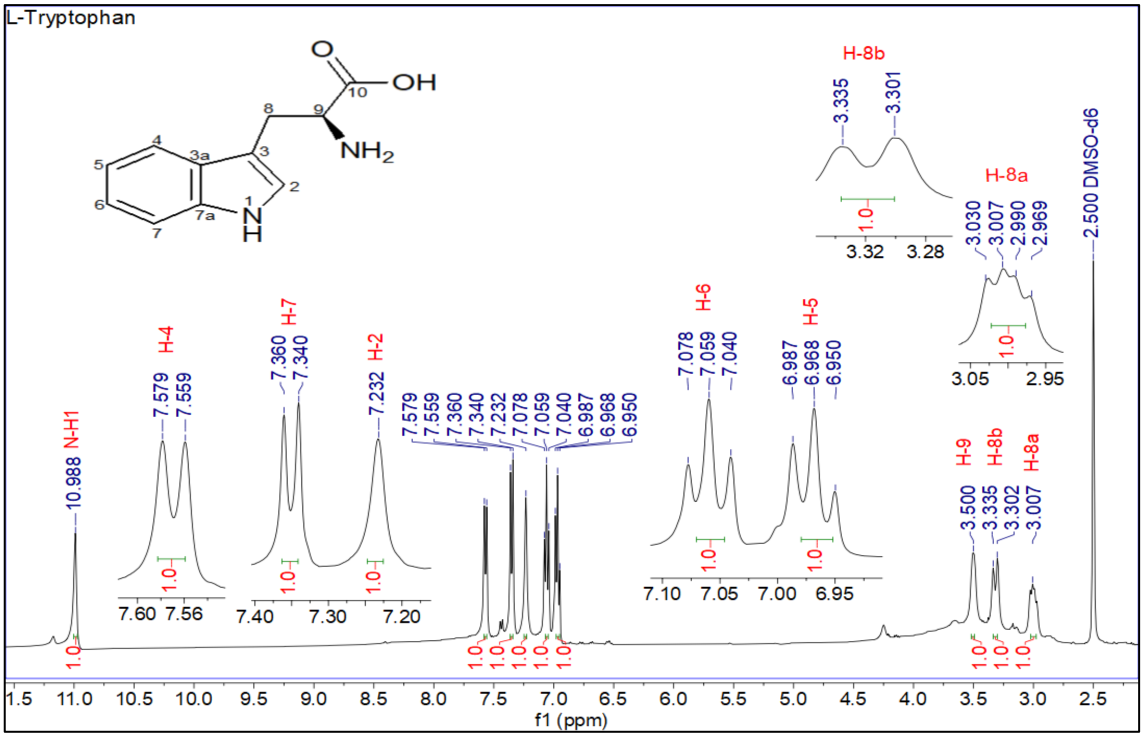

Fig. S37 : ^{1}H NMR spectrum of compound **10** (DMSO-d_6, 400 MHz)

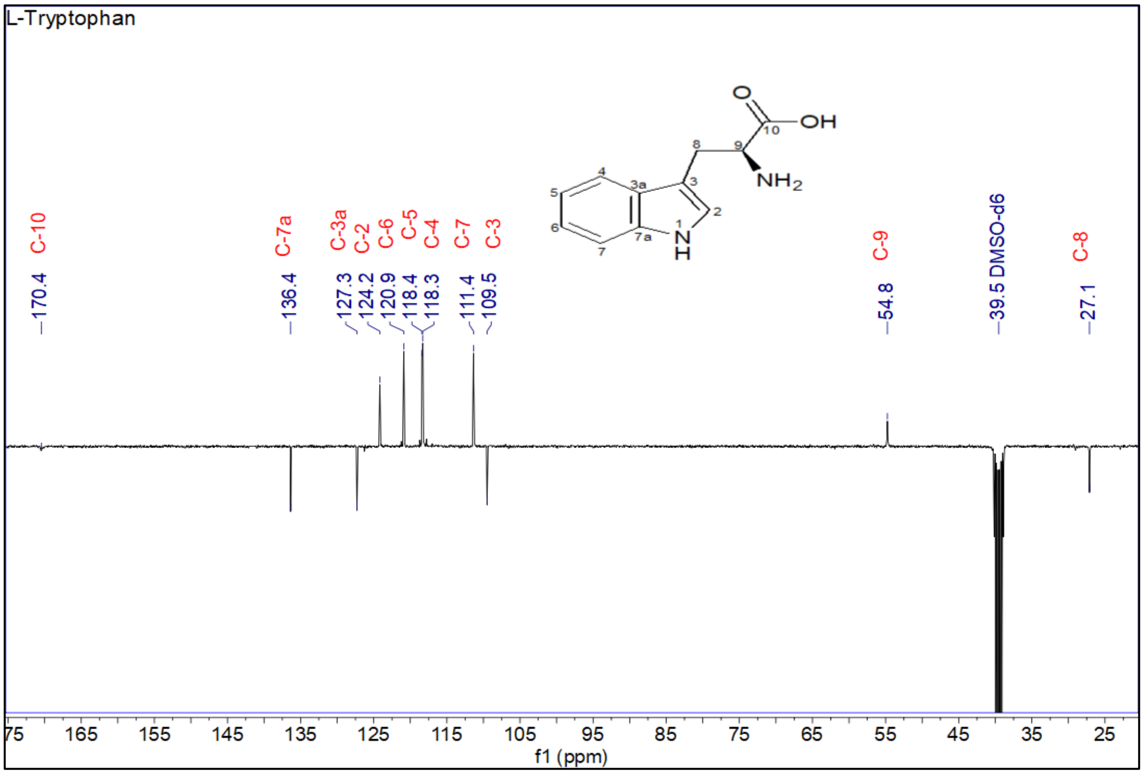

Fig. S38: ^{13}C (APT) NMR spectrum of compound **10** (DMSO-d_6, 100 MHz)

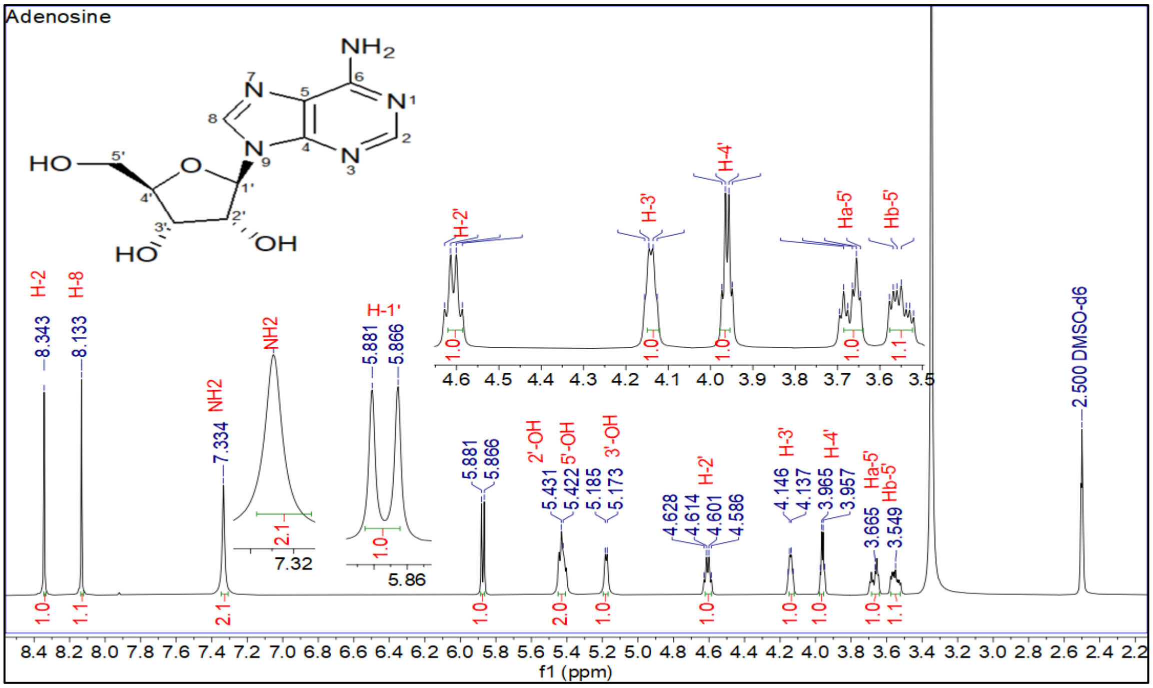

Fig. S39: ^{1}H NMR spectrum of compound **11** (DMSO-d_6, 400 MHz)

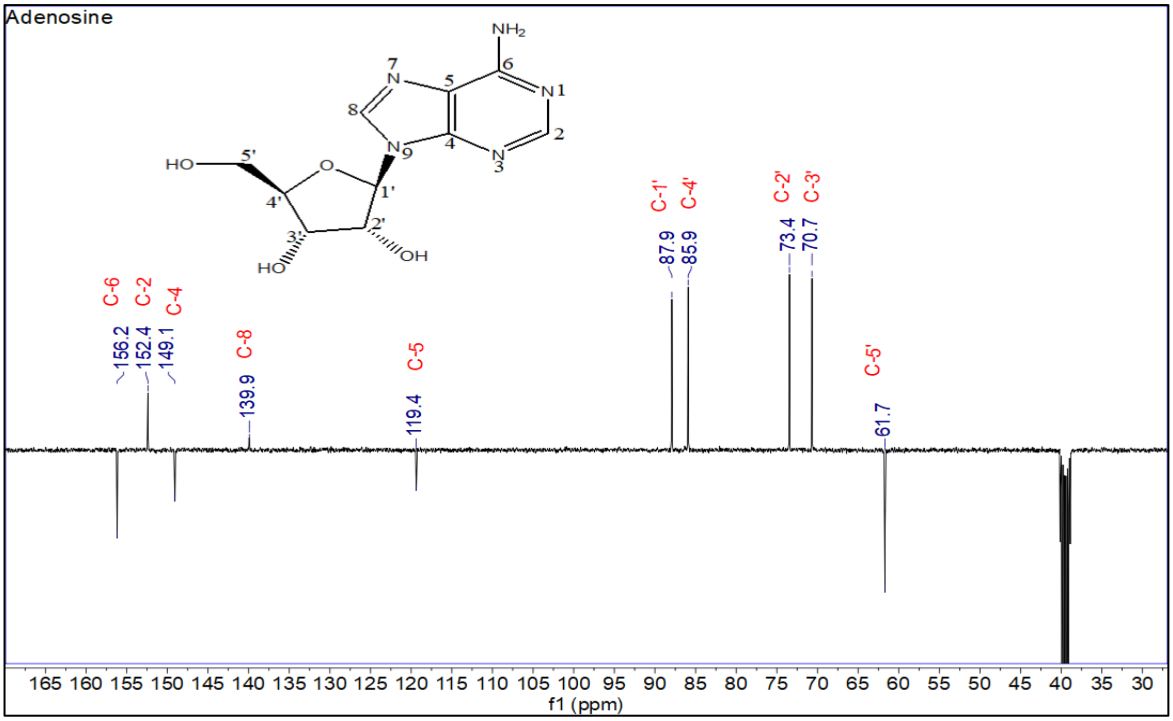

Fig. S40: ^{13}C (APT) NMR spectrum of compound **11** (DMSO-d_6, 100 MHz)

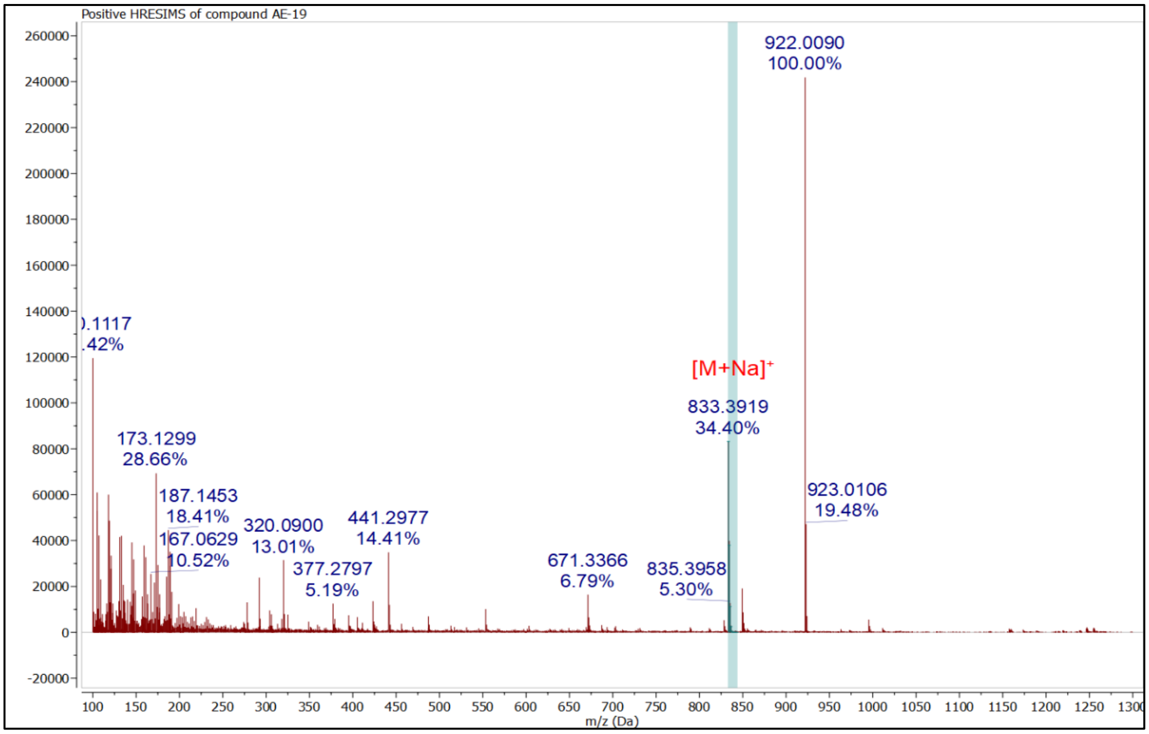

Fig. S41: Positive HR-ESI-MS of compound **12**

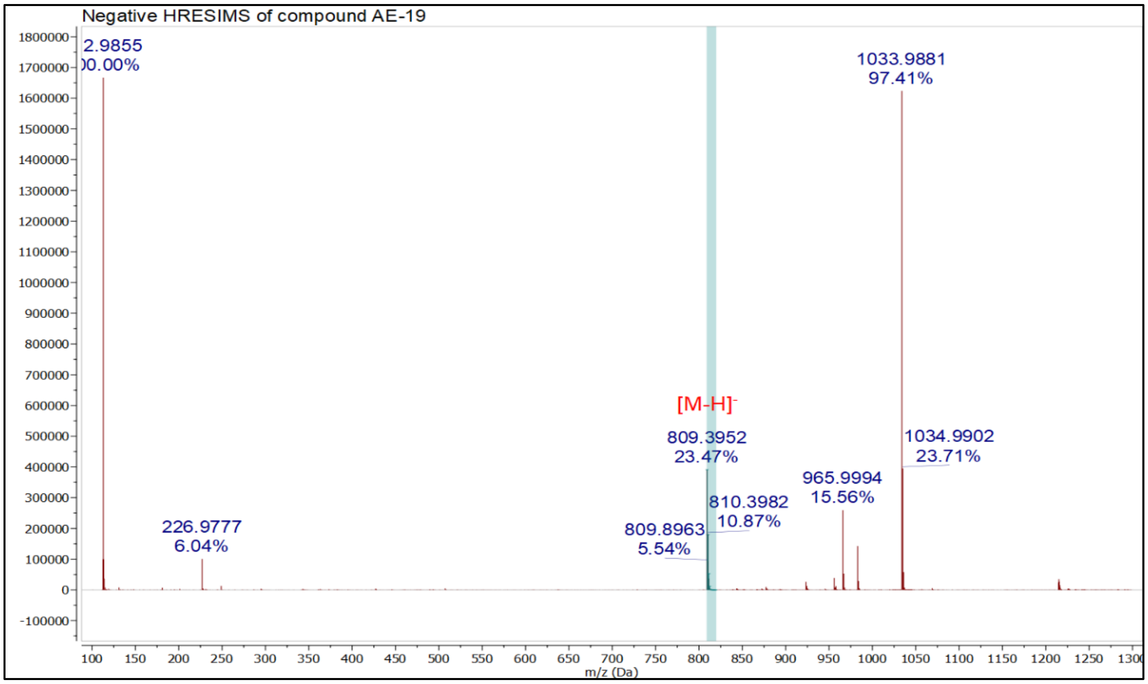

Fig. S42: Negative HR-ESI-MS of compound **12**

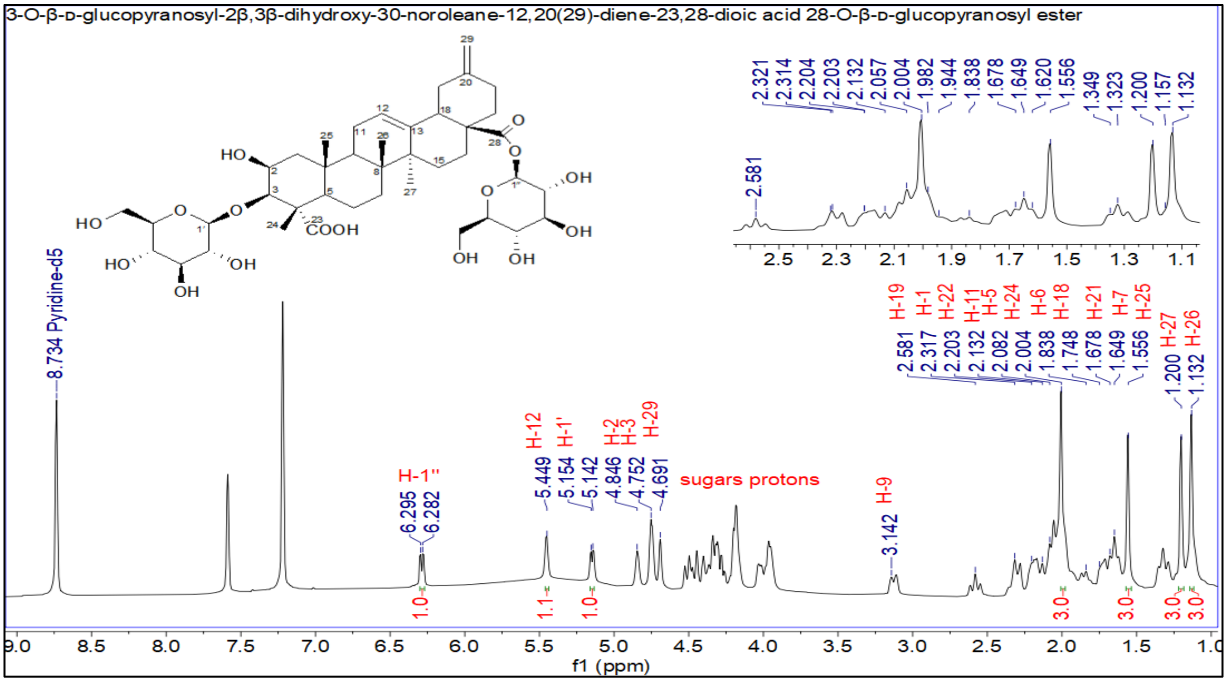

Fig. S43: ^{1}H NMR spectrum of compound **12** (C_5D_5N, 400 MHz)

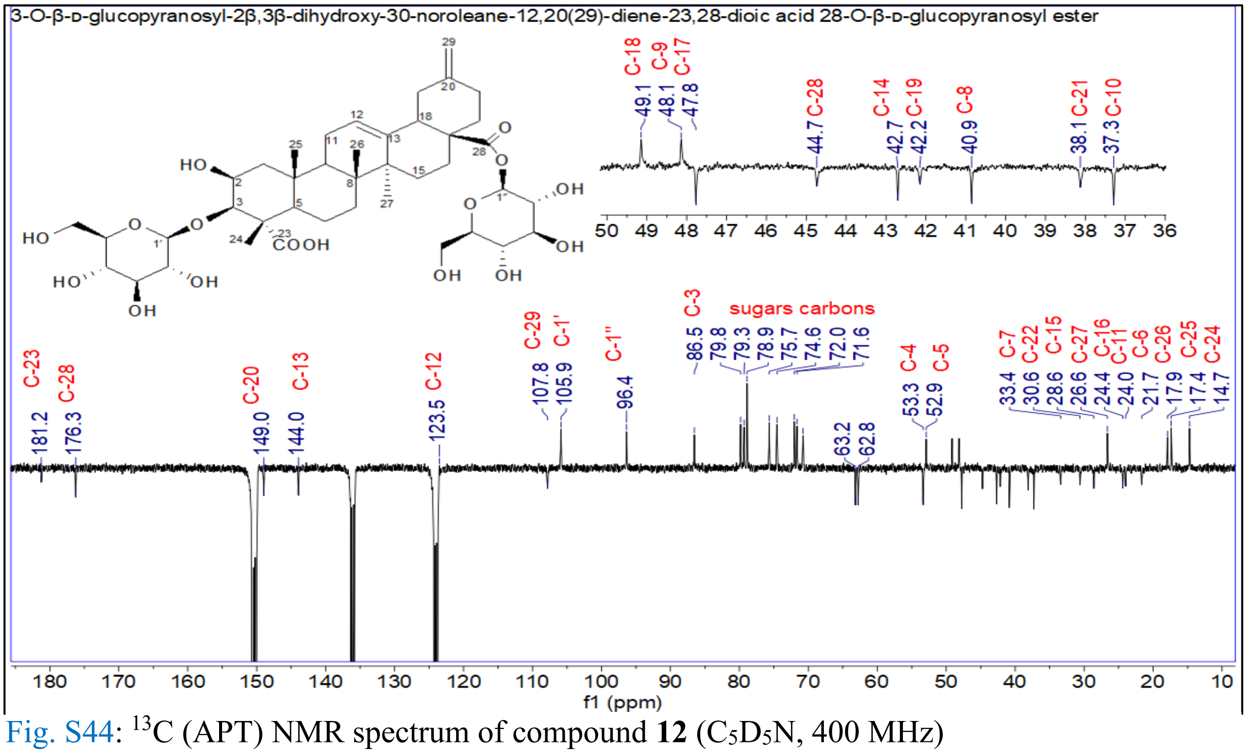

Fig. S44: ^{13}C (APT) NMR spectrum of compound **12** (C_5D_5N, 400 MHz)

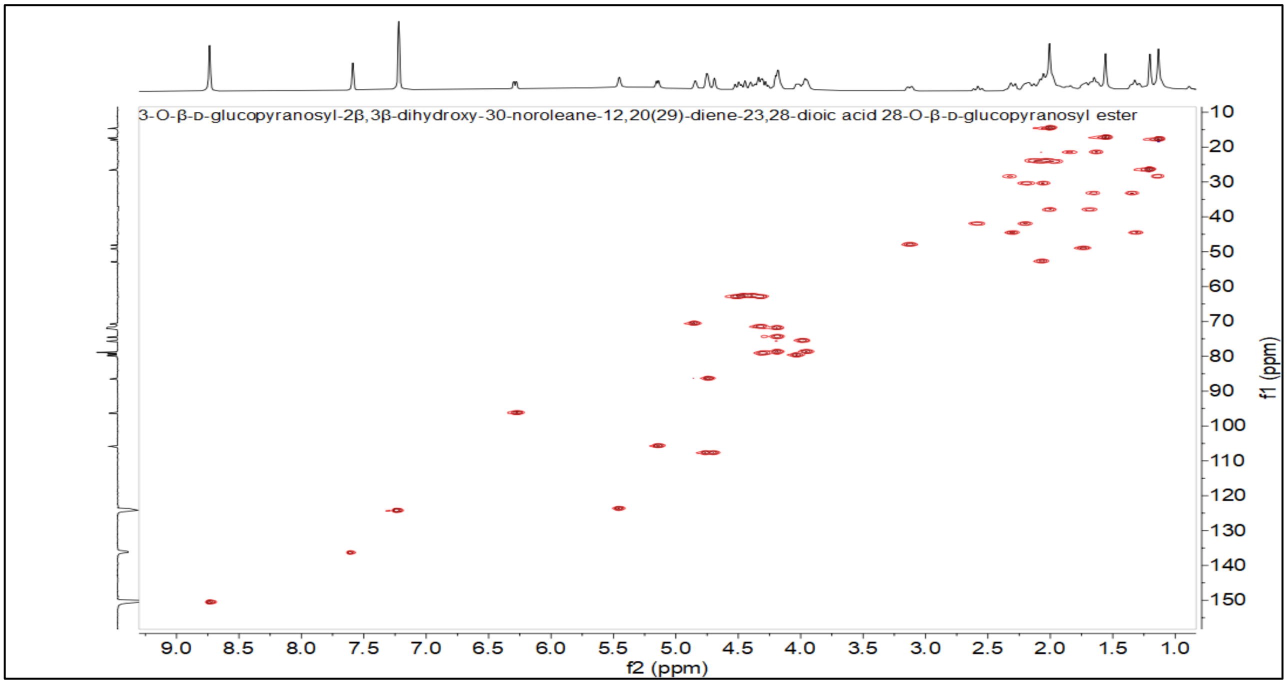

Fig. S45: HSQC spectrum of compound **12** (C_5D_5N, 400/100 MHz)

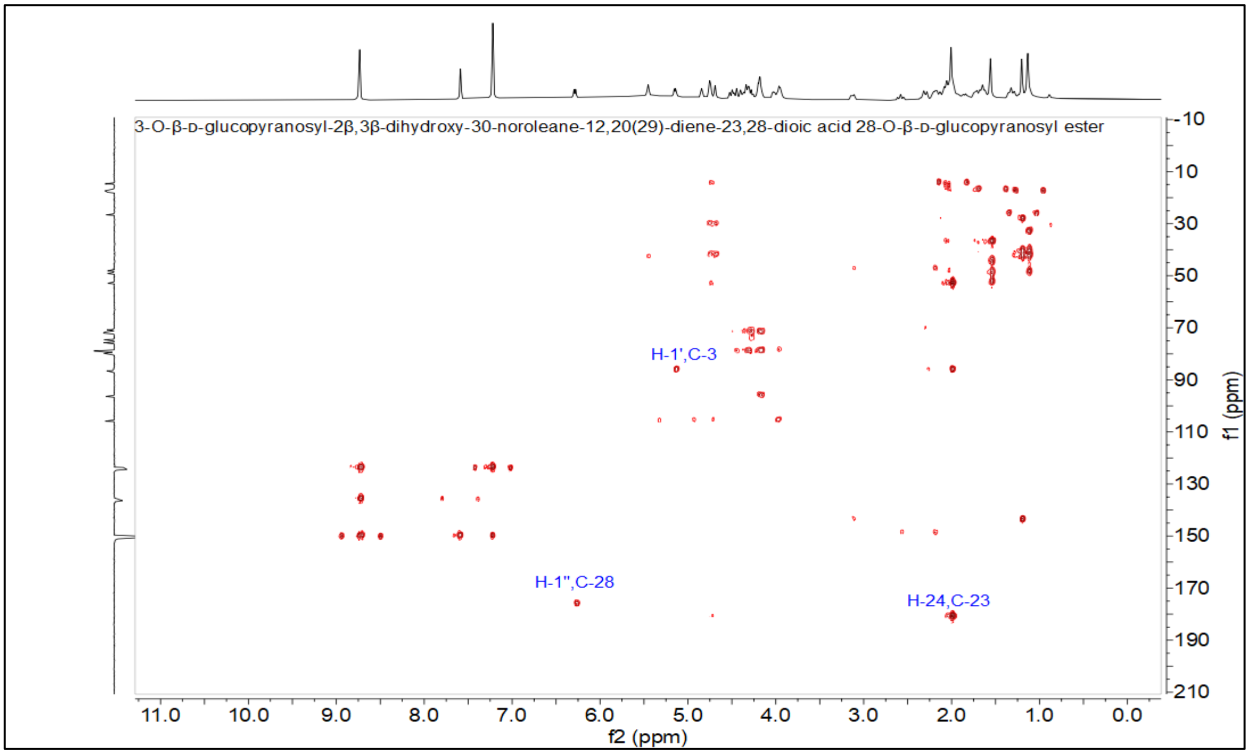

Fig. S46: HMBC spectrum of compound **12** (C_5D_5N, 400/100 MHz)

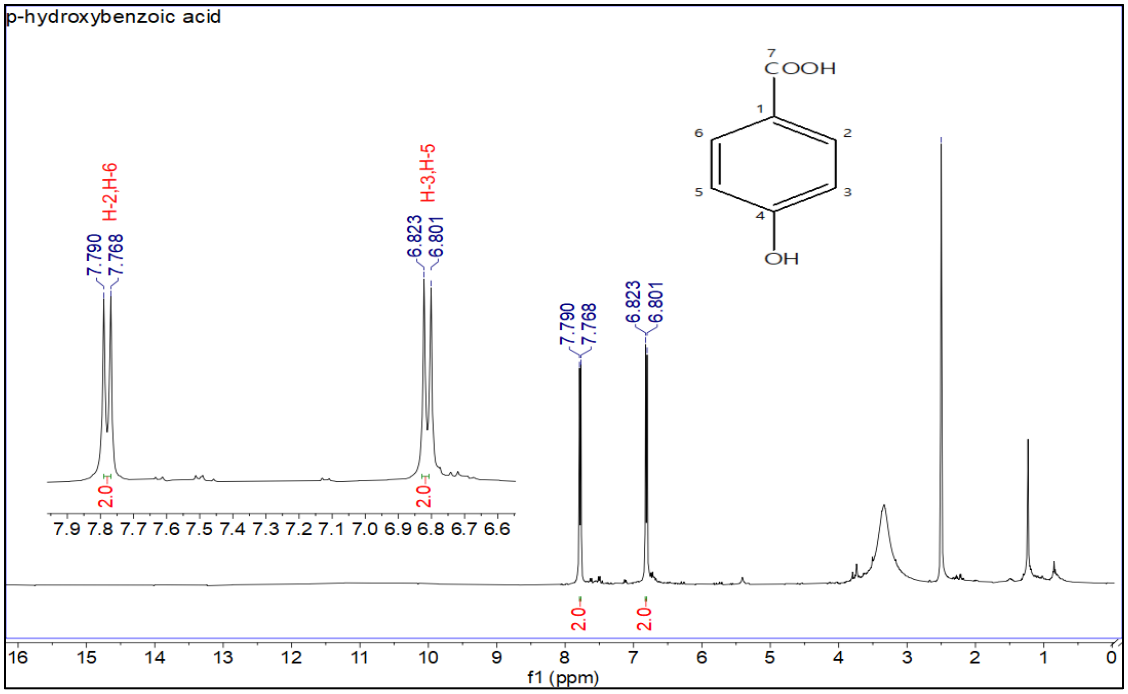

Fig. S47: ^{1}H NMR spectrum of compound **13** (DMSO-d_6, 400 MHz)

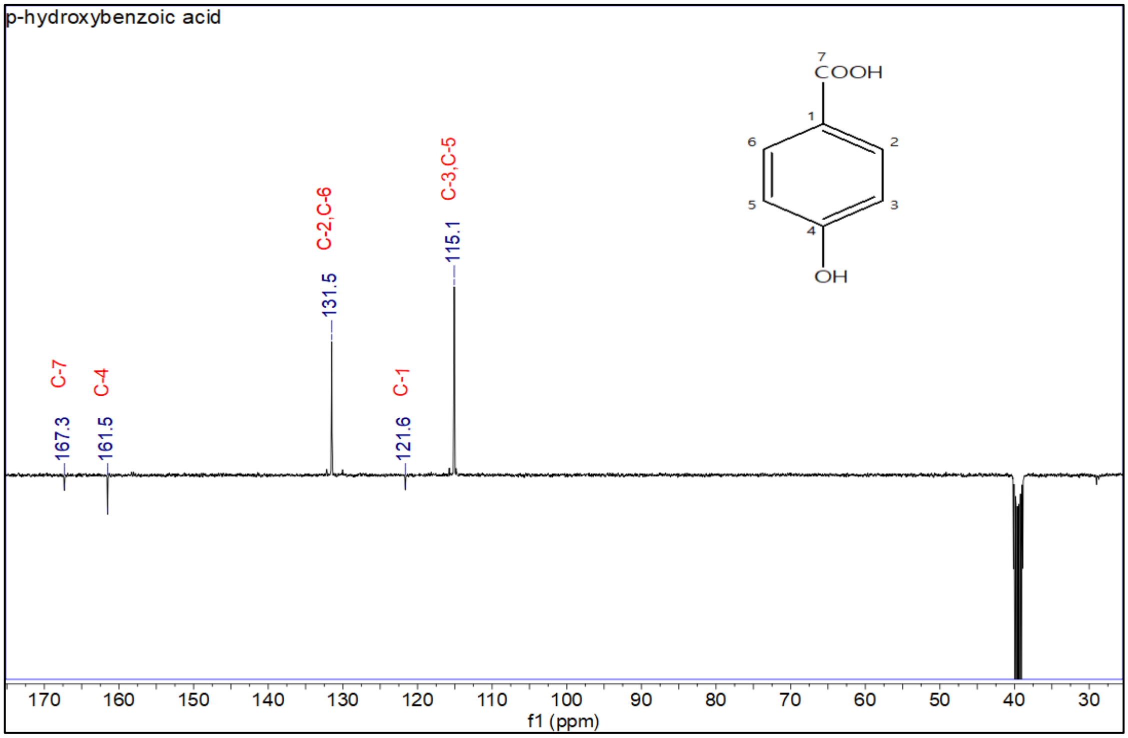

Fig. S48: ^{13}C (APT) NMR spectrum of compound **13** (DMSO-d_6, 100 MHz)

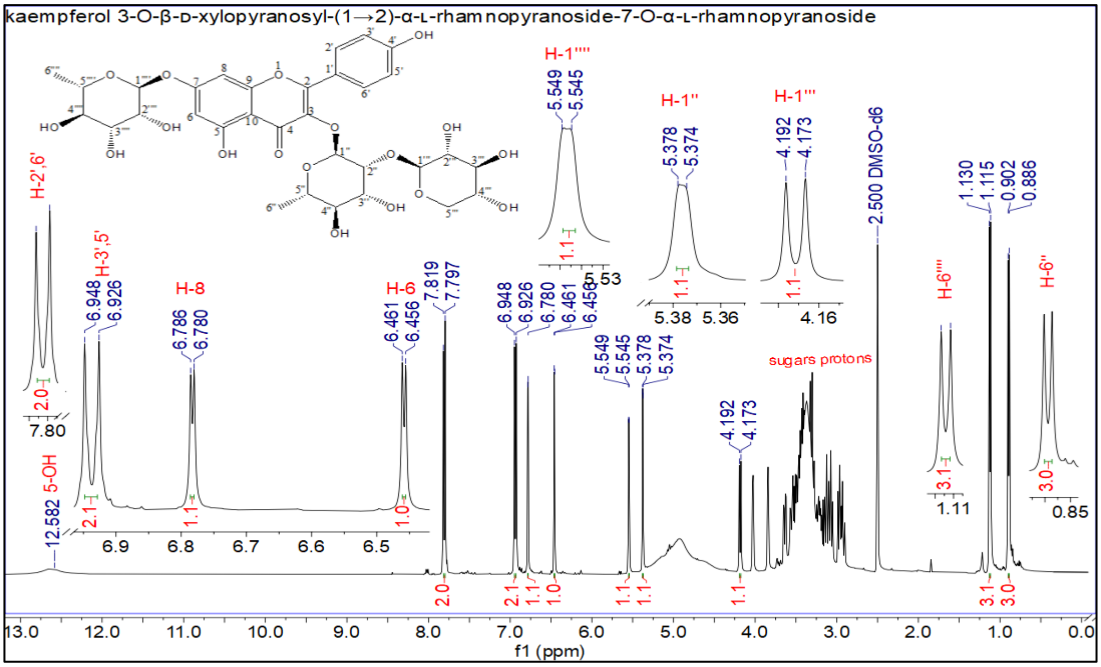

Fig. S49: ^{1}H NMR spectrum of compound **14** (DMSO-d_6, 400 MHz)

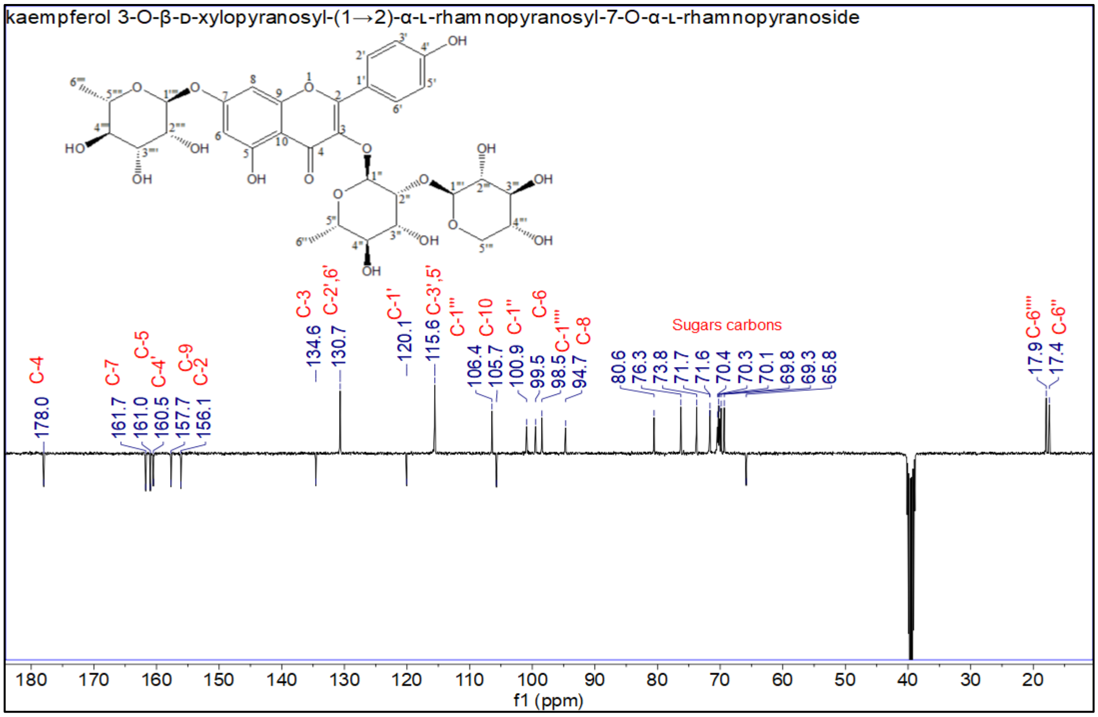

Fig. S50: ^{13}C (APT) NMR spectrum of compound **14** (DMSO-d_6, 100 MHz)

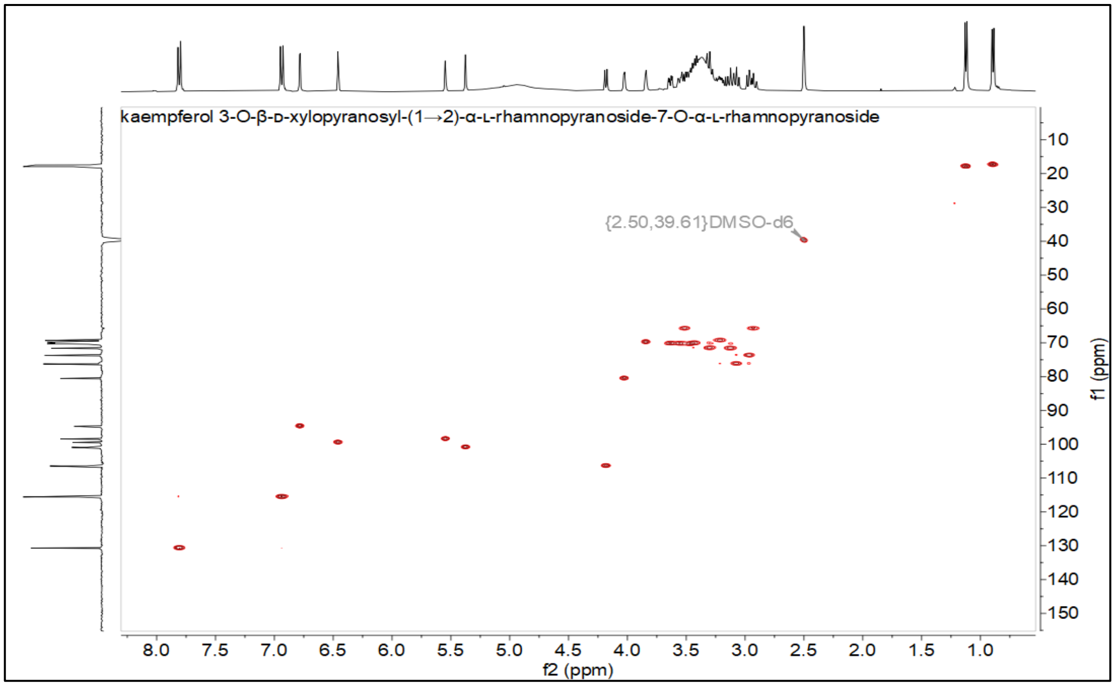

Fig. S51: HSQC spectrum of compound **14** (DMSO-d_6, 400/100 MHz)

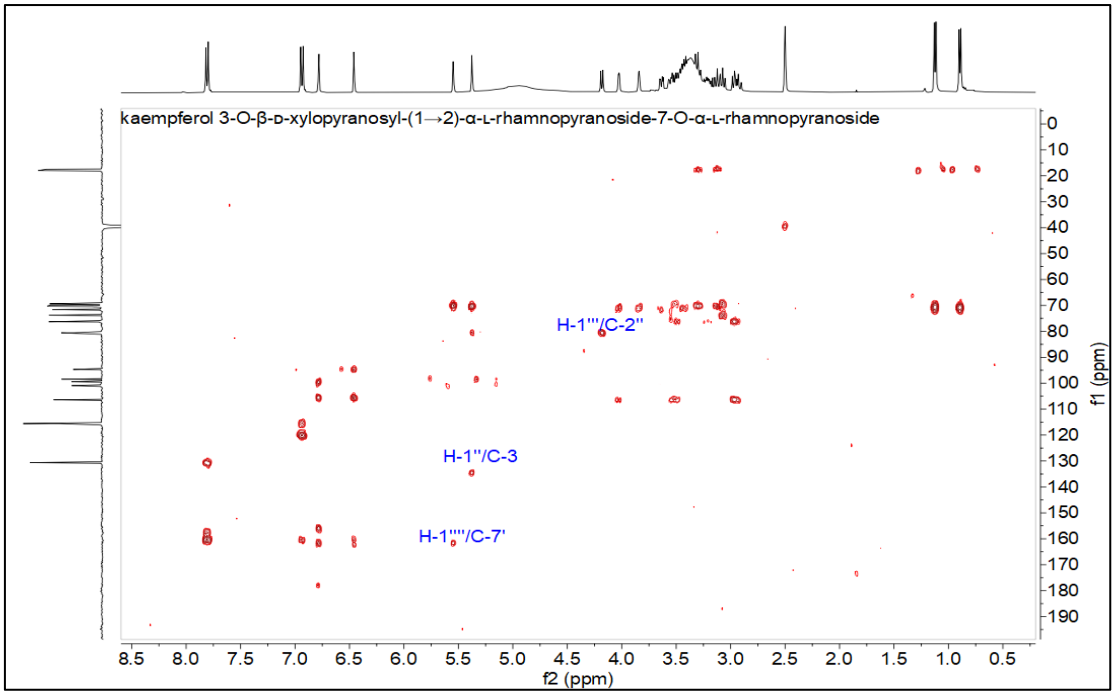

Fig. S52: HMBC spectrum of compound **14** (DMSO-d_6, 400/100 MHz)

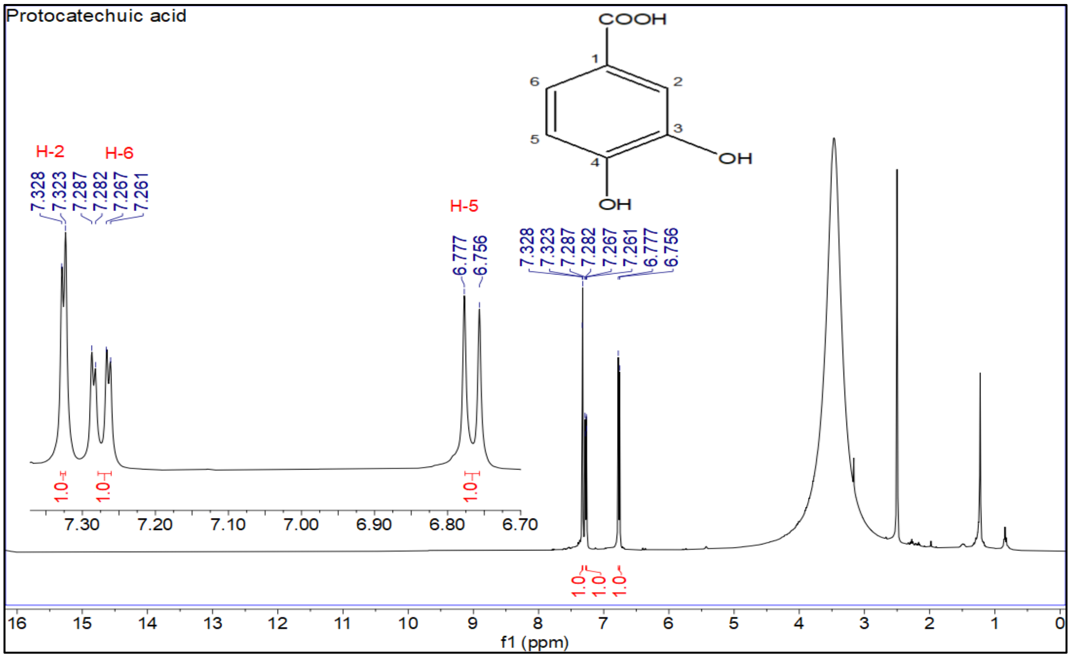

Fig. S53: ^{1}H NMR spectrum of compound **15** (DMSO-d_6, 400 MHz)

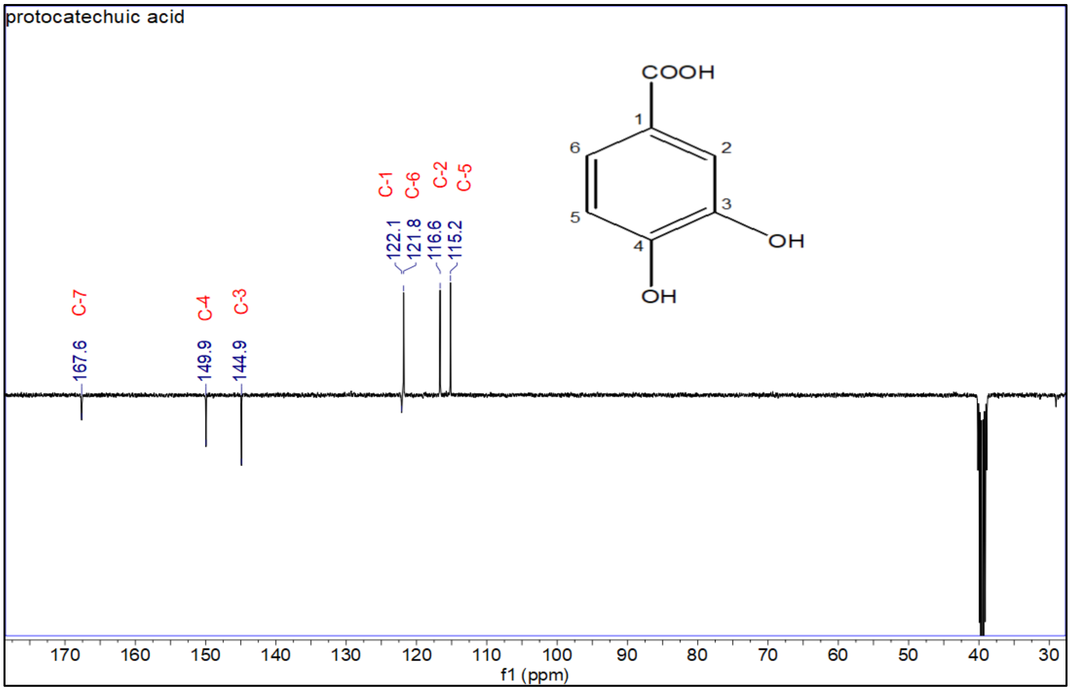

Fig. S54: ^{13}C (APT) NMR spectrum of compound **15** (DMSO-d_6, 100 MHz)

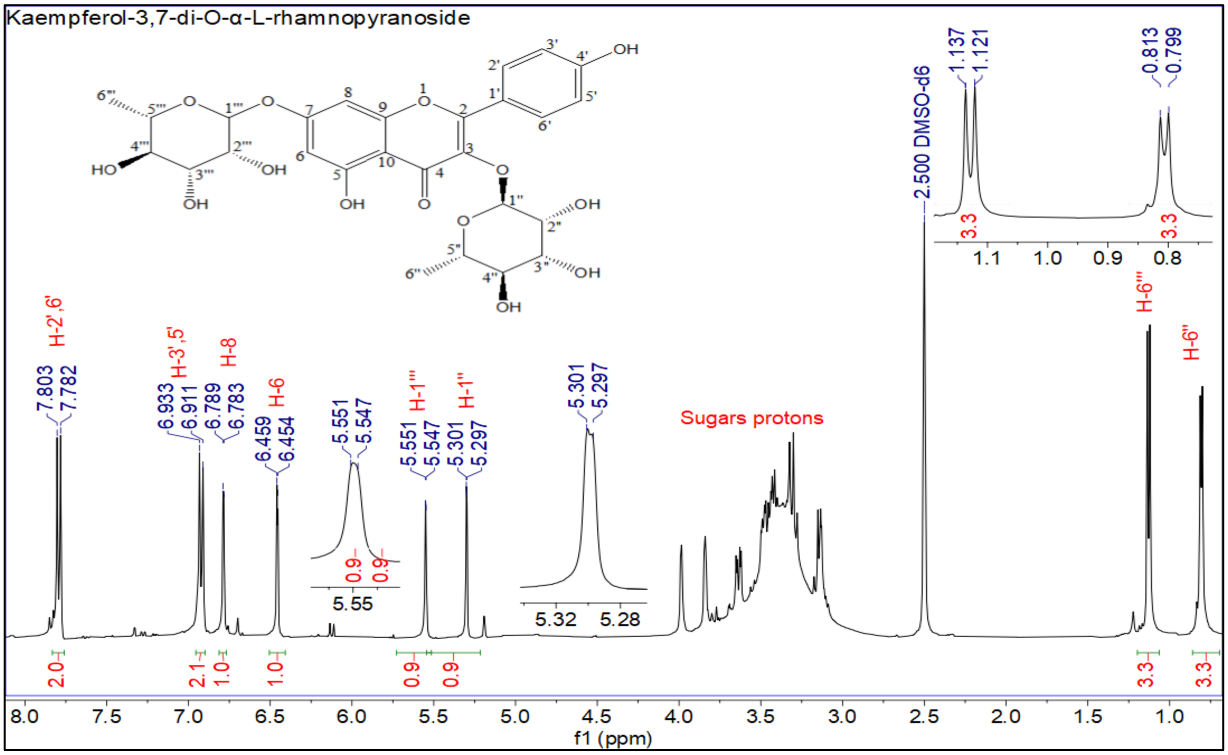

Fig. S55: ^{1}H NMR spectrum of compound **16** (DMSO-d_6, 400 MHz)

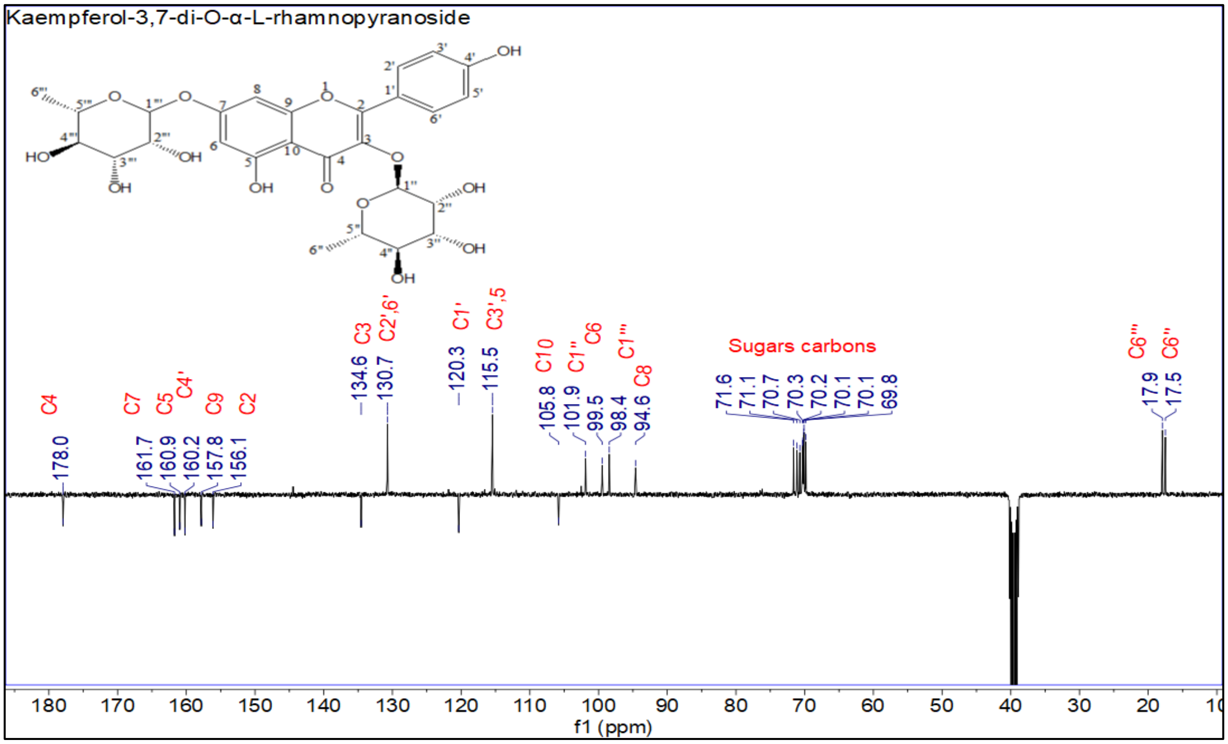

Fig. S56: ^{13}C (APT) NMR spectrum of compound **16** (DMSO-d_6, 100 MHz)

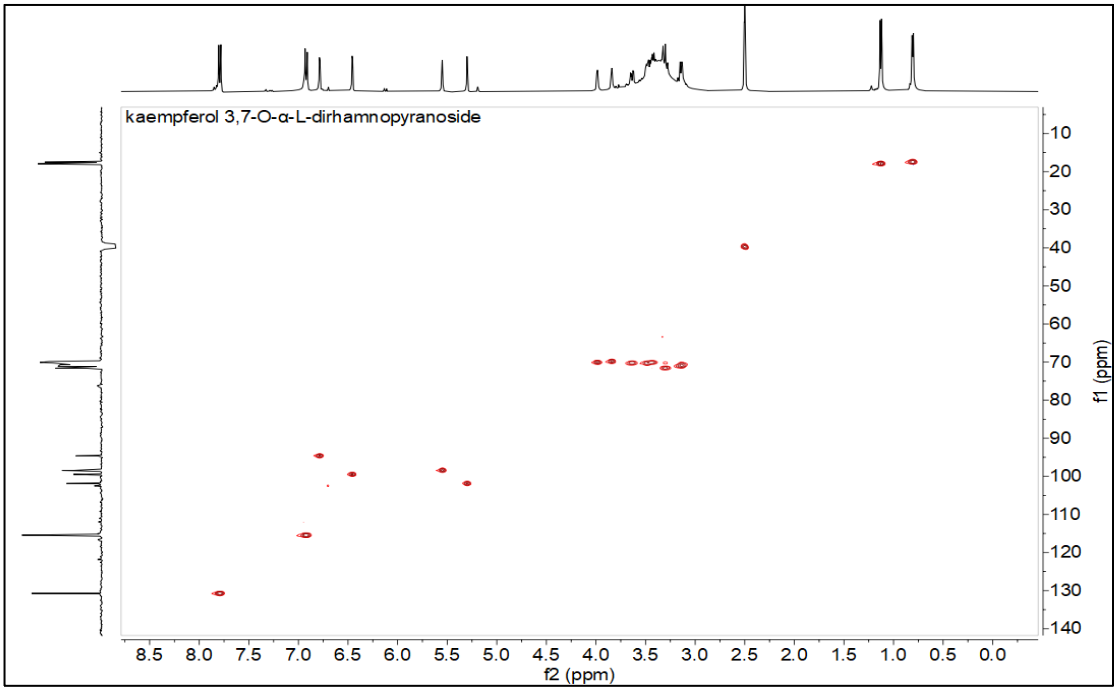

Fig. S57: HSQC spectrum of compound **16** (DMSO-d_6, 400/100 MHz)

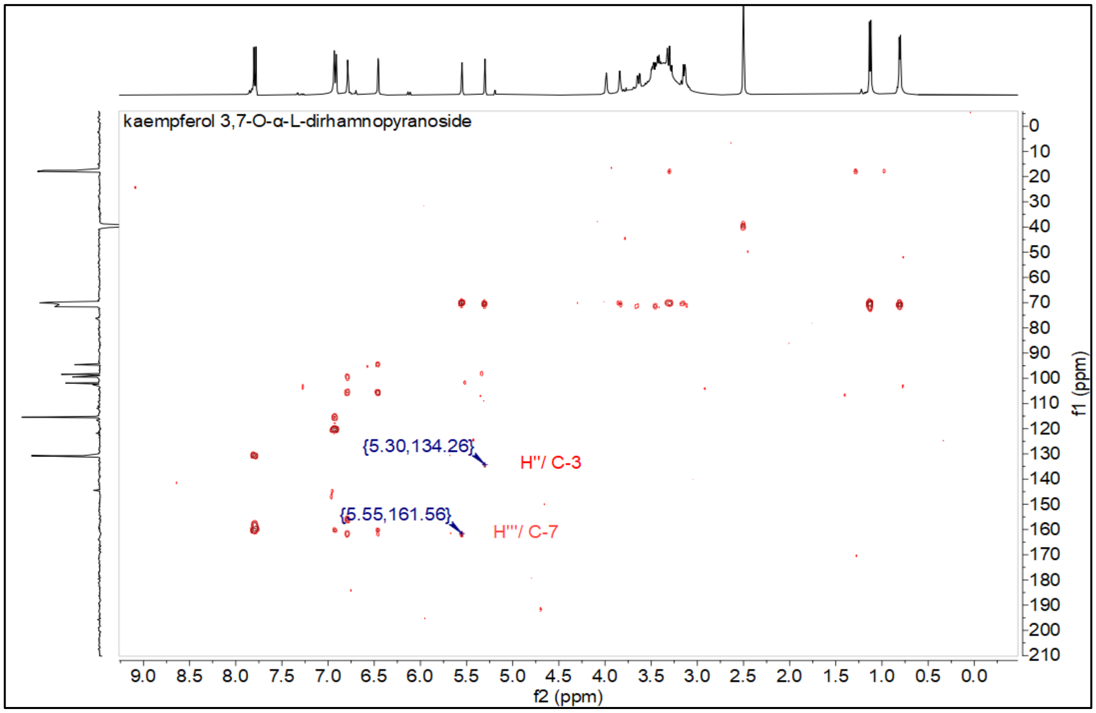

Fig. S58: HMBC spectrum of compound **16** (DMSO-d_6, 400/100 MHz)

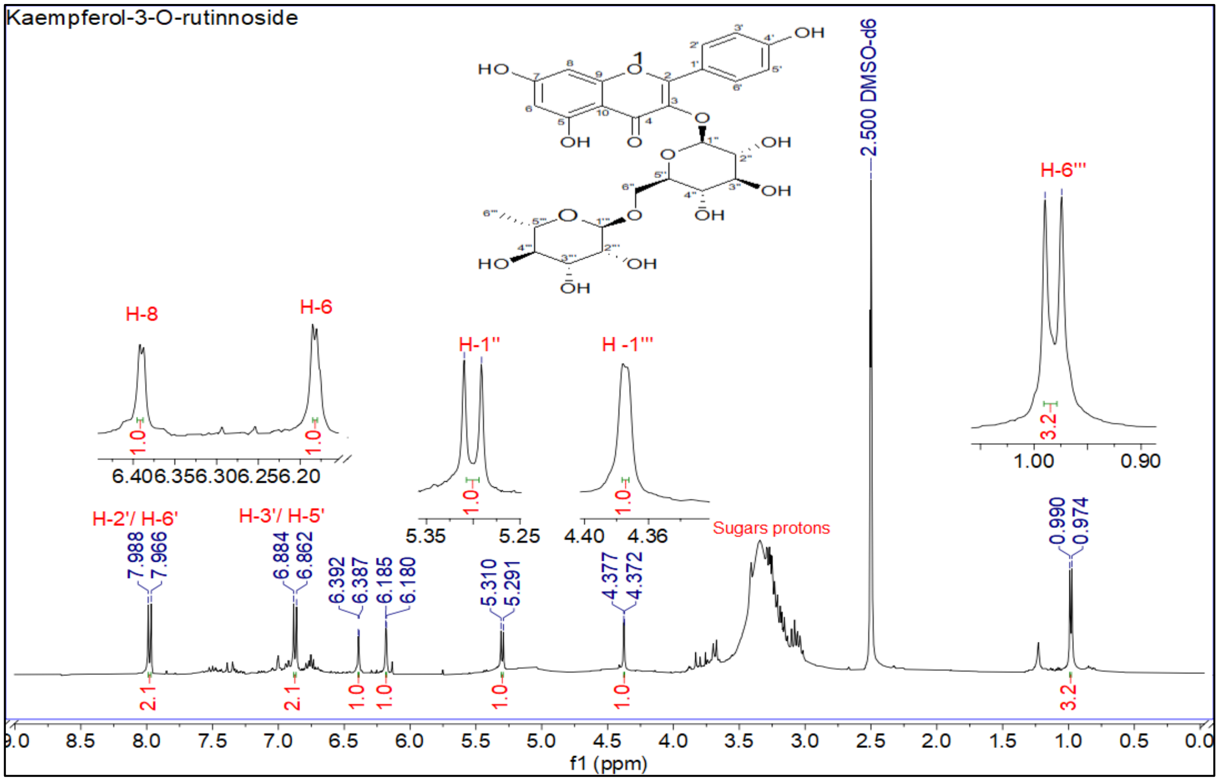

Fig. S59: ^{1}H NMR spectrum of compound **17** (DMSO-d_6, 400 MHz)

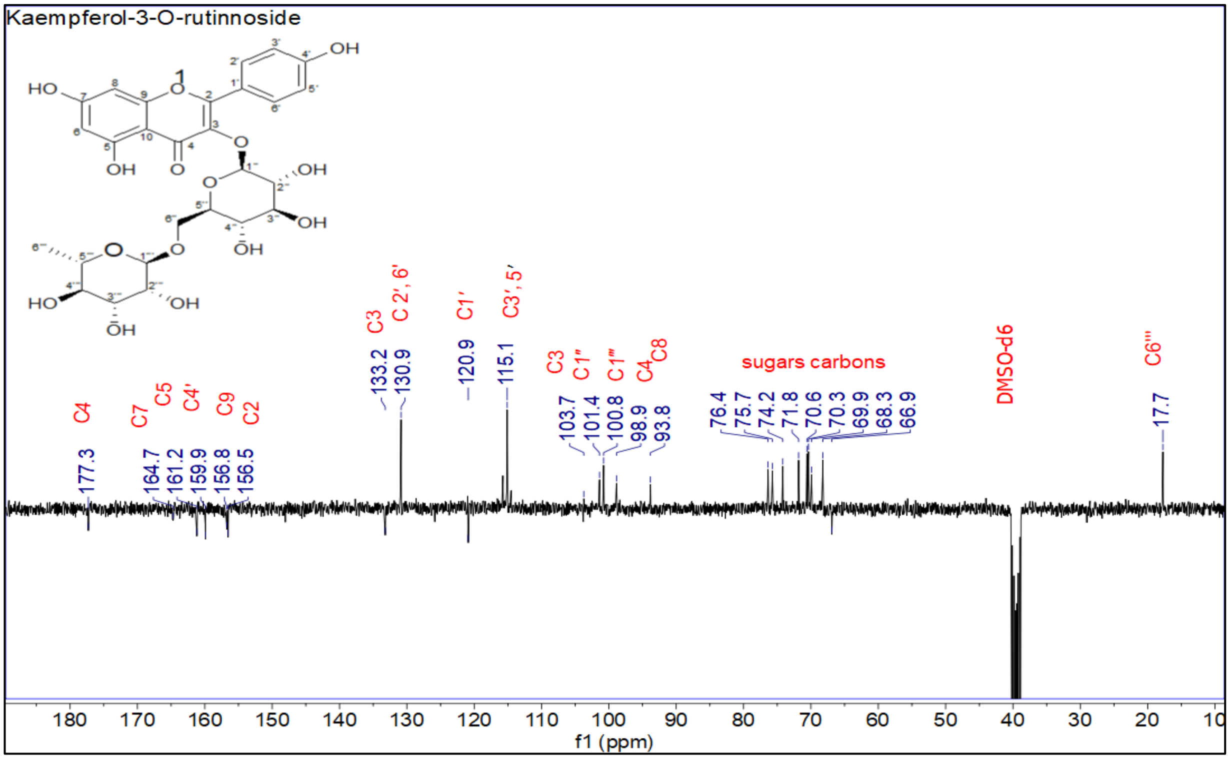

Fig. S60: ^{13}C (APT) NMR spectrum of compound **17** (DMSO-d_6, 100 MHz)

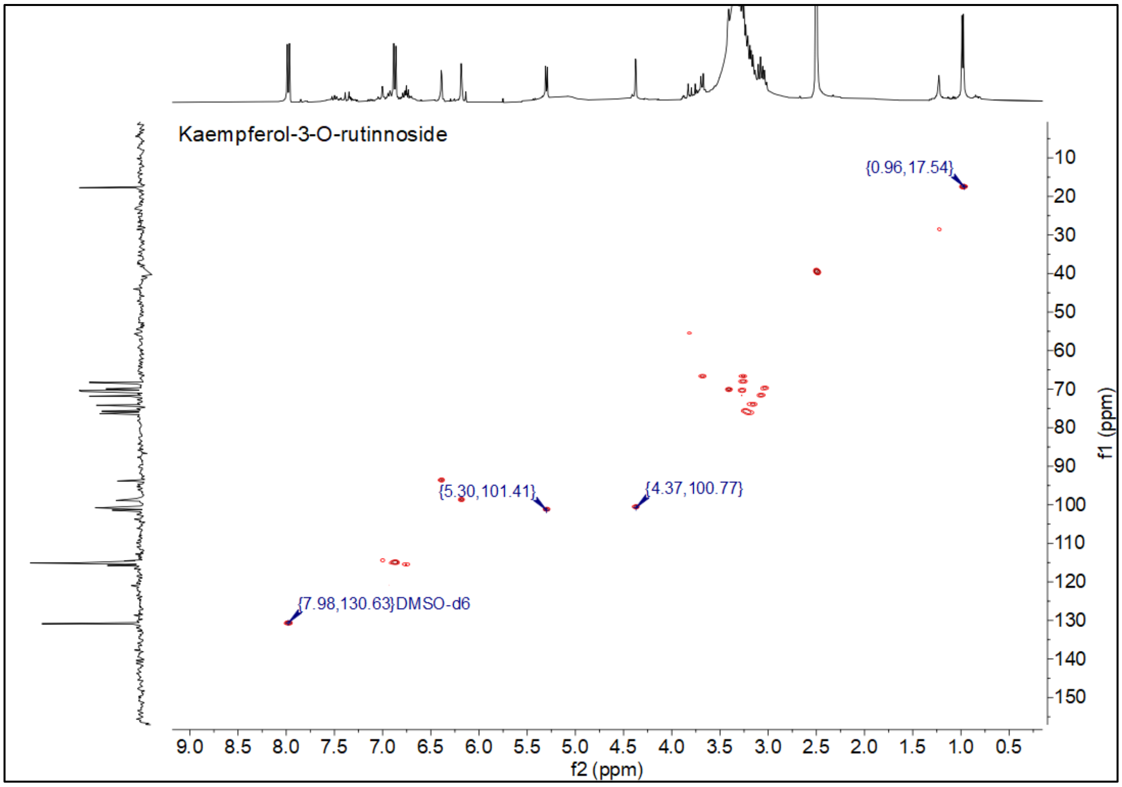

Fig. S61: HSQC spectrum of compound **17** (DMSO-d_6, 400/100 MHz)

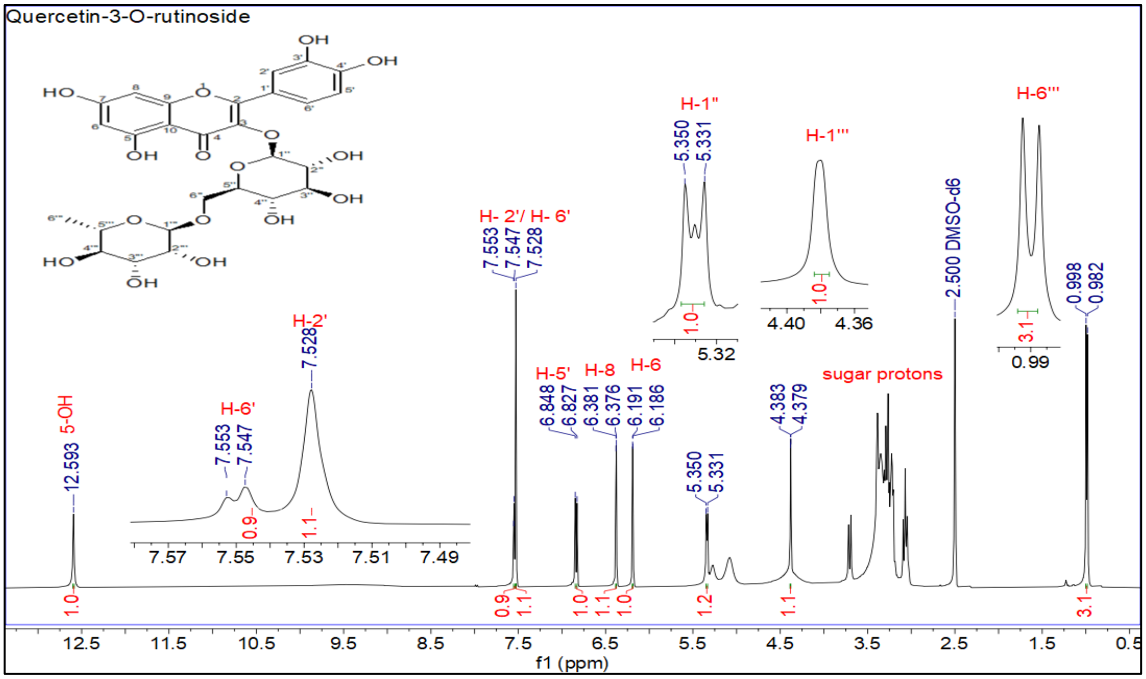

Fig. S62: ^{1}H NMR spectrum of compound **18** (DMSO-d_6, 400 MHz)

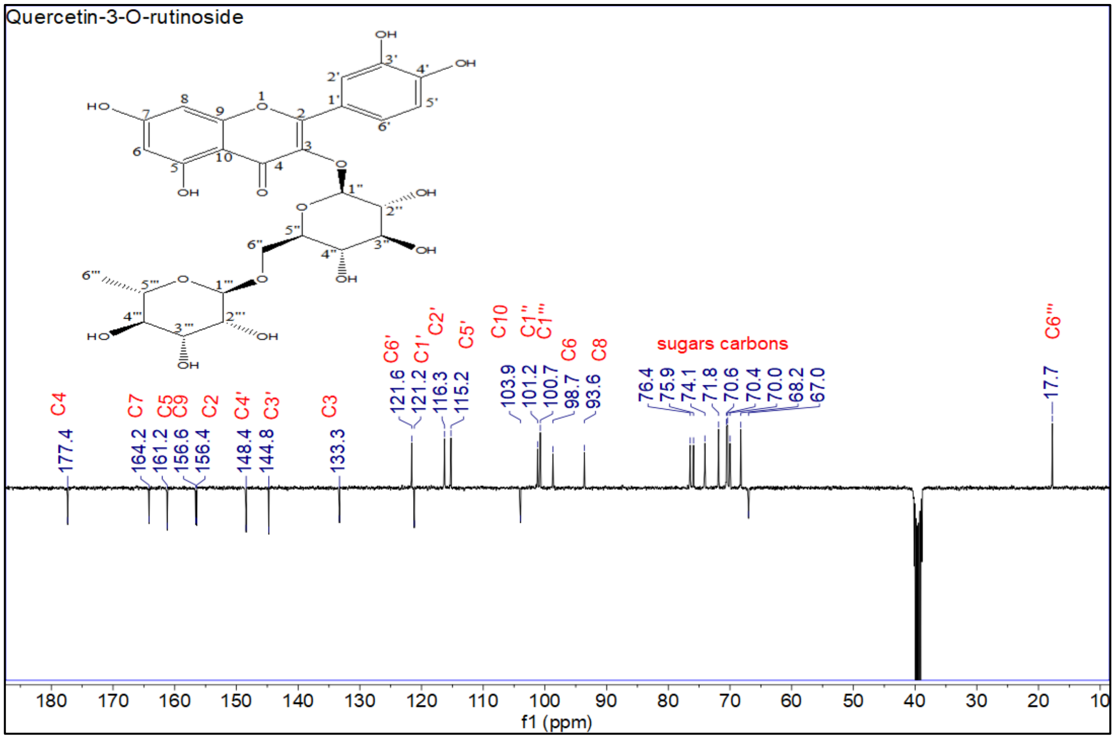

Fig. S63: ^{13}C (APT) NMR spectrum of compound **18** (DMSO-d_6, 100 MHz)

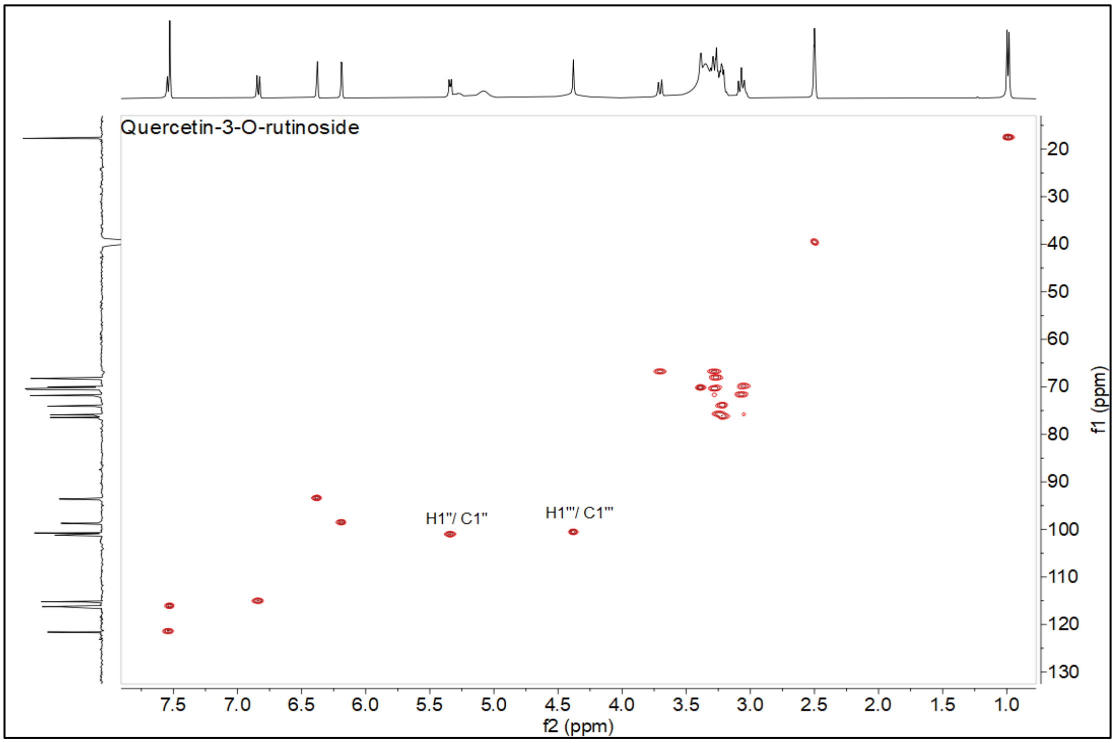

Fig. S64: HSQC spectrum of compound **18** (DMSO-d_6, 400/100 MHz)

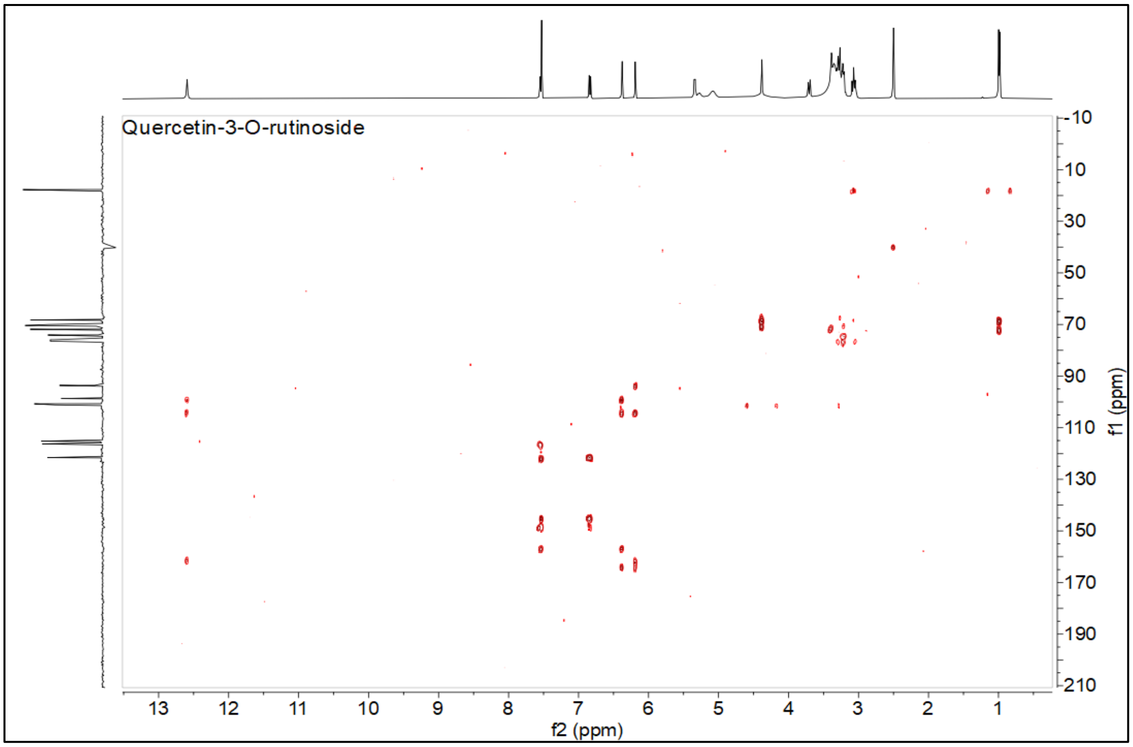

Fig. S65: HMBC spectrum of compound **18** (DMSO-d_6, 400/100 MHz)

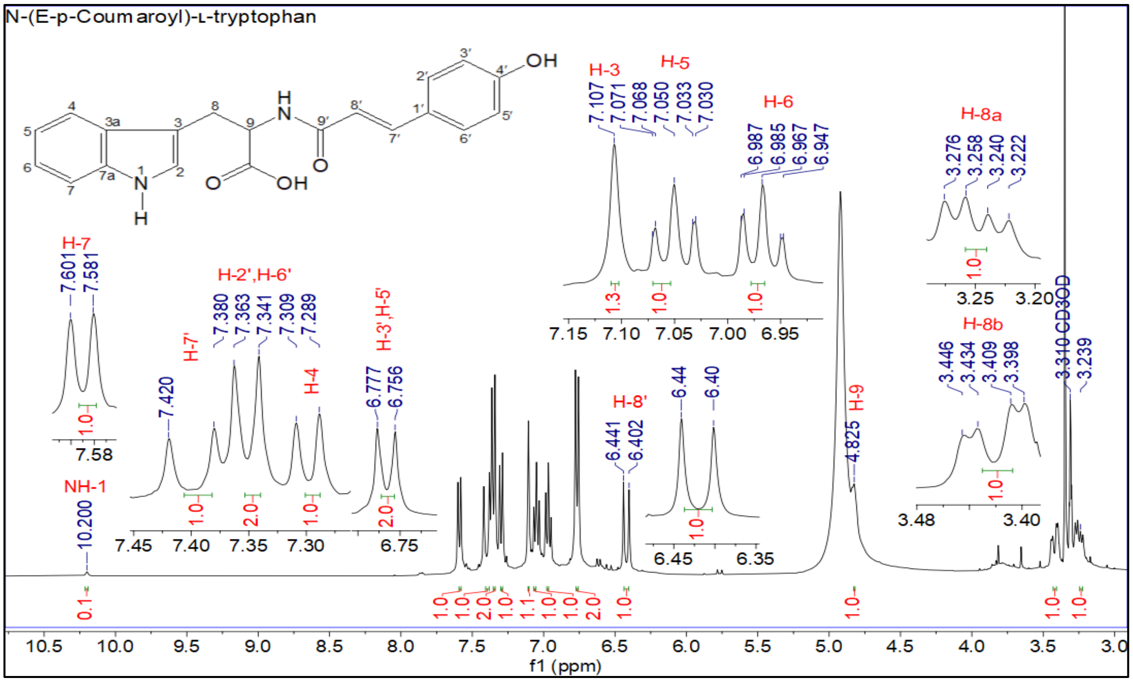

Fig. S66: ^{1}H NMR spectrum of compound **20** (CD_3OD, 400 MHz)

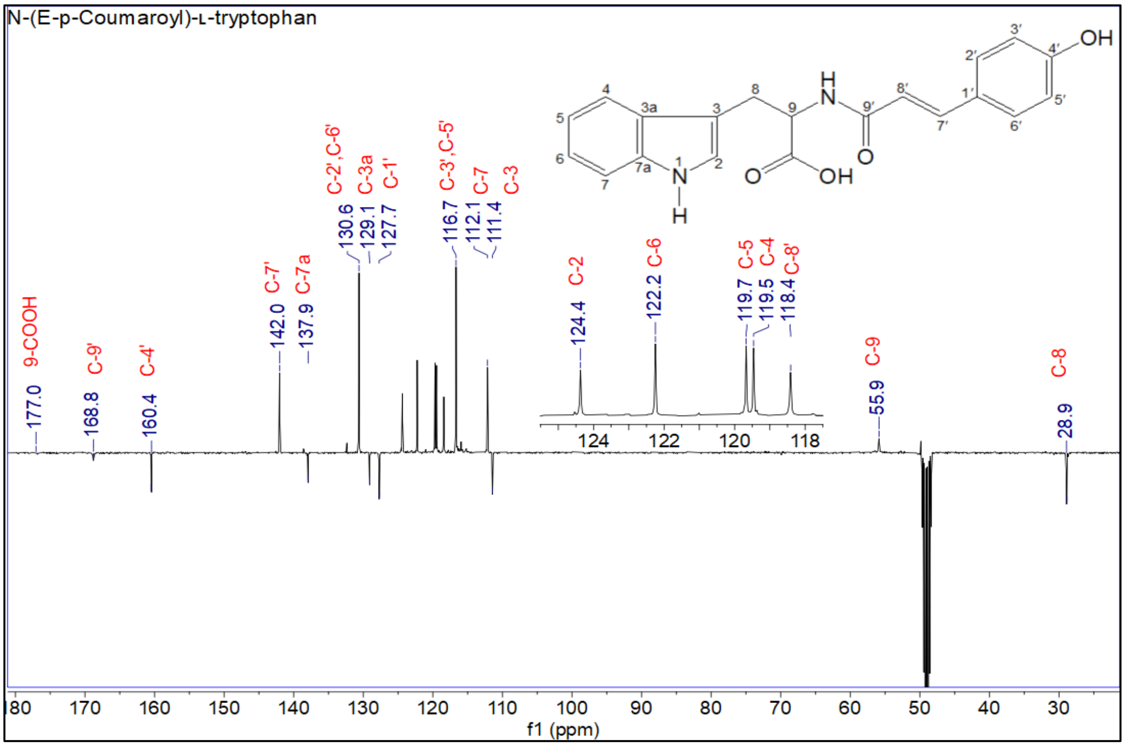

Fig. S67: ^{13}C (APT) NMR spectrum of compound **20** (CD_3OD, 100 MHz)

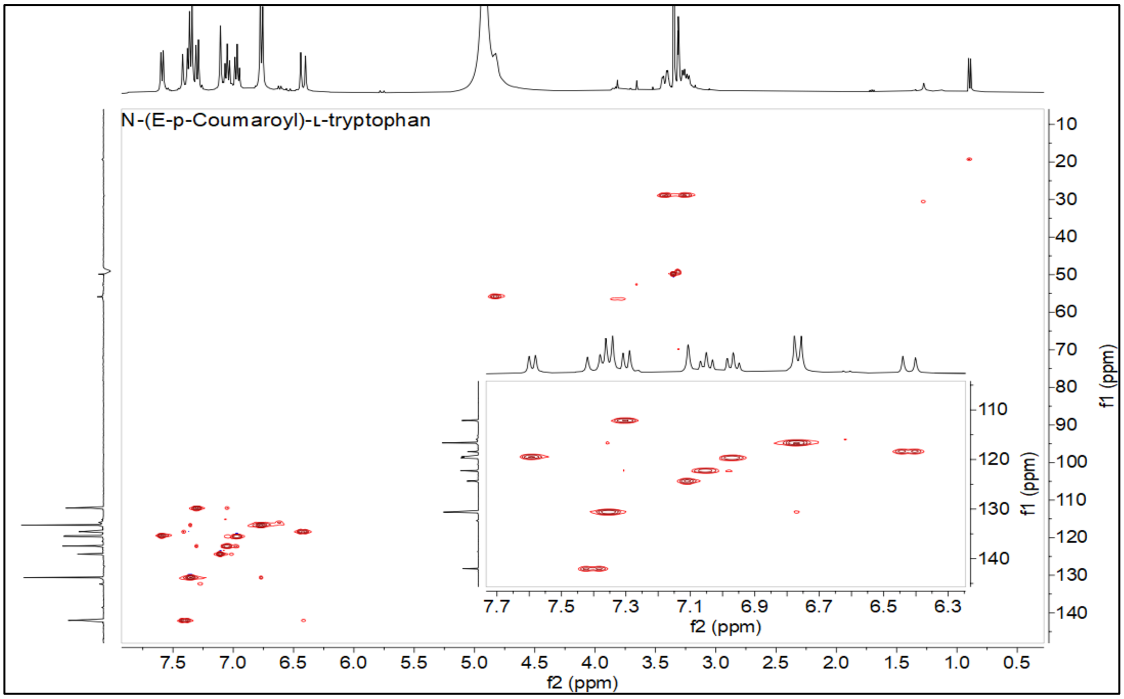

Fig. S68: HSQC spectrum of compound **20** (CD_3OD, 400/100 MHz)

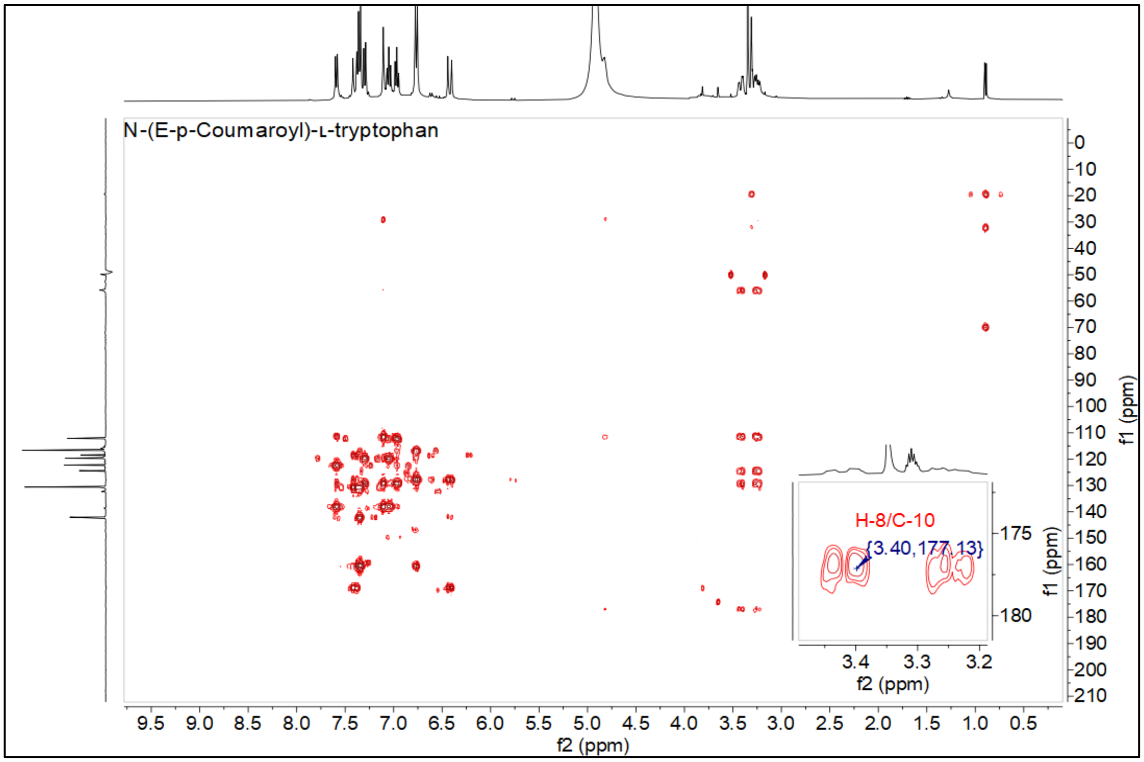

Fig. S69: HMBC spectrum of compound **20** (CD_3OD, 400/100 MHz)

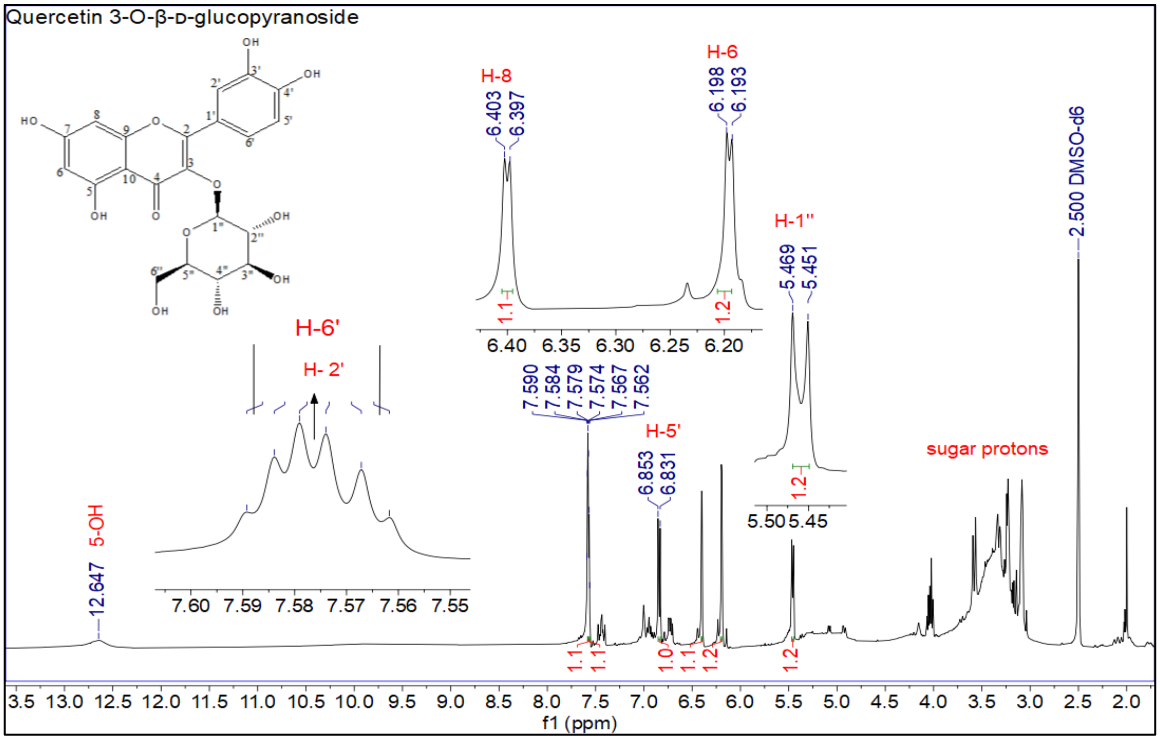

Fig. S70: ^{1}H NMR spectrum of compound **21** (DMSO-d_6, 400 MHz)

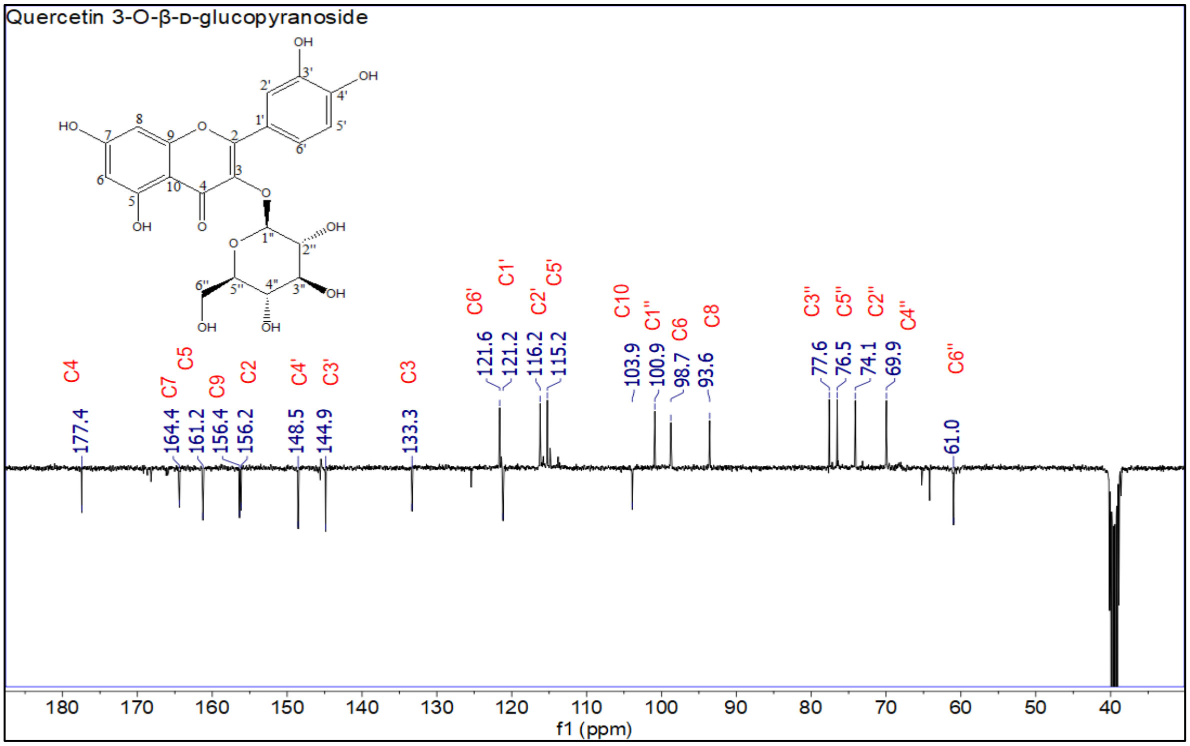

Fig. S71: ^{13}C (APT) NMR spectrum of compound **21** (DMSO-d_6, 100 MHz)

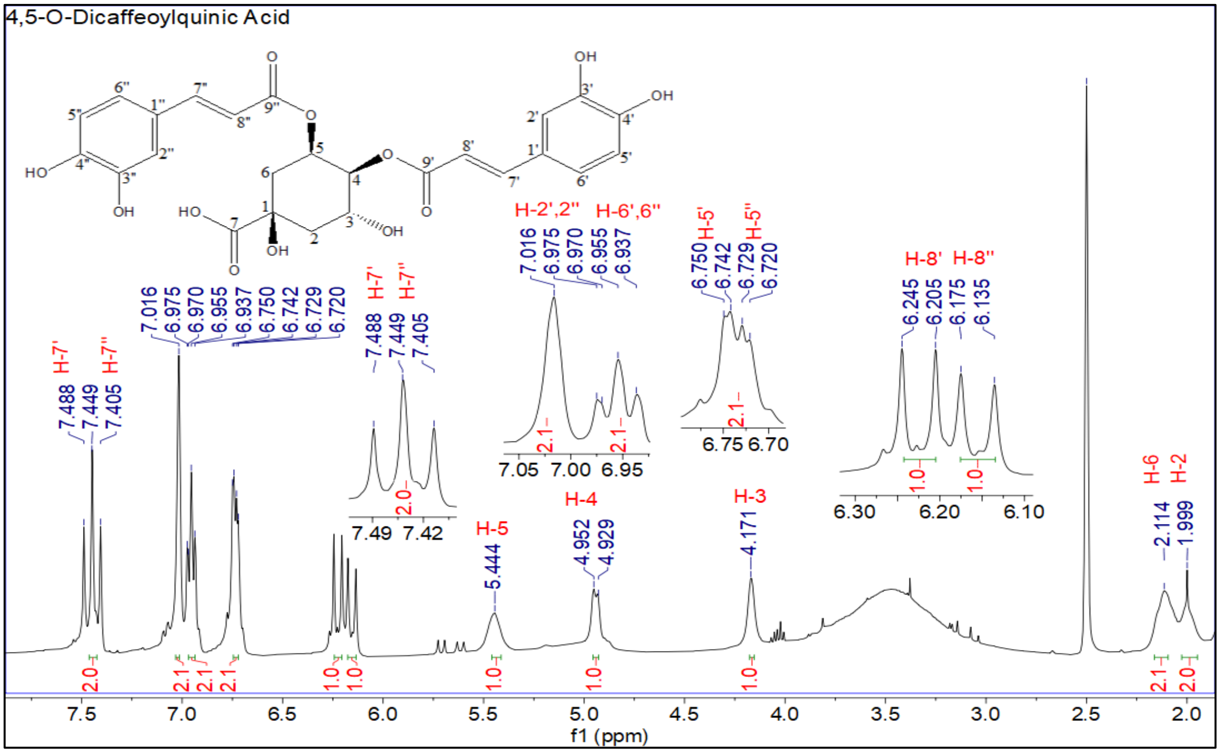

Fig. S72: ^{1}H NMR spectrum of compound **22** (DMSO-d_6, 400 MHz)

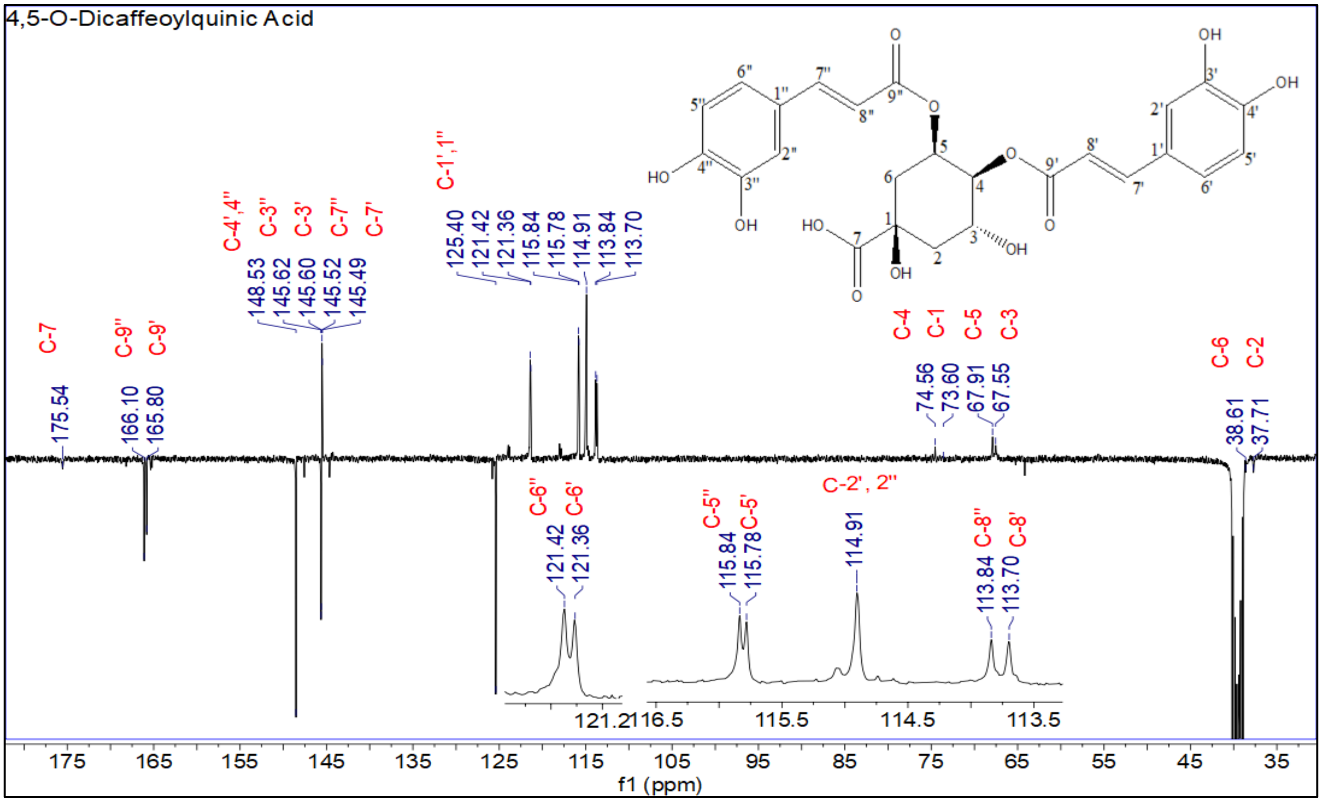

Fig. S73: ^{13}C (APT) NMR spectrum of compound **22** (DMSO-d_6, 400 MHz)

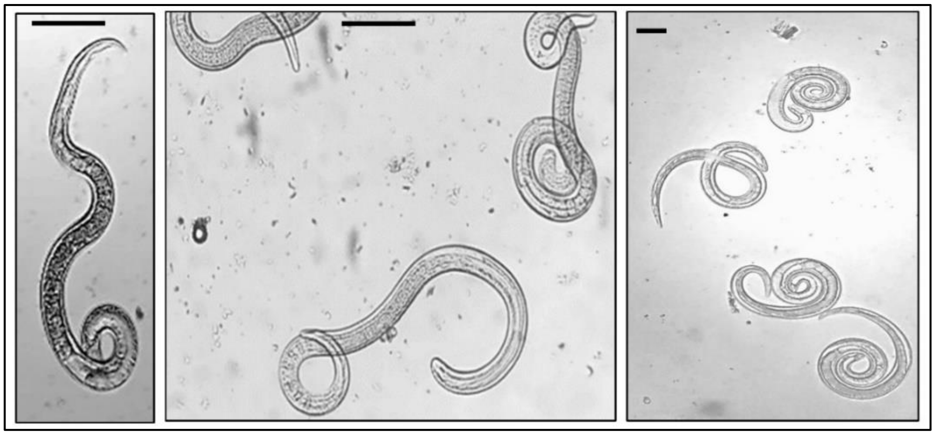

Fig. S74: Microscopic examination of active motile *Trichinella spiralis* larvae, scale bars are 100 μm.

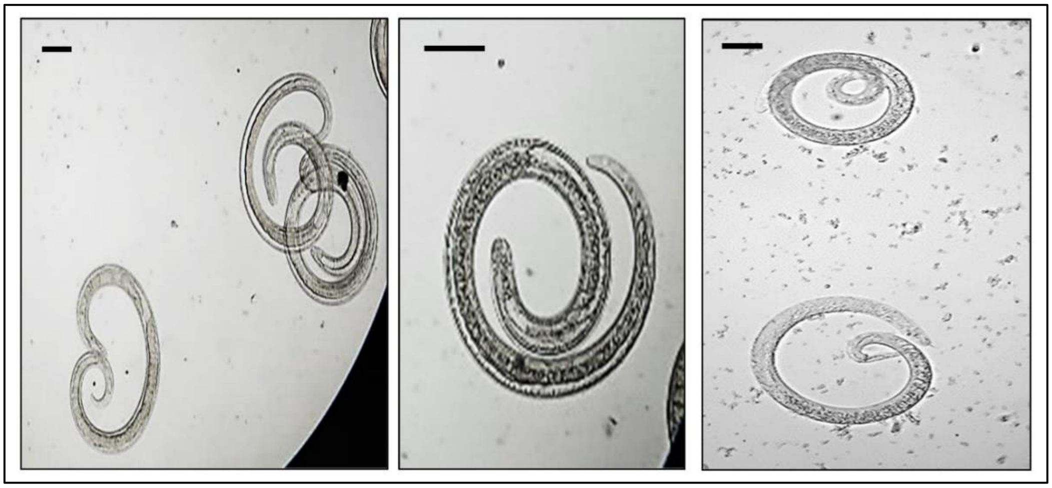

Fig. S75: Microscopic examination of weak coiled *Trichinella spiralis* larvae (wide circle shaped), scale bars are 100 μm.

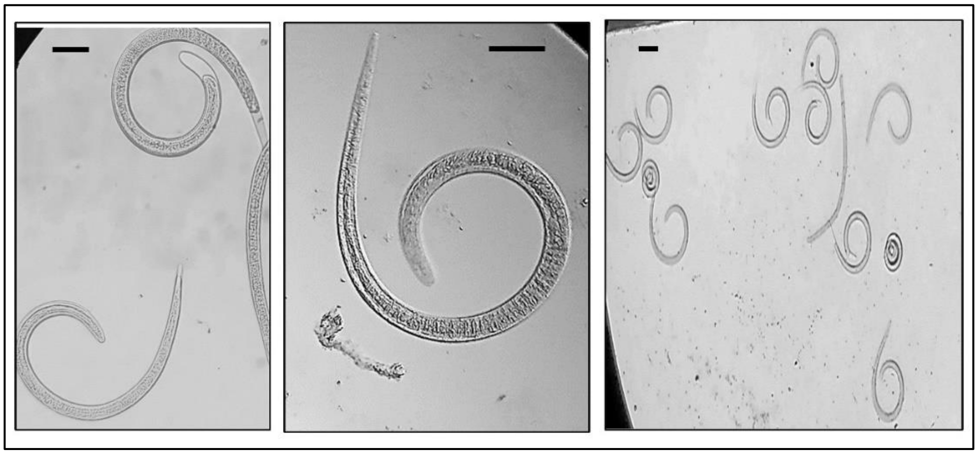

Fig. S76: Microscopic examination of completely dead *Trichinella spiralis* larvae (comma shaped), scale bars are 100 μm.

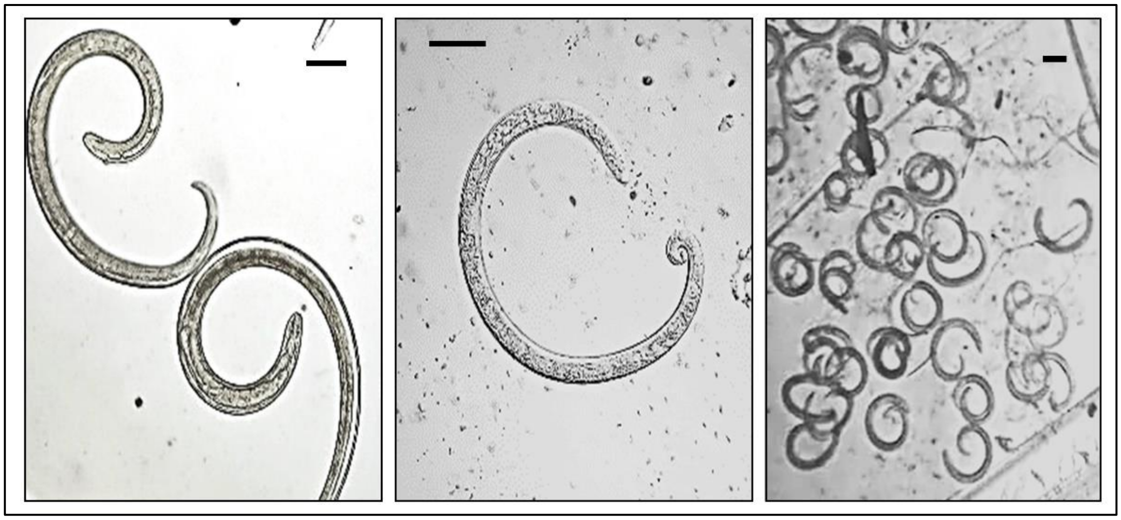

Fig. S77: Microscopic examination of C-shaped *Trichinella spiralis* larvae, scale bars are 100 µm.

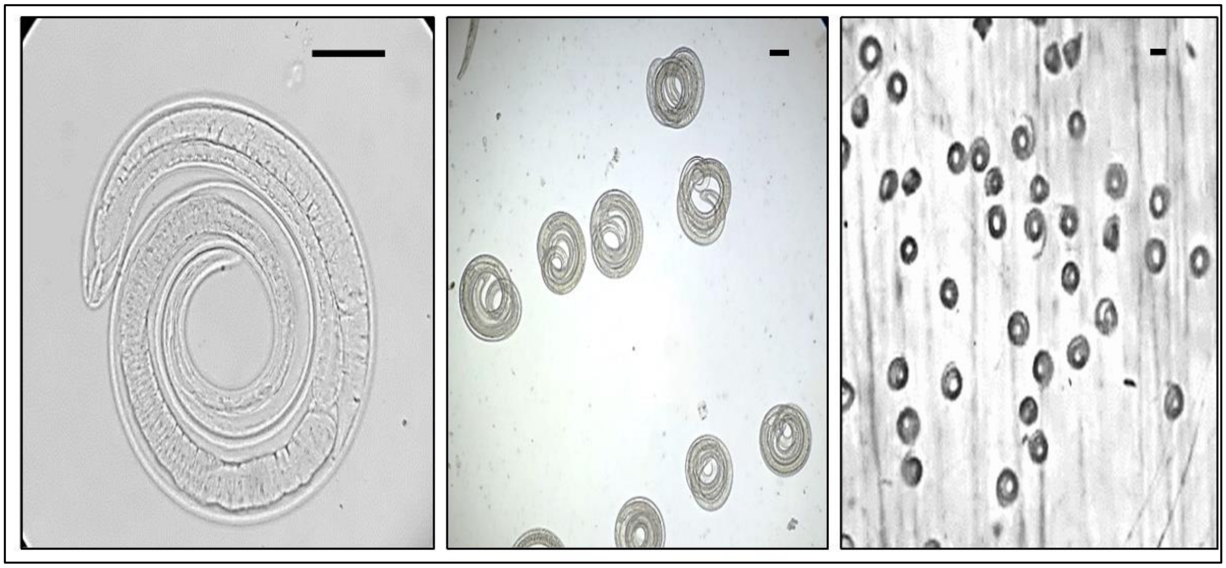

Fig. S78: Microscopic examination of typical supercoiled *Trichinella spiralis* larvae, scale bars are 100 µm.

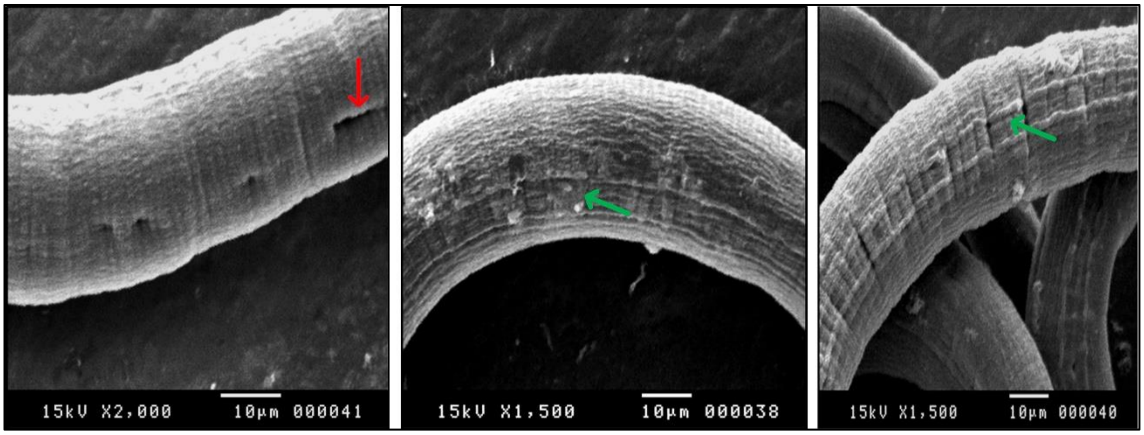

Fig. S79: Scanning electron microscopy of crude ethanolic extract-treated groups, showing opacity and loss of normal striation, pores and holes (red arrow), and blebs (green arrows)

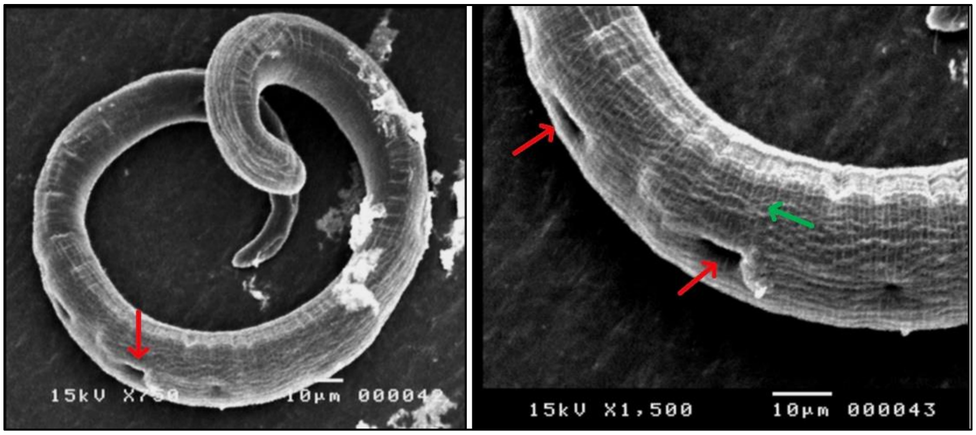

Fig. S80: Scanning electron microscopy of *n*-hexane-treated larvae, showing pores and holes (red arrows), and blebs (green arrow)

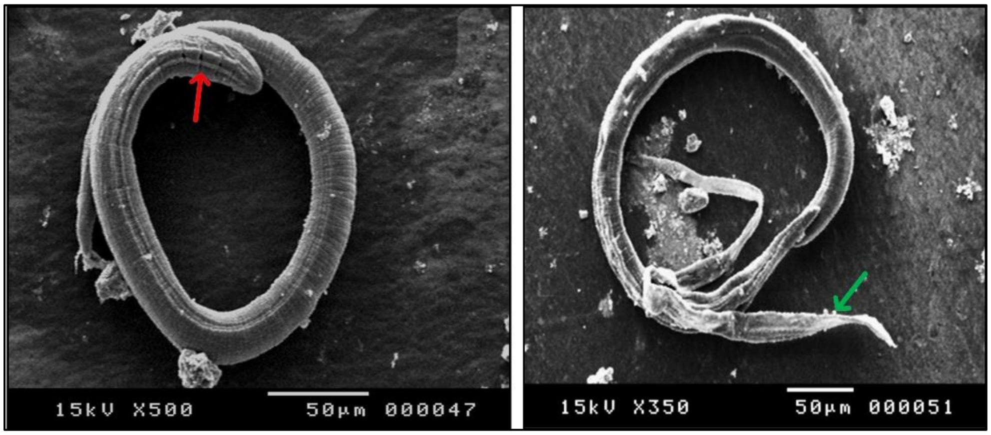

Fig. S81: Scanning electron microscopy of polar fraction-treated larvae, showing opacity shortening, pores and holes (red arrow), sloughing of some areas and detachment of the cuticles (green arrow).
